# NBS-Mediated C(sp^2^)-H Bond Chlorination of Enaminones: Using DCE as Chlorine Source

**DOI:** 10.3390/ijms252212073

**Published:** 2024-11-10

**Authors:** Menglin Peng, Yunhua Xie, Siyu Song, Zhilai Zhang, Yuanzheng Wei, Huimin Hu, Yongchao Wang, Fuchao Yu

**Affiliations:** 1 Faculty of Life Science and Technology, Kunming University of Science and Technology, Kunming 650500, China; 15680220116@163.com (M.P.); xieyunhua2023@163.com (Y.X.); songsiyu083@163.com (S.S.); 20222218046@stu.kust.edu.cn (Z.Z.); 19956087064@163.com (Y.W.); 18760968624@163.com (H.H.); 2 College of Vocational and Technical Education, Yunnan Normal University, Kunming 650092, China

**Keywords:** 1,2-dichloroethane (DCE), enaminones, *α*-chlorinated enaminones, chlorination reaction, NBS-mediated

## Abstract

Commercial DCE is excavated as both a “Cl” source and a solvent for the vinyl C(sp^2^)-H chlorination. The strategy involves a metal-free NBS-mediated C(sp^2^)-H chlorination of enaminones, and affords diverse, functionalized *α*-chlorinated enaminones with a *Z*-configuration. This mild and effective approach not only advances the vinyl C(sp^2^)-H chlorination, employing DCE as the “Cl” source, but also provides a new strategy for accessing chlorinated enaminone derivatives.

## 1. Introduction

**Scheme 1:**
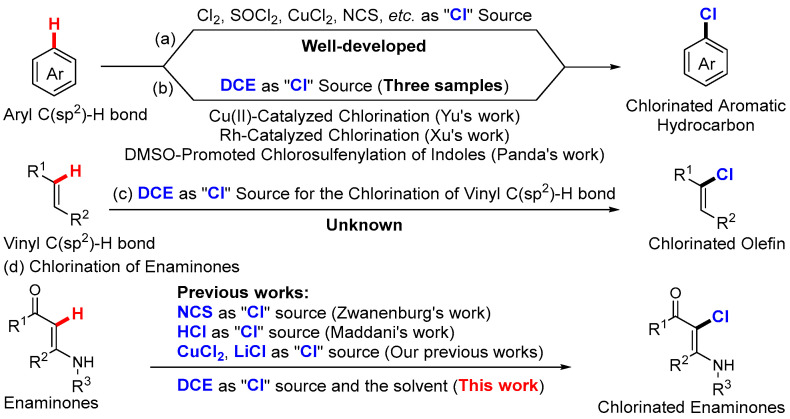
C(sp^2^)-H bond chlorination of enaminones. “C(sp2)-H bond chlorination of enaminones.” Should be changed to “(**a**) Aryl C(sp2)-H bond chlorination using other “Cl” source; (**b**) Aryl C(sp2)-H bond chlorination using DCE as “Cl” source; (**c**) Vinyl C(sp2)-H bond chlorination using using DCE as “Cl” source; (**d**) C(sp2)-H bond chlorination of enaminones.” [16,17,18,33,34].

As a privileged halogen-containing motif, the C(sp^2^)-Cl fragment is ubiquitous, occurring in natural products, synthetically intermediates, pharmaceutical molecules, agricultural chemicals, and functional materials [1,2,3,4]. In recent decades, the direct chlorination of C(sp^2^)-H bond has achieved great success in the formation of the C(sp^2^)-Cl fragment, and a variety of chlorinated reagents, such as Cl_2_, SOCl_2_, CuCl_2_, NCS, etc., have been extensively developed as “Cl” sources, providing efficient protocols to construct many useful organochlorine compounds (Scheme 1a) [5,6,7,8,9]. However, the mentioned well-developed “Cl” sources all rely on the use of external solvents, which may pose some challenges in terms of substrate compatibility and complicated post-processing. As an inexpensive and commercial organic reagent, 1,2-dichloroethane (DCE) has been widely used as a common solvent in organic synthesis. In fact, DCE has also been successfully excavated as an effective “Cl” source to prepare chlorinated aromatic hydrocarbons in the past few decades [10,11,12,13,14,15]. In this concept, DCE was used as a both “Cl” source and a solvent for the C(sp^2^)-H bond conversions, and there are currently three examples available (Scheme 1b). Yu [16] and Xu [17], respectively, describe transition metal-catalyzed regioselective phenyl C(sp^2^)-H bond chlorination, and a DMSO-promoted C(sp^2^)-H bond chlorosulfenylation of indoles has been reported by Panda recently [18]. However, all the abovementioned examples are focused on the aryl C(sp^2^)-H bond chlorination, and to our knowledge, there are no reports on vinyl C(sp^2^)-H bond chlorination by using DCE as a “Cl” source (Scheme 1c).

Enaminones, integrating the structural units of vinyl, carbonyl and amino, have been proved to be reliable organic synthons for the synthesis of valuable heterocycles [19,20,21,22,23,24,25,26,27,28]. Due to the challenge of the vinyl C(sp^2^)-H bond chlorination and the potential biological activities of chlorinated enaminones [29,30,31,32], the C(sp^2^)-H bond chlorination of enaminones has garnered interest (Scheme 1d). In 2002, Zwanenburg presented a regioselective C(sp^2^)-H bond chlorination of enaminones using NCS as a “Cl” source [33]. Subsequently, HCl was employed as a “Cl” source by Maddani for the prepared *α*-halo-*N*-aryl substituted enaminones [34]. Recently, we revealed two catalyzed C(sp^2^)-H bond chlorinations of enaminones by using CuCl_2_ [35] and LiCl [36] as “Cl” sources, respectively, affording a series of highly functionalized *α*-chlorinated enaminones. These methods provide effective strategies for the C(sp^2^)-H bond chlorination of enaminones, but limited by the use of metals, strong acids, and water-sensitive reagents, as well as the need for additional solvents. Following our continuous interest in enaminones’ chemistry [37,38,39,40,41] and vinyl C(sp^2^)-H bond chlorination, we present herein the first metal-free C(sp^2^)-H bond chlorination of enaminones using DCE, both as the “Cl” source and the solvent. This mild approach not only advances the vinyl C(sp^2^)-H chlorination, employing DCE as the “Cl” source, but also provides a new strategy for access to functionalized *α*-chlorinated enaminones.

## 2. Results and Discussion

To establish the feasibility of this NBS-mediated regioselective *α*-chlorination, the enaminone **1a** was selected as the model substrate to optimize the reaction conditions (Table 1). Initially, tetrabutylammonium iodide (TBAI) was tentatively chosen as the catalyst in DCE at 140 °C for 1 h, while it did not produce the desired product (entry 1). Subsequently, TBAC (tetrabutylammonium chloride), NIS (*N*-iodosuccinimide), HBr, PIDA (iodobenzene diacetate), and NBS (*N*-Bromosuccinimide) were sequentially screened (entries 2–6). To our delight, the desired *α*-chlorinated enaminone **2a** was isolated in 58% yield when NBS, which proved compatible, served as a catalyst (entry 6). To improve the reactivity, we then systematically evaluated the solvent effect of this chlorination by specifying NBS as catalyst and DCE as “Cl” source (entries 7–13). All the screened solvents, including both protonic and non-protonic were confirmed to be beneficial for this chlorination, while none of them can provide higher reactivity compared to DCE. We then explored the effect of “Cl” sources on reactivity (entries 13–16), but none of screened chlorinated compounds were better than DCE, and part of them failed to afford the target product (entries 14–15). Optimization efforts, including the reaction time (entries 17–21), catalyst-loading analysis (entries 22–23) and temperature-control experiments (entries 24–25), have been continuously dedicated to the synthesis of *α*-chlorinated enaminone **2a**. The result indicates that appropriately prolonging the reaction time from 1 h to 9 h is beneficial for improving the reactivity (entries 6 and 17–20, from 58% to 90%), while further prolonging the time is detrimental to yield (entry 21, 88%). In addition, when the reaction used a 1.5 equivalent of NBS, the yield dropped sharply and the reaction mixture was complex, probably due to further reactions of α-chlorinated enaminones. The 1.0 equivalent of catalyst loading (entries 22–23) and 140 °C (entries 24–25) proven to be the optimal catalyst dosage and temperature. Overall, the best reaction conditions for the synthesis of *α*-chlorinated enaminone **2a** was achieved by employing enaminone **1a** in DCE at 140 °C for 9 h, in which NBS was used as the promoter.

With the optimal reaction conditions established, we then evaluated the scope of this NBS-mediated α-chlorination. As shown in Table 2, diverse substituents bearing various electron properties and substitution patterns were well tolerated and afforded the corresponding *α*-chlorinated enaminones **2** in moderate-to-excellent yields. The R^2^ substituents of enaminones were evaluated first, and the R^1^ groups were fixed. Specifically, the *N*-aryl substituted enaminones bearing electron-withdrawing (Cl), electroneutral, and electron-donating (Me, OMe) substituents were suitable substrates, affording the corresponding *α*-chlorinated enaminones **2a**–**2d** in excellent reactivity. The *N*-benzyl-substituted enaminones were also compatible with this *α*-chlorination and provided the desired products **2e**–**2f** in 71–76% yields. Additionally, *N*-aliphatic enaminones were also well tolerated, although they only achieved a yield of 40% (**2g**). Then, the R^1^ groups of enaminones were then sequentially fixed as electron-donating OMe and electron-withdrawing Cl, to further evaluate the R^2^ substituents of substrates. Diverse *N*-aryl-, *N*-benzyl-, *N*-cyclohexyl-, and *N*-pyridyl-substituted enaminones were applicable and afforded the corresponding products **2h**–**2o** in 67–93% yields. Subsequently, the R^1^ groups of enaminones have also been systematically evaluated. We were pleased to find this NBS-mediated regioselective *α*-chlorination was tolerated for all tested enaminones **1** bearing various different R^1^ substituents. Notably, the electron-withdrawing substituents (Br, NO_2_) and *para*-substitution (**2q**–**2s**) were more favorable for the reaction compared to *ortho*-substitution (**2p**) and phenyl group (**2t**). The naphthyl- and pyridyl-containing enaminones were also suitable substrates, affording the chlorinated enaminones **2u**–**2w** in 85–92% yields. In addition, the cyclic enaminones were also investigated, and diverse cyclic substrates were shown to be well accommodated. Delightfully, this regioselective *α*-chlorination could also apply to 5-amino-3-methyl-1-phenylpyrazole, providing the desired product **2b’** in 92% yield. The structure of products with *Z*-configurations was unequivocally established by employing **2m** as a representative example via X-ray crystallographic analysis (CCDC 2214194).

**Scheme 2:**
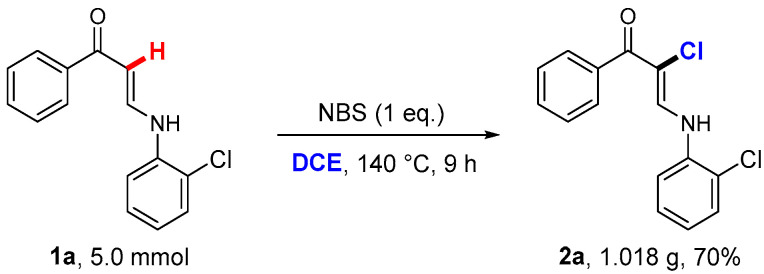
Gram-level reaction.

The synthetic potential of this NBS-mediated *α*-chlorination was tentatively demonstrated by the gram-scale preparation of compound **2a** (Scheme 2). Under the optimal reactions, this reaction proceeded smoothly at 5.0 mmol scale and afforded the products in 70% yield (1.018 g).

**Scheme 3:**
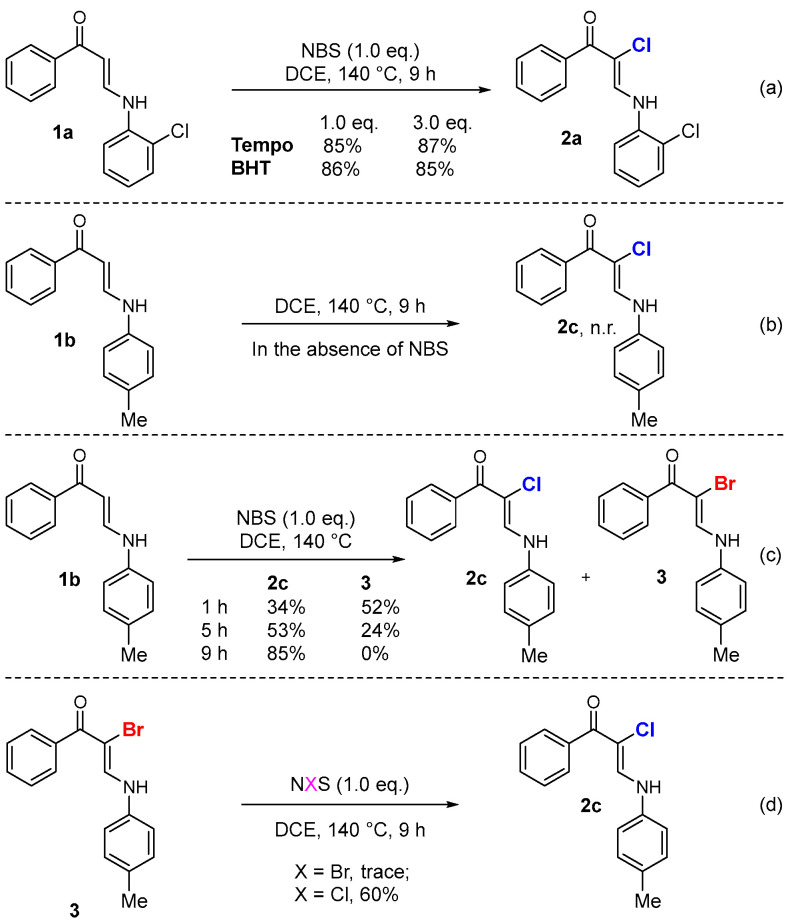
Control experiments. Control experiments.” Should be changed to ”(**a**) Radical-inhibition experiments; (**b**) Necessity experiments of NBS; (**c**) Time-control and intermediate-capture experiments; (**d**) Intermediate validation experiments.

To gain insight into the mechanism of this NBS-mediated *α*-chlorination, some control experiments were performed (Scheme 3). Firstly, the radical-inhibition experiment was conducted in the presence of 2,2,6,6-tetramethylpiperidinooxy (TEMPO) and butylated hydroxytoluene (BHT) under standard conditions. As indicated by the results presented in Scheme 3a, there was no significant change in reactivity when a 1.0 or 3.0 equivalent of free radical inhibitors was sequentially employed, which may suggest that this transformation might not involve a radical process. To clarify the necessity of the NBS, this *α*-chlorination reaction was conducted in the absence of NBS, and we failed to detect the desired product **2c** (Scheme 3b). By controlling the reaction time, α-chlorinated enaminone **2c** and α-brominated enaminone **3** could be isolated, respectively, under standard conditions (Scheme 3c). The results showed that the yield of α-chlorinated enaminone **2c** increased gradually with increasing time, while α-brominated enaminone **3** showed a decreasing trend. In addition, when α-brominated enaminone **3** reacted with NCS under standard conditions, α-chlorinated enaminone **2c** could be obtained in 60% yield, but the reaction could not be smoothly transformed with NBS (Scheme 3d). These results indicated that α-brominated enaminones and NCS may be the important intermediates in this α-chlorination reaction.

**Scheme 4:**
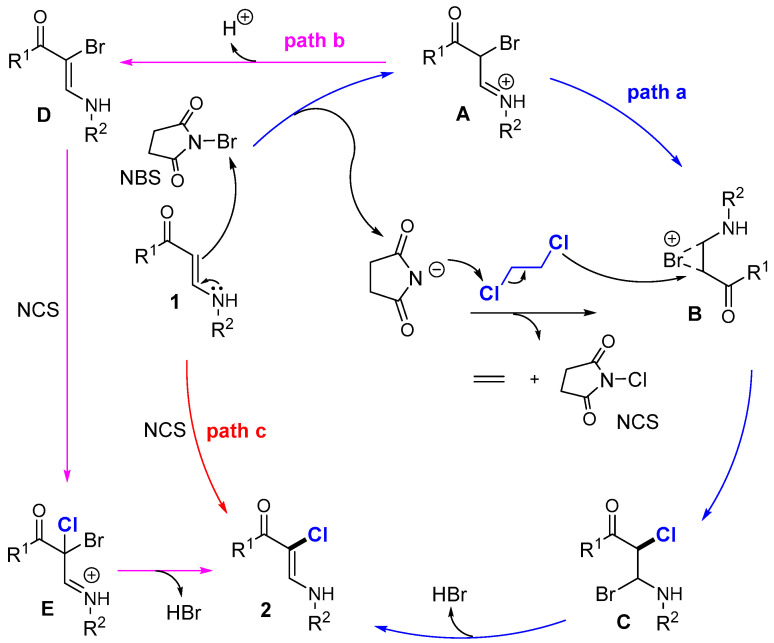
Proposed reaction mechanism.

Based on the above experimental results, a preliminary mechanism for this NBS-mediated *α*-chlorination reaction was proposed, as shown in Scheme 4. First, enaminone **1** reacts with NBS to form a succinimide anion and intermediate **A**, which then forms intermediate bromonium ion **B**. Meanwhile, the succinimide anion attacks a chlorine atom of DCE, causing another chlorine atom to attack intermediate bromonium ion **B** to form intermediate **C**, NCS, and ethylene. Finally, the desired product **2** is obtained via the subsequent dehydrobromination process of **C** (path a). Furthermore, the other two alternative mechanisms are the direct chlorination and elimination process of intermediate **A** or enaminone **1** by the in situ formed NCS to produce the desired product **2** (paths b and c).

## 3. Materials and Methods

### 3.1. General Information 

All chemicals and solvents were used as received without further purification unless otherwise stated. Column chromatography was performed on silica gel (200–300 mesh). All compounds were fully characterized by spectroscopic data. The NMR spectra were recorded on a DRX600 (^1^H: 500 MHz and 600 MHz, ^13^C: 125 MHz and 150 MHz), chemical shifts (*δ*) are expressed in ppm, *J* values are given in Hz, and deuterated CDCl_3_ and DMSO-*d_6_* were used as solvents. The reactions were monitored by thin-layer chromatography (TLC) using silica gel GF_254_. The melting points were determined on a XT-4A melting point apparatus and are uncorrected. HRMs were performed on an Agilent LC/MS TOF instrument.

*N*-Aryl enaminone **2** were prepared according to the previous literature [42,43,44]. Other reagents were purchased from Energy Chemical and Adamas-beta^®^. The X-ray Structure and Data of **2m**, ^1^H, ^13^C NMR and HRMS spectras for compounds **2** were included in Supplementary Materials.

### 3.2. General Procedure for the Synthesis of α-Chlorinated Enaminone ***2***

Enaminone 1 (0.5 mmol), NBS (0.5 mmol), and DCE (2 mL) were placed into 15 mL Ace Glass pressure tubes, and the mixture was stirred at 140 °C for 9.0 h until the enaminones were completely consumed. The mixture was cooled to room temperature, and then EtOAc (15 mL × 2) were added. The organic phase was washed with water (10 mL), dried over Na_2_SO_4_, and then concentrated and purified by flash column chromatography to afford the halogenation of *N*-aryl enaminone **2**.

**(*Z*)-2-Chloro-1-phenyl-3-(phenylamino)prop-2-en-1-one (2a).** V_Petroleum ether_/V_Ethyl acetate_ = 20:1, R*_f_* = 0.2; Yellow solid: 131 mg (90%); mp = 130–132 °C; ^1^H NMR (600 MHz, CDCl_3_): *δ* = 7.85 (d, *J* = 13.1 Hz, 1H, C=CH), 7.69 (d, *J* = 13.0 Hz, 1H, NH), 7.64 (d, *J* = 7.5 Hz, 2H, ArH), 7.56–7.54 (m, 1H, ArH), 7.49–7.47 (m, 2H, ArH), 7.41 (d, *J* = 8.1 Hz, 1H, ArH), 7.22 (t, *J* = 7.8 Hz, 1H, ArH), 7.02–6.99 (m, 1H, ArH), 6.90 (d, *J* = 8.2 Hz, 1H, ArH); ^13^C NMR (150 MHz, CDCl_3_): *δ* = 188.3, 139.7, 138.6, 135.9, 131.4, 130.3, 128.7, 128.7, 128.6, 128.6, 128.4, 124.2, 122.6, 114.8, 111.6; HRMS (TOF ESI^+^): *m*/*z* calcd for C_15_H_12_Cl_2_NO^+^ [(M+H)^+^], 292.0290, found, 292.0284.

**(*Z*)-2-Chloro-1-phenyl-3-(phenylamino)prop-2-en-1-one (2b)**. V_Petroleum ether_/V_Ethyl acetate_ = 10:1, R*_f_* = 0.2; Yellow solid: 123 mg (96%); mp = 189–191 °C; ^1^H NMR (600 MHz, CDCl_3_): *δ* = 7.85 (d, *J* = 13.2 Hz, 1H, C=CH), 7.62 (d, *J* = 6.9 Hz, 2H, ArH), 7.55–7.52 (m, 1H, ArH), 7.48–7.46 (m, 2H, ArH), 7.33–7.30 (m, 2H, ArH), 7.20 (d, *J* = 13.2 Hz, 1H, NH), 7.10–7.07 (m, 1H, ArH), 6.92 (d, *J* = 8.0 Hz, 2H, ArH); ^13^C NMR (150 MHz, CDCl_3_): *δ* = 188.3, 141.4, 139.2, 138.8, 131.2, 130.1, 130.1, 128.6, 128.6, 128.6, 128.6, 124.3, 116.4, 116.4, 109.9; HRMS (TOF ESI^+^): *m*/*z* calcd for C_15_H_13_ClNO^+^ [(M+H)^+^], 258.0680, found, 258.0678.

**(*Z*)-2-Chloro-1-phenyl-3-(*p*-tolylamino)prop-2-en-1-one (2c).** V_Petroleum ether_/V_Ethyl acetate_ = 7:1, R*_f_* = 0.2; Yellow solid: 109 mg (80%); mp = 168–170 °C; ^1^H NMR (600 MHz, CDCl_3_): *δ* = 7.81 (d, *J* = 13.3 Hz, 1H, C=CH), 7.62–7.60 (m, 2H, ArH), 7.53–7.51 (m, 1H, ArH), 7.47–7.45 (m, 2H, ArH), 7.15 (d, *J* = 12.8 Hz, 1H, NH), 7.11 (d, *J* = 8.0 Hz, 2H, ArH), 6.82 (d, *J* = 8.1 Hz, 2H, ArH), 2.30 (s, 3H, ArCH_3_); ^13^C NMR (150 MHz, CDCl_3_): *δ* = 185.7, 139.4, 136.5, 134.3, 131.6, 128.6, 128.1, 128.1, 126.2, 126.2, 126.1, 126.1, 114.1, 114.1, 106.9, 18.4; HRMS (TOF ESI^+^): *m*/*z* calcd for C_16_H_15_ClNO^+^ [(M+H)^+^], 272.0837, found, 272.0840.

**(*Z*)-2-Chloro-3-((4-methoxyphenyl)amino)-1-phenylprop-2-en-1-one (2d).** V_Petroleum ether_/V_Ethyl acetate_ = 10:1, R*_f_* = 0.2; Yellow solid: 84 mg (74%); mp = 113–114 °C; ^1^H NMR (600 MHz, CDCl_3_): *δ* = 7.75 (d, *J* = 13.3 Hz, 1H, C=CH), 7.60 (d, *J* = 6.9 Hz, 2H, ArH), 7.52–7.50 (m, 1H, ArH), 7.46–7.44 (m, 2H, ArH), 7.13 (d, *J* = 13.2 Hz, 1H, NH), 6.89–6.84 (m, 4H, ArH), 3.78 (s, 3H, ArOCH_3_); ^13^C NMR (150 MHz, CDCl_3_): *δ* = 188.1, 156.7, 142.6, 139.0, 132.7, 131.0, 128.6, 128.6, 128.6, 128.6, 118.3, 118.3, 115.2, 115.2, 109.0, 55.7; HRMS (TOF ESI^+^): *m*/*z* calcd for C_16_H_15_ClNO_2_^+^ [(M+H)^+^], 228.0786, found, 228.0784.

**(*Z*)-2-Chloro-3-((4-methoxybenzyl)amino)-1-phenylprop-2-en-1-one (2e).** V_Petroleum ether_/V_Ethyl acetate_ = 20:1, R*_f_* = 0.1; Yellow solid: 107 mg (71%); mp = 116–117 °C; ^1^H NMR (600 MHz, CDCl_3_): *δ* = 7.50–7.48 (m, 2H, ArH), 7.46–7.44 (m, 1H, ArH), 7.40–7.37 (m, 2H, ArH), 7.34 (d, *J* = 13.5 Hz, 1H, C=CH), 7.15 (d, *J* = 8.5 Hz, 2H, ArH), 6.91–6.89 (m, 2H, ArH), 5.64–5.59 (m, 1H, NH), 4.36 (d, *J* = 5.8 Hz, 2H, ArCH_2_), 3.81 (s, 3H, ArOCH_3_); ^13^C NMR (150 MHz, CDCl_3_): *δ* = 187.7, 159.7, 149.7, 139.4, 130.5, 128.9, 128.9, 128.9, 128.4, 128.4, 128.4, 128.4, 114.5, 114.5, 106.4, 55.5, 51.6; HRMS (TOF ESI^+^): *m*/*z* calcd for C_17_H_17_ClNO_2_^+^ [(M+H)^+^], 302.0942, found, 302.0952.

**(*Z*)-2-Chloro-3-((1-(naphthalen-2-yl)ethyl)amino)-1-phenylprop-2-en-1-one (2f).** V_Petroleum ether_/V_Ethyl acetate_ = 20:1, R*_f_* = 0.1; Yellow solid: 127 mg (76%); mp = 123–124 °C; ^1^H NMR (600 MHz, CDCl_3_): *δ* = 7.94–7.91 (m, 2H, ArH), 7.83 (d, *J* = 8.1 Hz, 1H, ArH), 7.58–7.53 (m, 2H, ArH), 7.49–7.47 (m, 1H, ArH), 7.43 (d, *J* = 7.1 Hz, 1H, C=CH), 7.37–7.32 (m, 3H, ArH), 7.28 (s, 1H, ArH), 7.25–7.23 (m, 2H, ArH), 5.80–5.77 (m, 1H, NH), 5.34–5.30 (m, 1H, CH), 1.74 (d, *J* = 6.8 Hz, 3H, CH_3_); ^13^C NMR (150 MHz, CDCl_3_): *δ* = 187.7, 148.3, 139.1, 137.4, 134.1, 130.5, 130.3, 129.4, 129.0, 128.4, 128.4, 128.2, 128.2, 126.9, 126.2, 125.6, 123.2, 122.5, 106.7, 53.4, 22.9; HRMS (TOF ESI^+^): *m*/*z* calcd for C_21_H_19_ClNO^+^ [(M+H)^+^], 336.1150, found, 336.1162.

**(*Z*)-2-Chloro-1-phenyl-3-((4-phenylbutan-2-yl)amino)prop-2-en-1-one (2g).** V_Petroleum ether_/V_Ethyl acetate_ = 8:1, R*_f_* = 0.2; White solid: 72 mg (46%); mp = 98–100 °C; ^1^H NMR (600 MHz, CDCl_3_): *δ* = 7.51 (d, *J* = 7.2 Hz, 2H, ArH), 7.48–7.40 (m, 4H, ArH), 7.25 (d, *J* = 8.5 Hz, 2H, ArH), 7.20 (d, *J* = 7.3 Hz, 1H, C=CH), 7.10 (d, *J* = 7.4 Hz, 2H, ArH), 5.25–5.22 (m, 1H, NH), 3.29–3.24 (m, 1H, CH), 2.73–2.69 (m, 5.7 Hz, 1H, CH_2_), 2.62–2.59 (m, 1H, CH_2_), 1.89–1.82 (m, 2H, CH_2_), 1.25 (d, *J* = 6.3 Hz, 3H, CH_3_); ^13^C NMR (150 MHz, CDCl_3_): *δ* = 187.6, 148.9, 140.5, 139.6, 130.5, 128.8, 128.8, 128.4, 128.4, 128.4, 128.4, 128.4, 128.4, 126.4, 105.9, 53.8, 39.1, 32.1, 22.4; HRMS (TOF ESI^+^): *m*/*z* calcd for C_19_H_21_ClNO^+^ [(M+H)^+^], 314.1306, found, 314.1303.

**(*Z*)-2-Chloro-3-((4-fluorophenyl)amino)-1-(4-methoxyphenyl)prop-2-en-1-one (2h).** V_Petroleum ether_/V_Ethyl acetate_ = 20:1, R*_f_* = 0.1; White solid: 142 mg (93%); mp = 130–132 °C; ^1^H NMR (600 MHz, DMSO-*d_6_*): *δ* = 9.53 (d, *J* = 12.9 Hz, 1H, NH), 7.80 (d, *J* = 12.9 Hz, 1H, C=CH), 7.61–7.59 (m, 2H, ArH), 7.23–7.21 (m, 2H, ArH), 7.15 (t, *J* = 8.7 Hz, 2H, ArH), 7.02–7.01 (m, 2H, ArH), 3.82 (s, 3H, ArOCH_3_); ^13^C NMR (150 MHz, DMSO-*d_6_*): *δ* = 186.2, 161.4, 158.5 (C-F, *J* = 238.5 Hz), 143.0, 137.2 (C-F, *J* = 1.5 Hz), 131.2, 130.6, 130.6, 118.9 (C-F, *J* = 9.0 Hz), 118.9 (C-F, *J* = 9.0 Hz), 116.1 (C-F, *J* = 22.7 Hz), 116.1 (C-F, *J* = 22.7 Hz), 113.8, 113.8, 107.3, 55.4; ^19^F NMR (500 MHz, DMSO-*d_6_*): *δ* = −119.3; HRMS (TOF ESI^+^): *m*/*z* calcd for C_16_H_13_ClFNNaO_2_^+^ [(M+Na)^+^], 328.0511, found, 328.0511.

**(*Z*)-2-Chloro-3-((4-chlorophenyl)amino)-1-(4-methoxyphenyl)prop-2-en-1-one (2i).** V_Petroleum ether_/V_Ethyl acetate_ = 10:1, R*_f_* = 0.2; Yellow solid: 140 mg (87%); mp = 178–179 °C; ^1^H NMR (600 MHz, CDCl_3_): *δ* = 7.79 (d, *J* = 13.1 Hz, 1H, C=CH), 7.64–7.62 (m, 2H, ArH), 7.28–7.27 (m, 2H, ArH), 7.14 (d, *J* = 13.0 Hz, 1H, NH), 6.97–6.95 (m, 2H, ArH), 6.89–6.86 (m, 2H, ArH), 3.88 (s, 3H, ArOCH_3_); ^13^C NMR (150 MHz, CDCl_3_): *δ* = 187.4, 162.3, 139.9, 138.1, 130.9, 130.9, 130.9, 130.0, 130.0, 129.0, 117.5, 117.5, 113.7, 113.9, 110.2, 55.5; HRMS (TOF ESI^+^): *m*/*z* calcd for C_16_H_14_Cl_2_NO_2_^+^ [(M+H)^+^], 322.0396, found, 322.0398.

**(*Z*)-3-(Benzylamino)-2-chloro-1-(4-methoxyphenyl)prop-2-en-1-one (2j)**. V_Petroleum ether_/V_Ethyl acetate_ = 15:1, R*_f_* = 0.1; Yellow solid: 114 mg (76%); mp = 170–172 °C; ^1^H NMR (600 MHz, DMSO-*d*_6_): *δ* = 7.84–7.80 (m, 1H, NH), 7.57 (d, *J* = 13.6 Hz, 1H, C=CH), 7.37–7.34 (m, 4H, ArH), 7.29–7.23 (m, 3H, ArH), 6.94–6.92 (m, 2H, ArH), 4.43 (d, *J* = 5.8 Hz, 2H, ArCH_2_), 3.79 (s, 3H, ArOCH_3_); ^13^C NMR (150 MHz, DMSO–*d*_6_): *δ* = 185.3, 160.9, 152.9, 139.2, 131.8, 130.2, 130.2, 128.7, 128.7, 127.4, 127.4, 113.6, 113.6, 95.6, 55.4, 55.4, 50.7; HRMS (TOF ESI^+^): *m*/*z* calcd for C_17_H_16_ClNNaO_2_^+^ [(M+Na)^+^], 324.0762, found, 324.0762.

**(*Z*)-2-Chloro-3-(cyclohexylamino)-1-(4-methoxyphenyl)prop-2-en-1-one (2k).** V_Petroleum ether_/V_Ethyl acetate_ = 10:1, R*_f_* = 0.1; Yellow solid: 112 mg (76%); mp = 170–172 °C; ^1^H NMR (600 MHz, CDCl_3_): *δ* = 7.51 (d, *J* = 8.3 Hz, 2H, ArH), 7.35 (d, *J* = 13.7 Hz, 1H, C=CH), 6.91 (d, *J* = 8.3 Hz, 2H, ArH), 5.32–5.29 (m, 1H, NH), 3.84 (s, 3H, ArOCH_3_), 3.14–3.09 (m, 1H, CH), 1.92–1.75 (m, 5H, CH_2_, CH_2_, CH_2_), 1.28 (t, *J* = 9.8 Hz, 4H, CH_2_, CH_2_), 1.20–1.15 (m, 1H, CH_2_); ^13^C NMR (150 MHz, CDCl_3_): *δ* = 186.8, 161.5, 147.7, 131.9, 130.4, 130.4, 113.6, 113.6, 105.6, 56.8, 55.5, 34.3, 34.3, 25.1, 25.1, 24.7; HRMS (TOF ESI^+^): *m*/*z* calcd for C_16_H_21_ClNO_2_^+^ [(M+H)^+^], 294.1255, found, 294.1232.

**(*Z*)-2-Chloro-1-(4-chlorophenyl)-3-(*p*-tolylamino)prop-2-en-1-one (2l).** V_Petroleum ether_/V_Ethyl acetate_ = 10:1, R*_f_* = 0.2; White solid: 131 mg (86%); mp = 169–171 °C; ^1^H NMR (500 MHz, CDCl_3_): *δ* = 7.78 (d, *J* = 13.3 Hz, 1H, C=CH), 7.58–7.55 (m, 2H, ArH), 7.45–7.42 (m, 2H, ArH), 7.20 (d, *J* = 13.4 Hz, 1H, NH), 7.13 (d, *J* = 8.1 Hz, 2H, ArH), 6.84 (d, *J* = 8.5 Hz, 2H, ArH), 2.31 (s, 3H, ArCH_3_); ^13^C NMR (125 MHz, CDCl_3_): *δ* = 186.8, 141.7, 137.3, 136.7, 136.7, 134.3, 130.6, 130.6, 130.1, 130.1, 128.8, 128.8, 116.6, 116.6, 109.0, 20.9; HRMS (TOF ESI^+^): *m*/*z* calcd for C_16_H_14_Cl_2_NO^+^ [(M+H)^+^], 306.0447, found, 306.0442.

**(*Z*)-2-Chloro-1-(4-chlorophenyl)-3-((4-methoxyphenyl)amino)prop-2-en-1-one (2m).** V_Petroleum ether_/V_Ethyl acetate_ = 10:1, R*_f_* = 0.1; Yellow solid: 107 mg (67%); mp = 160–162 °C; ^1^H NMR (600 MHz, CDCl_3_): *δ* = 7.71 (d, *J* = 13.3 Hz, 1H, C=CH), 7.55 (d, *J* = 8.0 Hz, 2H, ArH), 7.42 (d, *J* = 8.0 Hz, 2H, ArH), 7.21 (d, *J* = 13.4 Hz, 1H, NH), 6.90 (d, *J* = 8.7 Hz, 2H, ArH), 6.86 (d, *J* = 8.7 Hz, 2H, ArH), 3.78 (s, 3H, ArOCH_3_); ^13^C NMR (150 MHz, CDCl_3_): *δ* = 186.8, 156.8, 142.5, 137.3, 137.2, 132.5, 130.0, 130.0, 128.8, 128.8, 118.4, 118.4, 115.2, 115.2, 108.6, 55.7; HRMS (TOF ESI^+^): *m*/*z* calcd for C_16_H_14_Cl_2_NO_2_^+^ [(M+H)^+^], 322.0396, found, 322.0398.

**(*Z*)-2-Chloro-1-(4-chlorophenyl)-3-((4-chlorophenyl)amino)prop-2-en-1-one (2n).** V_Petroleum ether_/V_Ethyl acetate_ = 20:1, R*_f_* = 0.2; White solid: 149 mg (92%); mp = 191–192 °C; ^1^H NMR (600 MHz, CDCl_3_): *δ* = 7.78 (d, *J* = 13.2 Hz, 1H, C=CH), 7.58–7.54 (m, 2H, ArH), 7.45–7.44 (m, 2H, ArH), 7.30–7.29 (m, 2H, ArH), 7.26–7.25 (d, *J* = 15.1 Hz, 1H, NH), 6.89–6.87 (m, 2H, ArH); ^13^C NMR (150 MHz, CDCl_3_): *δ* = 187.2, 142.9, 137.7, 137.6, 137.0, 130.2, 130.2, 130.1, 130.1, 129.7, 129.0, 128.9, 117.9, 117.7, 102.1; HRMS (TOF ESI^+^): *m*/*z* calcd for C_15_H_11_Cl_3_NO^+^ [(M+H)^+^], 325.9901, found, 325.9878.

**(*Z*)-2-Chloro-1-(4-chlorophenyl)-3-(pyridin-2-ylamino)prop-2-en-1-one (2o).** V_Petroleum ether_/V_Ethyl acetate_ = 5:1, R*_f_* = 0.1; White solid: 100 mg (68%); mp = 196–198 °C; ^1^H NMR (500 MHz, CDCl_3_): *δ* = 8.53 (d, *J* = 12.4 Hz, 1H, C=CH), 8.24–8.22 (m, 1H, ArH), 7.66–7.62 (m, 1H, ArH), 7.62–7.59 (m, 2H, ArH), 7.54 (d, *J* = 12.5 Hz, 1H, NH), 7.47–7.44 (m, 2H, ArH), 6.99–6.96 (m, 1H, ArH), 6.82 (d, *J* = 8.2 Hz, 1H, ArH); ^13^C NMR (125 MHz, CDCl_3_): *δ* = 187.5, 150.8, 148.8, 139.3, 138.8, 137.6, 136.9, 130.3, 130.3, 128.9, 128.9, 119.1, 111.1, 110.9; HRMS (TOF ESI^+^): *m*/*z* calcd for C_14_H_11_Cl_2_N_2_O^+^ [(M+H)^+^], 293.0243, found, 293.0244.

**(*Z*)-2-Chloro-1-(2-chlorophenyl)-3-((4-methoxyphenyl)amino)prop-2-en-1-one (2p).** V_Petroleum ether_/V_Ethyl acetate_ = 5:1, R*_f_* = 0.2; Yellow solid: 93 mg (58%); mp = 152–154 °C; ^1^H NMR (600 MHz, CDCl_3_): *δ* = 7.51 (d, *J* = 13.5 Hz, 1H, NH), 7.44 (d, *J* = 8.0 Hz, 1H, ArH), 7.40–7.37 (m, 1H, C=CH), 7.35–7.33 (m, 2H, ArH), 6.87–6.83 (m, 5H, ArH), 3.77 (s, 3H, ArOCH_3_); ^13^C NMR (150 MHz, CDCl_3_): *δ* = 157.1, 132.4, 131.2, 130.9, 130.8, 130.2, 129.0, 126.9, 126.9, 118.9, 118.9, 118.7, 118.7, 115.2, 115.2, 55.7; HRMS (TOF ESI^+^): *m*/*z* calcd for C_16_H_13_Cl_2_NNaO_2_^+^ [(M+Na)^+^], 344.0216, found, 344.0224.

**(*Z*)-1-(4-Bromophenyl)-2-chloro-3-(*p*-tolylamino)prop-2-en-1-one (2q).** V_Petroleum ether_/V_Ethyl acetate_ = 8:1, R*_f_* = 0.2; Yellow solid: 139 mg (80%); mp = 168–170 °C; ^1^H NMR (600 MHz, CDCl_3_): *δ* = 7.82–7.77 (m, 1H, C=CH), 7.60 (d, *J* = 8.0 Hz, 2H, ArH), 7.50–7.47 (m, 2H, ArH), 7.25–7.17 (m, 1H, NH), 7.13 (d, *J* = 8.4 Hz, 2H), 6.84–6.83 (m, 2H, ArH), 2.31 (s, 3H, ArCH_3_); ^13^C NMR (150 MHz, CDCl_3_): *δ* = 187.1, 144.0, 141.7, 137.8, 136.6, 134.5, 131.8, 131.8, 130.6, 130.6, 130.3, 125.6, 116.8, 116.6, 101.1, 20.9; HRMS (TOF ESI^+^): *m*/*z* calcd for C_16_H_14_BrClNO^+^ [(M+H)^+^], 349.9942, found, 349.9941.

**(*Z*)-(2-Chloro-3-((4-methoxyphenyl)amino)-1-(4-nitrophenyl)allylidene)oxonium (2r).** V_Petroleum ether_/V_Ethyl acetate_ = 10:1, R*_f_* = 0.2; Brown solid: 116 mg (70%); mp = 189–191 °C; ^1^H NMR (600 MHz, CDCl_3_): *δ* = 8.33–8.30 (m, 2H, ArH), 7.77–7.75 (m, 2H, ArH), 7.68 (d, *J* = 13.4 Hz, 1H, C=CH), 7.26 (d, *J* = 13.1 Hz, 1H, NH), 6.91–6.89 (m, 2H, ArH), 6.88–6.86 (m, 2H, ArH), 3.78 (s, 3H, ArOCH_3_); ^13^C NMR (150 MHz, CDCl_3_): *δ* = 185.8, 157.2, 149.1, 144.9, 142.9, 132.1, 129.4, 129.4, 123.8, 123.8, 123.8, 118.9, 118.7, 115.3, 108.3, 55.7; HRMS (TOF ESI^+^): *m*/*z* calcd for C_16_H_13_ClN_2_NaO_4_^+^ [(M+Na)^+^], 355.0456, found, 355.0459.

**(*Z*)-2-Chloro-3-((4-fluorophenyl)amino)-1-(4-nitrophenyl)prop-2-en-1-one (2s).** V_Petroleum ether_/V_Ethyl acetate_ = 5:1, R*_f_* = 0.2; Yellow solid: 117 mg (73%); mp = 218–220 °C; ^1^H NMR (500 MHz, DMSO-*d*_6_): *δ* = 9.82 (s, 1H, NH), 8.29 (d, *J* = 8.2 Hz, 2H, ArH), 7.84 (d, *J* = 8.3 Hz, 2H, ArH), 7.79 (s, 1H, C=CH), 7.29–7.26 (m, 2H, ArH), 7.16–7.13 (m, 2H, ArH); ^13^C NMR (125 MHz, DMSO-*d*_6_): *δ* = 185.3, 159.8 (C-F, *J* = 238.8 Hz), 148.5, 145.1, 145.1, 136.7 (C-F, *J* = 2.5 Hz), 129.7, 129.7, 123.7, 123.7, 119.6 (C-F, *J* = 7.5 Hz), 119.6 (C-F, *J* = 7.5 Hz), 116.1 (C-F, *J* = 22.8 Hz), 116.1 (C-F, *J* = 22.8 Hz), 106.9; ^19^F NMR (500 MHz, DMSO-*d_6_*): *δ* = −120.1; HRMS (TOF ESI^+^): *m*/*z* calcd for C_15_H_11_ClFN_2_O_3_^+^ [(M+H)^+^], 321.0437, found, 321.0442.

**(*Z*)-1-([1,1’-Biphenyl]-4-yl)-2-chloro-3-(phenylamino)prop-2-en-1-one (2t).** V_Petroleum ether_/V_Ethyl acetate_ = 5:1, R*_f_* = 0.1; White solid: 61 mg (37%); mp = 152–153 °C; ^1^H NMR (600 MHz, CDCl_3_): *δ* = 7.93 (d, *J* = 13.3 Hz, 1H, C=CH), 7.73–7.69 (m, 4H, ArH), 7.65 (d, *J* = 7.0 Hz, 2H, ArH), 7.50–7.48 (m, 2H, ArH), 7.44–7.40 (m, 1H, ArH), 7.34–7.32 (m, 2H, ArH), 7.19 (d, *J* = 13.2 Hz, 1H, NH), 7.11–7.08 (m, 1H, ArH), 6.95 (d, *J* = 8.0 Hz, 2H, ArH); ^13^C NMR (150 MHz, CDCl_3_): *δ* = 187.9, 144.1, 141.1, 140.1, 139.2, 137.5, 130.1, 130.1, 129.3, 129.3, 129.1, 129.1, 128.2, 127.4, 127.3, 127.3, 127.3, 124.3, 116.4, 116.4, 109.9; HRMS (TOF ESI^+^): *m*/*z* calcd for C_21_H_17_ClNO^+^ [(M+H)^+^], 334.0993, found, 334.0993.

**(*Z*)-2-Chloro-1-(naphthalen-2-yl)-3-(naphthalen-2-ylamino)prop-2-en-1-one (2u).** V_Petroleum ether_/V_Ethyl acetate_ = 20:1, R*_f_* = 0.3; White solid: 164 mg (92%); mp = 174–175 °C; ^1^H NMR (600 MHz, CDCl_3_): *δ* = 8.15 (s, 1H, ArH), 8.10 (d, *J* = 13.2 Hz, 1H, ArH), 7.97–7.93 (m, 3H, ArH), 7.78–7.75 (m, 3H, ArH), 7.66 (d, *J* = 8.3 Hz, 1H, C=CH), 7.63–7.57 (m, 2H, ArH), 7.48–7.43 (m, 2H, ArH), 7.40–7.37 (m, 1H, ArH), 7.26 (s, 1H, ArH), 7.10–7.08 (m, 1H, NH); ^13^C NMR (150 MHz, CDCl_3_): *δ* = 188.4, 143.4, 136.7, 136.2, 134.6, 134.0, 132.6, 130.6, 130.4, 129.1, 129.0, 128.6, 128.0, 128.0, 127.9, 127.4, 127.1, 127.1, 125.7, 125.3, 117.1, 112.5, 102.6; HRMS (TOF ESI^+^): *m*/*z* calcd for C_23_H_16_ClNNaO^+^ [(M+Na)^+^], 380.0813, found, 380.0821.

**(*Z*)-2-Chloro-3-((4-methoxyphenyl)amino)-1-(pyridin-4-yl)prop-2-en-1-one (2v).** V_Petroleum ether_/V_Ethyl acetate_ = 15:1, R*_f_* = 0.2; Yellow solid: 122 mg (85%); mp = 145–147 °C; ^1^H NMR (500 MHz, CDCl_3_): *δ* = 8.74–8.73 (m, 2H, ArH), 7.67 (d, *J* = 13.5 Hz, 1H, C=CH), 7.45–7.43 (m, 2H, ArH), 7.36 (d, *J* = 13.4 Hz, 1H, NH), 6.91–6.85 (m, 4H, ArH), 3.78 (s, 3H, ArOCH_3_); ^13^C NMR (125 MHz, CDCl_3_): *δ* = 185.7, 157.2, 150.3, 150.3, 146.4, 143.2, 132.3, 122.3, 122.3, 118.7, 118.7, 115.3, 115.3, 108.2, 55.7; HRMS (TOF ESI^+^): *m*/*z* calcd for C_15_H_14_ClN_2_O_2_^+^ [(M+H)^+^], 289.0738, found, 289.0720.

**(*Z*)-2-Chloro-3-((4-chlorophenyl)amino)-1-(pyridin-4-yl)prop-2-en-1-one (2w).** V_Petroleum ether_/V_Ethyl acetate_ = 15:1, R*_f_* = 0.1; Yellow solid: 124 mg (85%); mp = 152–153 °C; ^1^H NMR (600 MHz, CDCl_3_): *δ* = 8.77–8.76 (m, 2H, ArH), 7.74 (d, *J* = 13.2 Hz, 1H, C=CH), 7.45–7.44 (m, 2H, ArH), 7.36–7.33 (m, 1H, NH), 7.32–7.29 (m, 2H, ArH), 6.89–6.86 (m, 2H, ArH); ^13^C NMR (150 MHz, CDCl_3_): *δ* = 186.2, 150.5, 150.5, 146.1, 143.7, 137.4, 130.3, 130.2, 130.2, 122.3, 122.3, 118.1, 118.1, 101.5; HRMS (TOF ESI^+^): *m*/*z* calcd for C_14_H_10_Cl_2_N_2_NaO^+^ [(M+Na)^+^], 315.0062, found, 315.0062.

**(*Z*)-2-Chloro-3-(mesitylamino)cyclohex-2-en-1-one (2x).** V_Petroleum ether_/V_Ethyl acetate_ = 1:1, R*_f_* = 0.2; White solid: 67 mg (51%); mp = 160–162 °C ^1^H NMR (600 MHz, CDCl_3_): *δ* = 6.93 (s, 2H, ArH), 6.68 (s, 1H, NH), 2.50 (t, *J* = 6.4 Hz, 2H, CH_2_), 2.29 (s, 3H, ArCH_3_), 2.17–2.14 (m, 8H, ArCH_3_, ArCH_3_, and CH_2_), 1.90–1.86 (m, 2H, CH_2_); ^13^C NMR (150 MHz, CDCl_3_): *δ* = 188.5, 159.9, 138.4, 136.5, 132.2, 129.4, 129.4, 129.4, 104.5, 37.2, 26.5, 21.1, 21.1, 18.3, 18.3; HRMS (TOF ESI^+^): *m*/*z* calcd for C_15_H_19_ClNO^+^ [(M+H)^+^], 264.1150, found, 264.1159.

**(*Z*)-3-(Benzylamino)-2-chlorocyclohex-2-en-1-one (2y).** V_Petroleum ether_/V_Ethyl acetate_ = 1:1, R*_f_* = 0.2; White solid: 60 mg (51%); mp = 153–154 °C; ^1^H NMR (600 MHz, CDCl_3_): *δ* = 7.41–7.38 (m, 2H, ArH), 7.35–7.32 (m, 1H, ArH), 7.26 (d, *J* = 5.7 Hz, 2H, ArH), 5.98–5.96 (m, 1H, NH), 4.51 (d, *J* = 6.2 Hz, 2H, ArCH_2_), 2.58 (t, *J* = 6.2 Hz, 2H, CH_2_), 2.48 (t, *J* = 6.5 Hz, 2H, CH_2_), 1.99–1.95 (m, 2H, CH_2_); ^13^C NMR (150 MHz, CDCl_3_): *δ* = 188.0, 159.7, 137.1, 129.3, 128.2, 126.9, 126.9, 126.9, 104.1, 47.2, 36.7, 26.3, 20.8; HRMS (TOF ESI^+^): *m*/*z* calcd for C_13_H_15_ClNO^+^ [(M+H)^+^], 236.0837, found, 236.0841.

**(*Z*)-2-Chloro-3-(mesitylamino)-5,5-dimethylcyclohex-2-en-1-one (2z).** V_Petroleum ether_/V_Ethyl acetate_ = 5:1, R*_f_* = 0.2; White solid: 73 mg (50%); mp = 152–153 °C; ^1^H NMR (600 MHz, CDCl_3_): *δ* = 6.96 (s, 2H, ArH), 6.76 (s, 1H, NH), 2.41 (s, 2H, CH_2_), 2.32 (s, 3H, ArCH_3_), 2.17 (s, 6H, ArCH_3_, ArCH_3_), 2.01 (s, 2H, CH_2_), 1.01 (s, 6H, CH_3_); ^13^C NMR (150 MHz, CDCl_3_): *δ* = 188.0, 159.8, 138.5, 136.6, 136.6, 132.4, 129.5, 129.5, 95.5, 50.8, 40.4, 32.4, 28.3, 28.3, 21.2, 18.4, 18.4; HRMS (TOF ESI^+^): *m*/*z* calcd for C_17_H_23_ClNO^+^ [(M+H)^+^], 292.1463, found, 292.1468.

**(*Z*)-2-Chloro-3-((2,6-diisopropylphenyl)amino)-5,5-dimethylcyclohex-2-en-1-one (2a’).** V_Petroleum ether_/V_Ethyl acetate_ = 10:1, R*_f_* = 0.2; White solid: 113 mg (68%); mp = 166–167 °C; ^1^H NMR (500 MHz, CDCl_3_): *δ* = 7.39–7.36 (m, 1H, ArH), 7.22 (d, *J* = 7.8 Hz, 2H, ArH), 6.78 (s, 1H, NH), 3.03–2.98 (m, 2H, CH, CH), 2.41 (s, 2H, CH_2_), 2.02 (s, 2H, CH_2_), 1.25 (d, *J* = 7.0 Hz, 6H, CH_3_, CH_3_), 1.17 (d, *J* = 6.8 Hz, 6H, CH_3_, CH_3_), 1.01 (s, 6H, CH_3_, CH_3_); ^13^C NMR (125 MHz, CDCl_3_): *δ* = 188.0, 159.6, 147.2, 147.2, 132.0, 129.5, 124.1, 124.1, 95.7, 51.0, 40.7, 32.4, 28.6, 28.6, 28.2, 28.2, 24.8, 24.8, 22.7, 22.7; HRMS (TOF ESI^+^): *m*/*z* calcd for C_20_H_29_ClNO^+^ [(M+H)^+^], 334.1932, found, 334.1933.

**(*Z*)-4-Chloro-3-methyl-1-phenyl-1*H*-pyrazol-5-amine (2b’).** V_Petroleum ether_/V_Ethyl acetate_ = 8:1, R*_f_* = 0.2; White solid: 96 mg (92%); mp = 114–116 °C; ^1^H NMR (600 MHz, CDCl_3_): *δ* = 7.53 (d, *J* = 7.9 Hz, 2H, ArH), 7.49–7.45 (m, 2H, ArH), 7.36–7.32 (m, 1H, ArH), 3.87 (s, 2H, NH_2_), 2.24 (s, 3H, CH_3_); ^13^C NMR (150 MHz, CDCl_3_): *δ* = 146.1, 141.1, 138.7, 129.7, 129.7, 127.6, 123.4, 123.4, 93.2, 11.9; HRMS (TOF ESI^+^): *m*/*z* calcd for C_10_H_11_ClN_3_^+^ [(M+H)^+^], 208.0636, found, 208.0641.

## 4. Conclusions

In conclusion, a metal-free C(sp^2^)-H chlorination reaction of enaminones has been accomplished, in which DCE has been excavated both as a “Cl” source and as a solvent. Highly functionalized *α*-chlorinated enaminones with a *Z*-configuration have been synthesized in 37–96% yields via a NBS-mediated vinyl C(sp^2^)-H bond chlorination process. This mild approach not only advances the vinyl C(sp^2^)-H bond chlorination, employing DCE as a “Cl” source, but also provides a new strategy for accessing chlorinated enaminone derivatives.

## Figures and Tables

**Table 1:** Optimization of reaction conditions *^a^*.

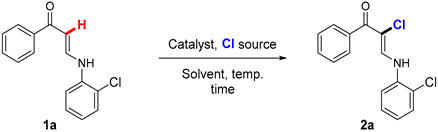					
Entry	Promoter (eq.)	“Cl” Source + Solvent	Temp. (°C)	Time (h)	Yield *^b^* (%)
1	TBAI (1.0)	DCE	140	1	n.d. *^c^*
2	TBAC (1.0)	DCE	140	1	n.d. *^c^*
3	NIS (1.0)	DCE	140	1	Trace
4	HBr (1.0)	DCE	140	1	n.d. *^c^*
5	PIDA (1.0)	DCE	140	1	n.d. *^c^*
6	NBS (1.0)	DCE	140	1	58
7	NBS (1.0)	DCE + MeCN	140	1	25
8	NBS (1.0)	DCE + THF	140	1	18
9	NBS (1.0)	DCE + 1,4-dioxane	140	1	20
10	NBS (1.0)	DCE + EA	140	1	15
11	NBS (1.0)	DCE + MeOH	140	1	27
12	NBS (1.0)	DCE + DMSO	140	1	11
13	NBS (1.0)	DCE + Toluene	140	1	32
14	NBS (1.0)	DCM + Toluene	140	1	trace
15	NBS (1.0)	CHCl_3_ + Toluene	140	1	trace
16	NBS (1.0)	CCl_4_ + Toluene	140	1	15
17	NBS (1.0)	DCE	140	3	65
18	NBS (1.0)	DCE	140	5	72
19	NBS (1.0)	DCE	140	7	84
**20**	**NBS** **(1.0)**	**DCE**	**140**	**9**	**90**
21	NBS (1.0)	DCE	140	11	88
22	NBS (0.5)	DCE	140	9	43
23	NBS (1.5)	DCE	140	9	38
24	NBS (1.0)	DCE	160	9	89
25	NBS (1.0)	DCE	120	9	72

*^a^* Reaction conditions: **1a** (0.1 mmol), catalyst, “Cl” source (0.5 mL) in 0.5 mL of solvent. ^*b*^ Isolated yields. *^c^* Not detected.

**Table 2:** Substrate scope of enaminones *^a,b^*.

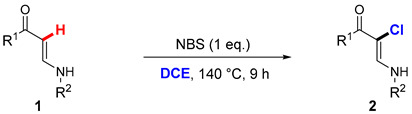
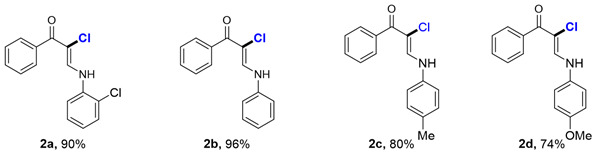
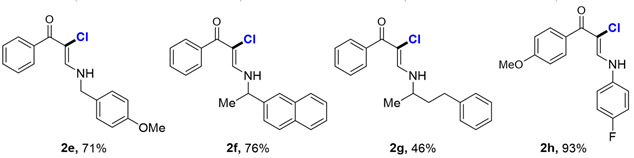
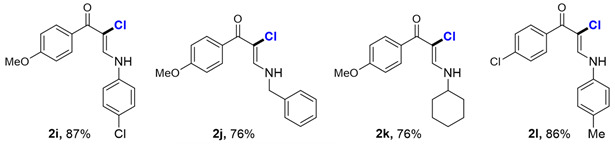
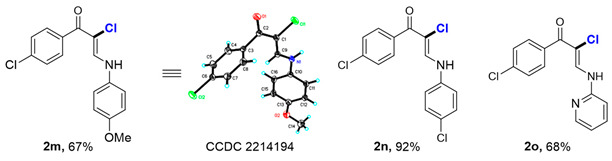
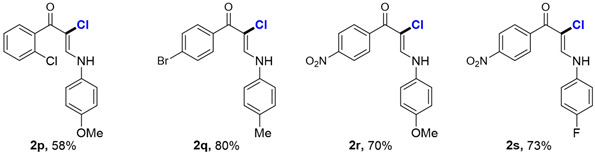
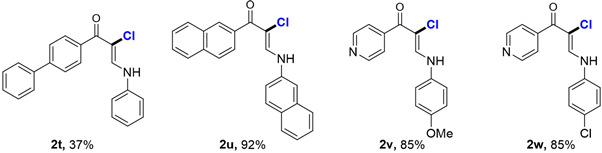
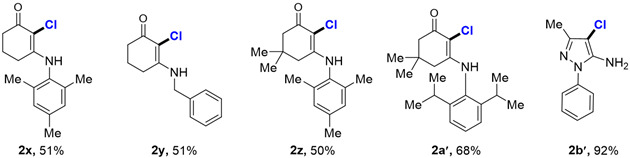

*^a^* Reaction conditions: **1a** (0.5 mmol), catalyst in 2 mL of solvent stirred at 140 °C for 9 h. ^*b*^ Isolated yields.

## Data Availability

Data are contained within the article.

## Supplementary Materials

The following supporting information can be downloaded at: https://www.mdpi.com/article/10.3390/ijms252212073/s1.
