# Supplementary material for: NBS-Mediated C(sp2)-H Bond Chlorination of Enaminones: Using DCE as Chlorine Source
Source: Int J Mol Sci. 2024 Nov 10;25(22):12073. doi: 10.3390/ijms252212073 (PMC11593413; doi:10.3390/ijms252212073)
Supplement: Supplementary file 1 [file ijms-25-12073-s001.zip › ijms-3276580-supplementary.pdf]

NBS-Mediated C(sp^2)-H Bond Chlorination of Enaminones: Using DCE as Chlorine Source

Menglin Peng[1,†], Yunhua Xie[1,†], Siyu Song[1], Zhilai Zhang[1], Yuanzheng Wei[1], Huimin Hu[1], Yongchao Wang[2,*], Fuchao Yu[1,*]

[1] Faculty of Life Science and Technology, Kunming University of Science and Technology, Kunming, 650500, P. R. China.
E-mail: yufuchao05@126.com; yufc@kust.edu.cn

[2] Faculty of Chemistry and Chemical Engineering, Yunnan Normal University, Kunming 650500, P. R. China.
Email: ycwang@ynnu.edu.cn

[†] These authors contributed equally to this study

Supporting Information

Table of Contents

1. X-ray Structure and Data[1] of **2m** (CCDC 2214194). 2
2. ^{1}H NMR and ^{13}C NMR spectra for spectroscopic data. 3
4. References and notes. 90

1. X-ray Structure and Data[1] of 2m (CCDC 2214194).

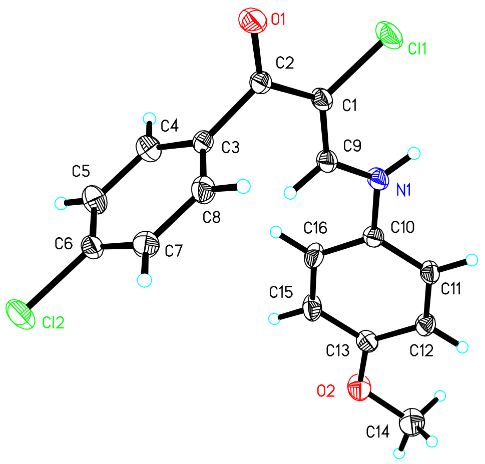

Figure S1 X-Ray crystal structure of **2m.**

Table S1 Crystal data and structure refinement for **2m.**

Empirical formula	$C_{16}H_{13}Cl_2NO_2$
Formula weight	322.17
Temperature	296.00 K
Crystal system, space group	Orthorhombic, Pbca
Unit cell dimensions	a = 13.0984(15) A alpha = 90 deg.
	b = 8.2937(10) A beta = 90 deg.
	c = 27.577(3) A gamma = 90 deg.
Volume	2995.8(6) A^3
Z, Calculated density	8, 1.429 Mg/m^3
Absorption coefficient	0.436 mm^-1
F(000)	1328.0
Theta range for data collection	2.145 to 27.656 deg.
Limiting indices	-13<=h<=17, -10<=k<=10, -33<=l<=35
Reflections collected / unique	16838 / 3458 [R(int) = 0.0372]
Data/restraints/parameters	3458 / 0 / 192
Goodness-of-fit on F^2	1.032
Final R indices [I>2sigma(I)]	R1 = 0.0406, wR2 = 0.0940
R indices (all data)	R1 = 0.0634, wR2 = 0.1054
Largest diff. peak and hole	0.237 and -0.292 e.A^-3

2. ^{1}H NMR and ^{13}C NMR spectra for spectroscopic data.

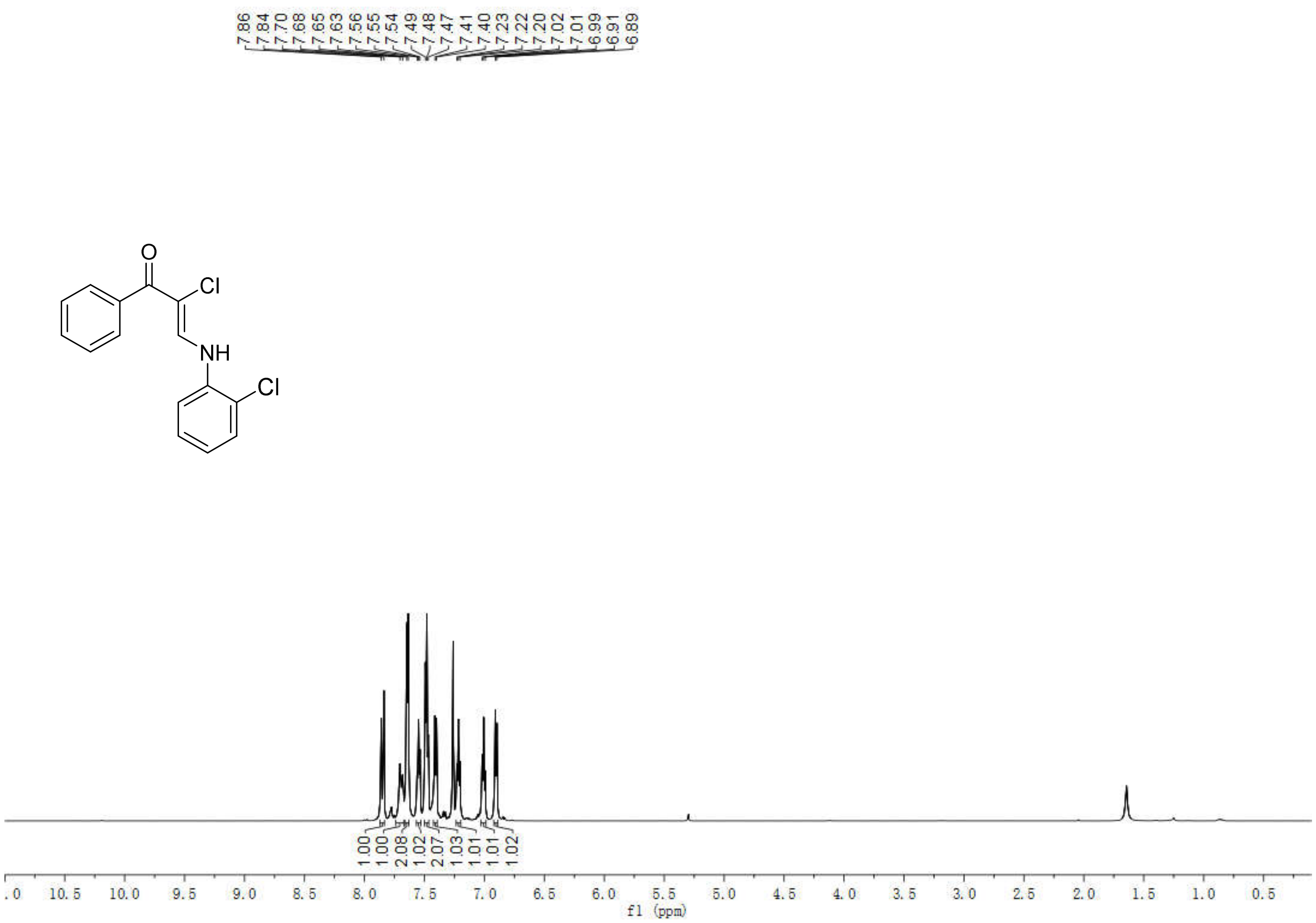

Figure S2. ^{1}H NMR (600 MHz, $CDCl_3$) spectra of compound **2a**

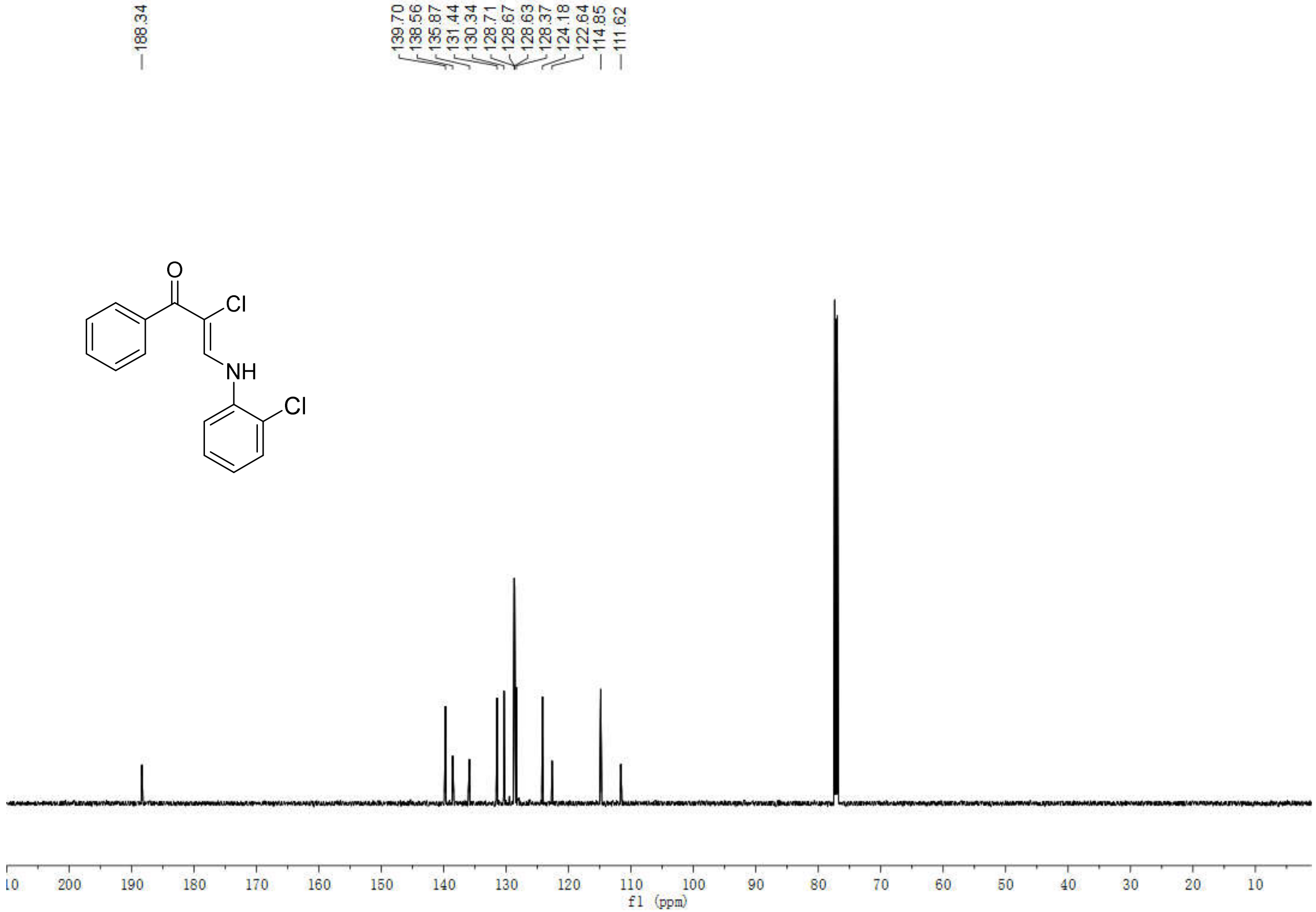

Figure S3. ^{13}C NMR (600 MHz, $CDCl_3$) spectra of compound **2a**

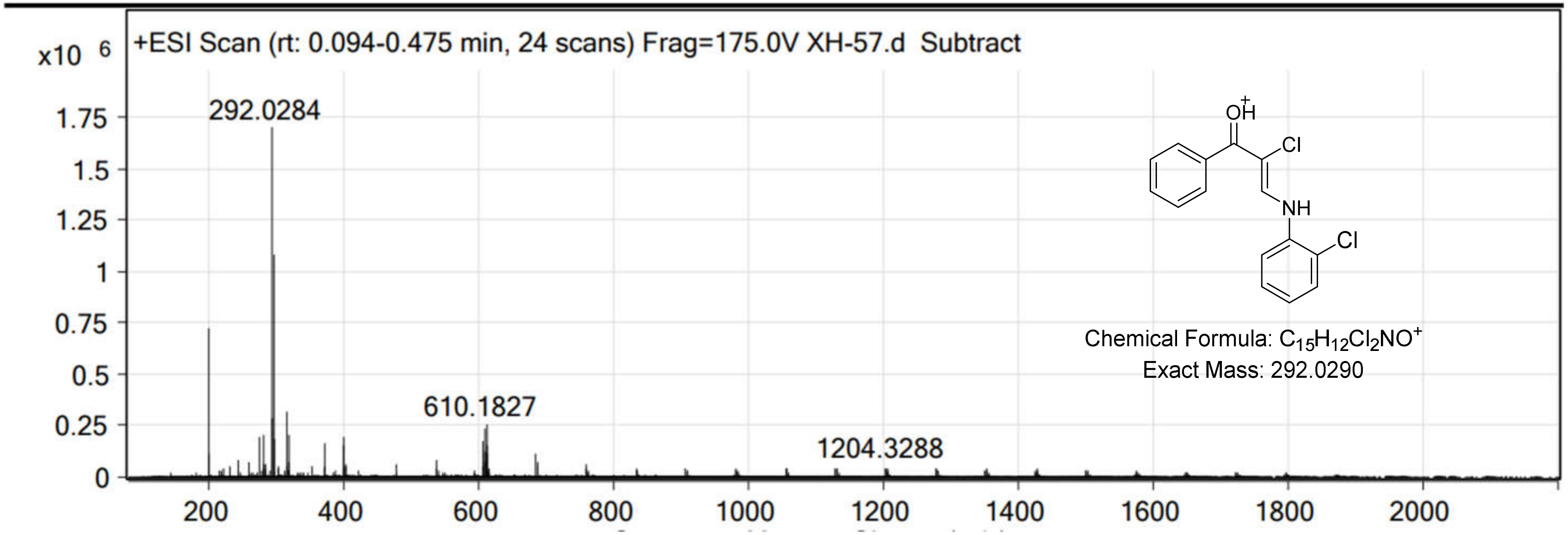

Figure S4. HRMS spectra of compound **2a**

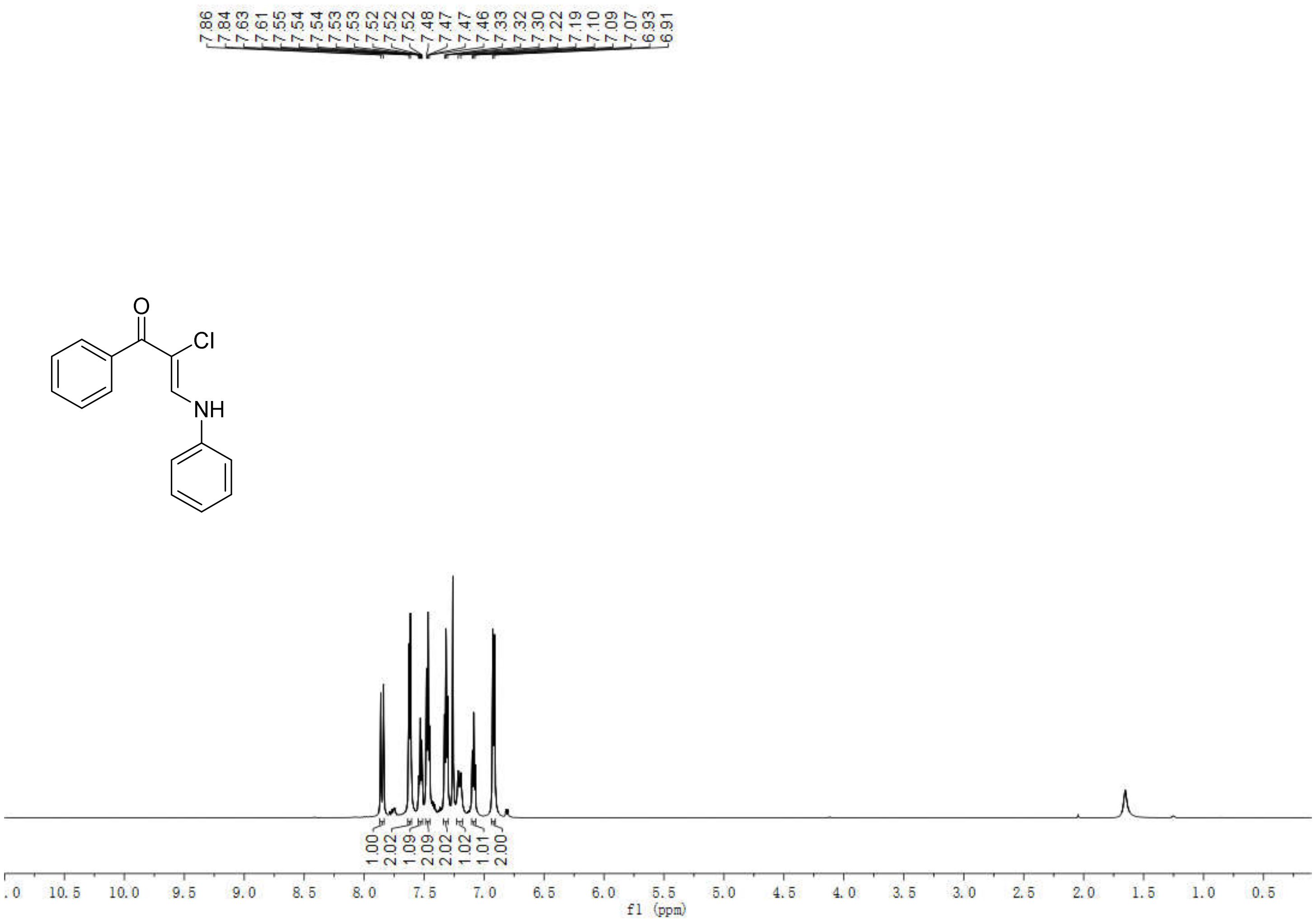

Figure S5. ^{1}H NMR (600 MHz, $CDCl_3$) spectra of compound **2b**

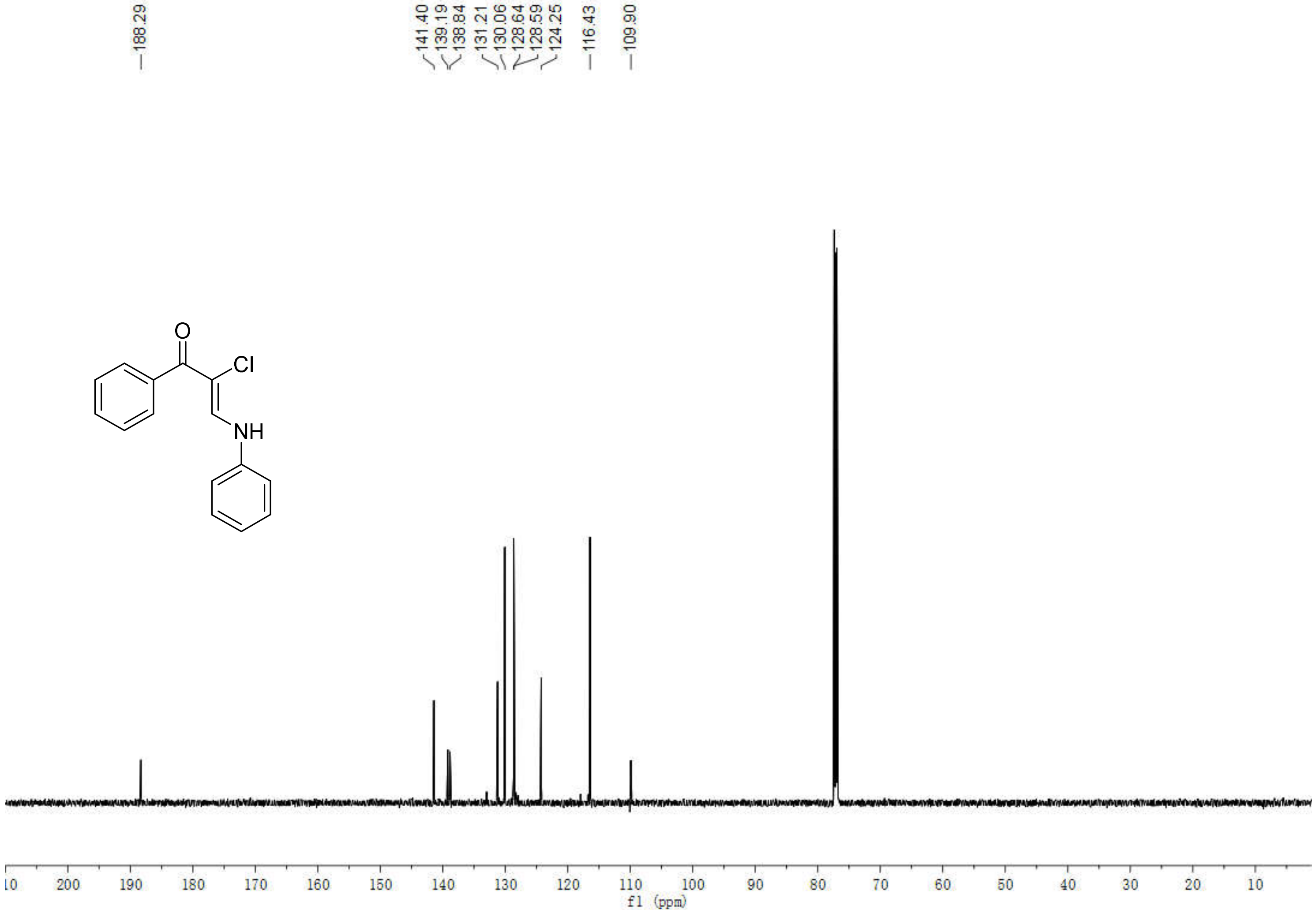

Figure S6. ^{13}C NMR (600 MHz, $CDCl_3$) spectra of compound **2b**

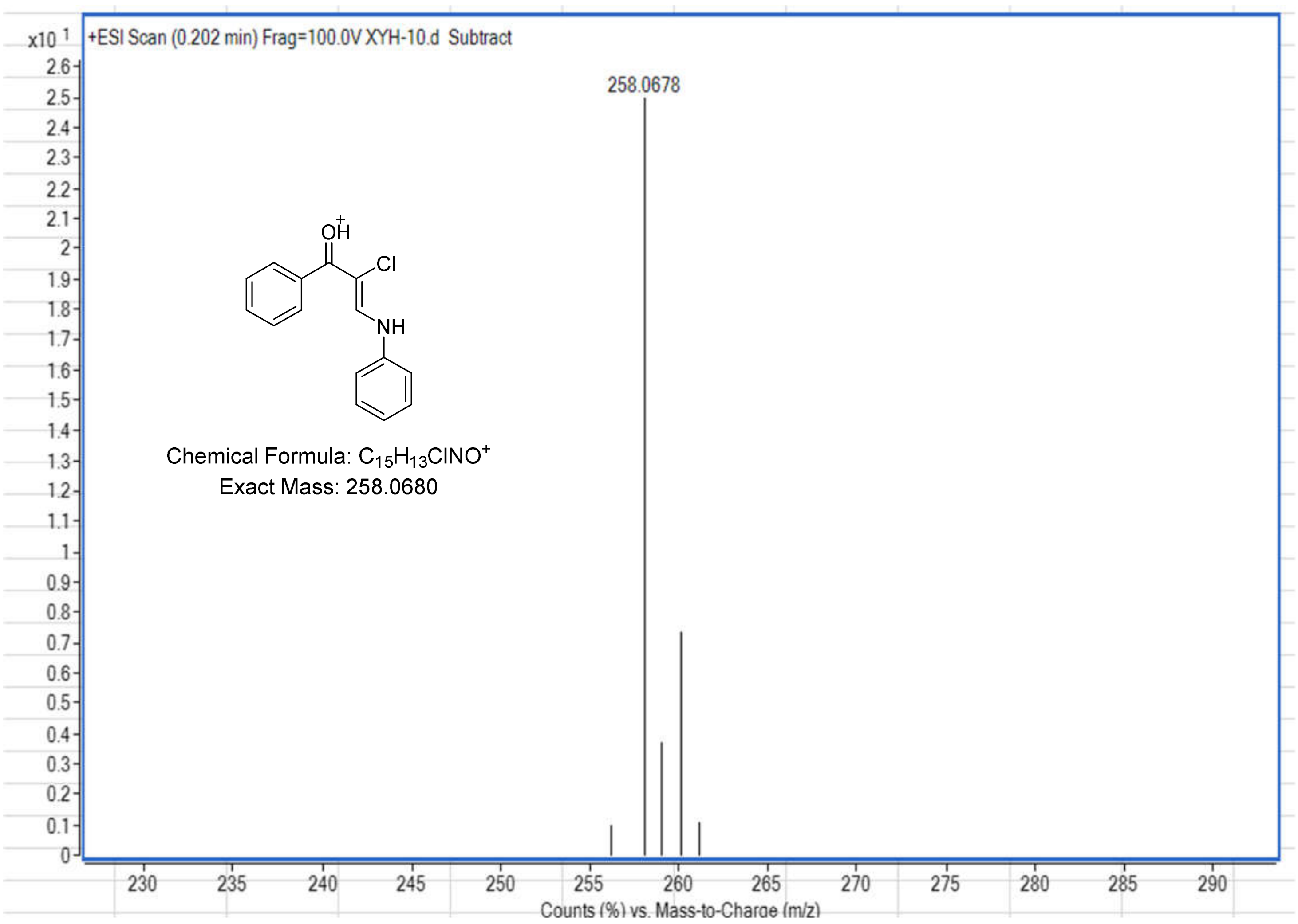

Figure S7. HRMS spectra of compound **2b**

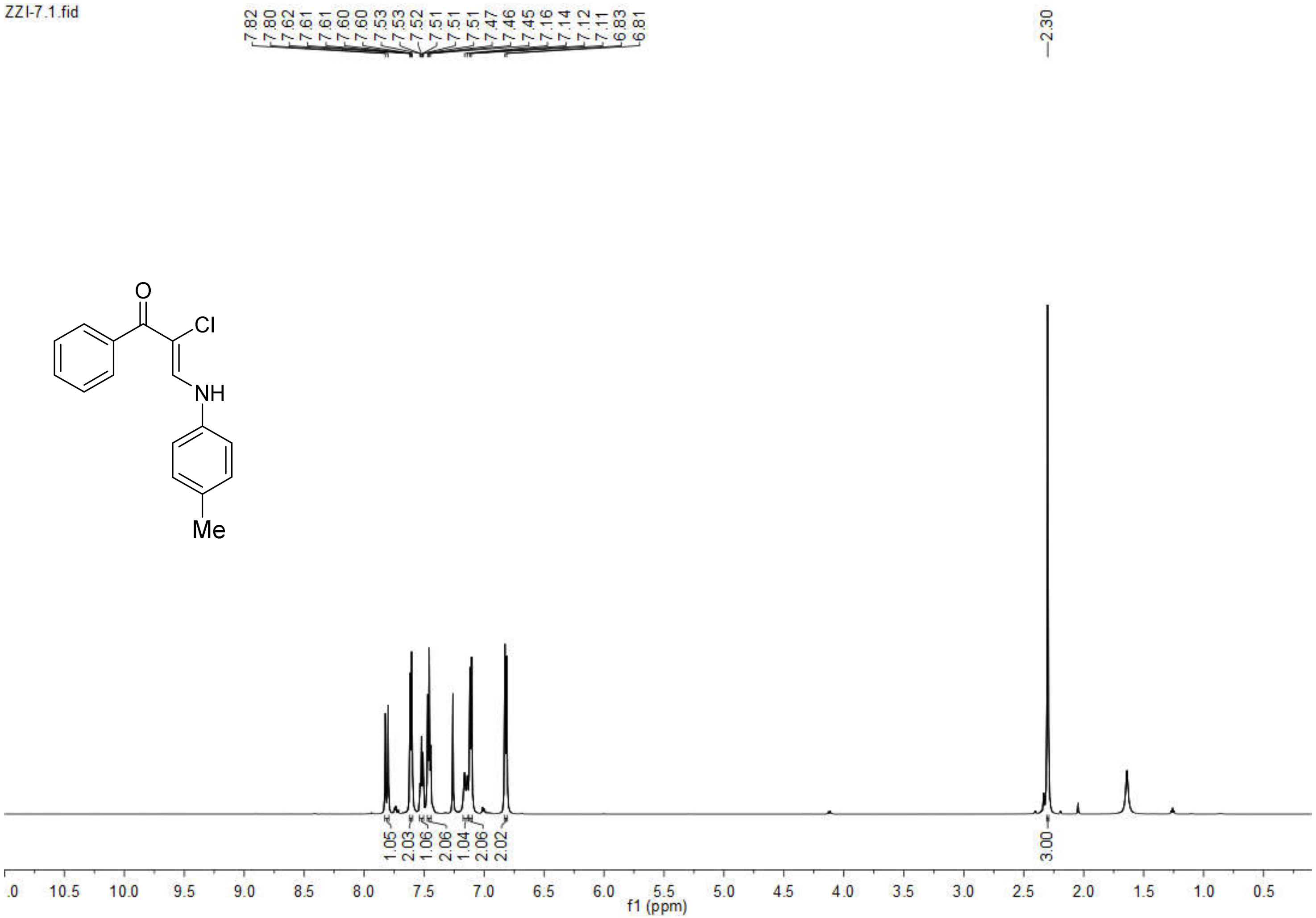

Figure S8. ^{1}H NMR (600 MHz, $CDCl_3$) spectra of compound **2c**

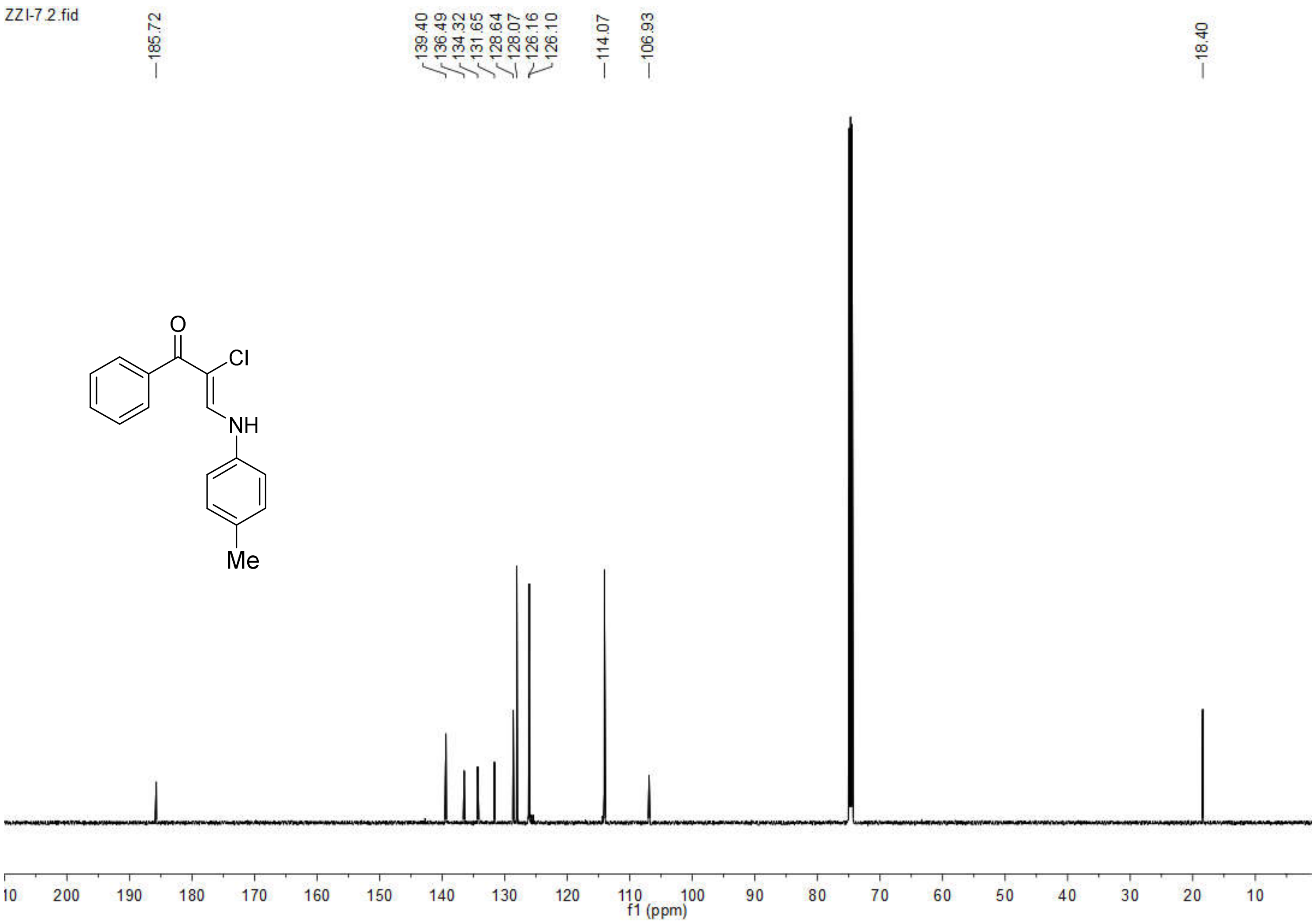

Figure S9. ^{13}C NMR (600 MHz, $CDCl_3$) spectra of compound **2c**

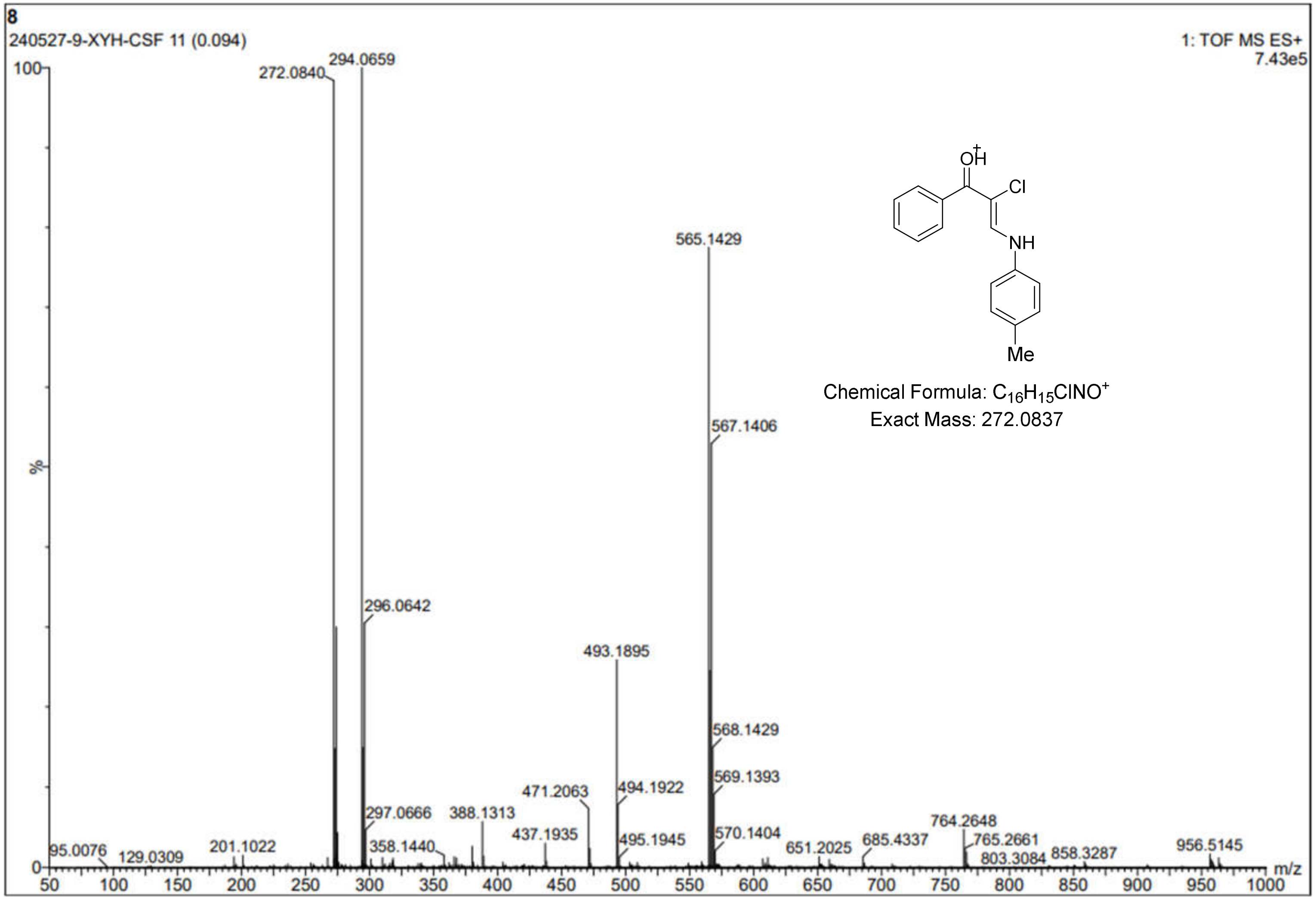

Figure S10. HRMS spectra of compound **2c**

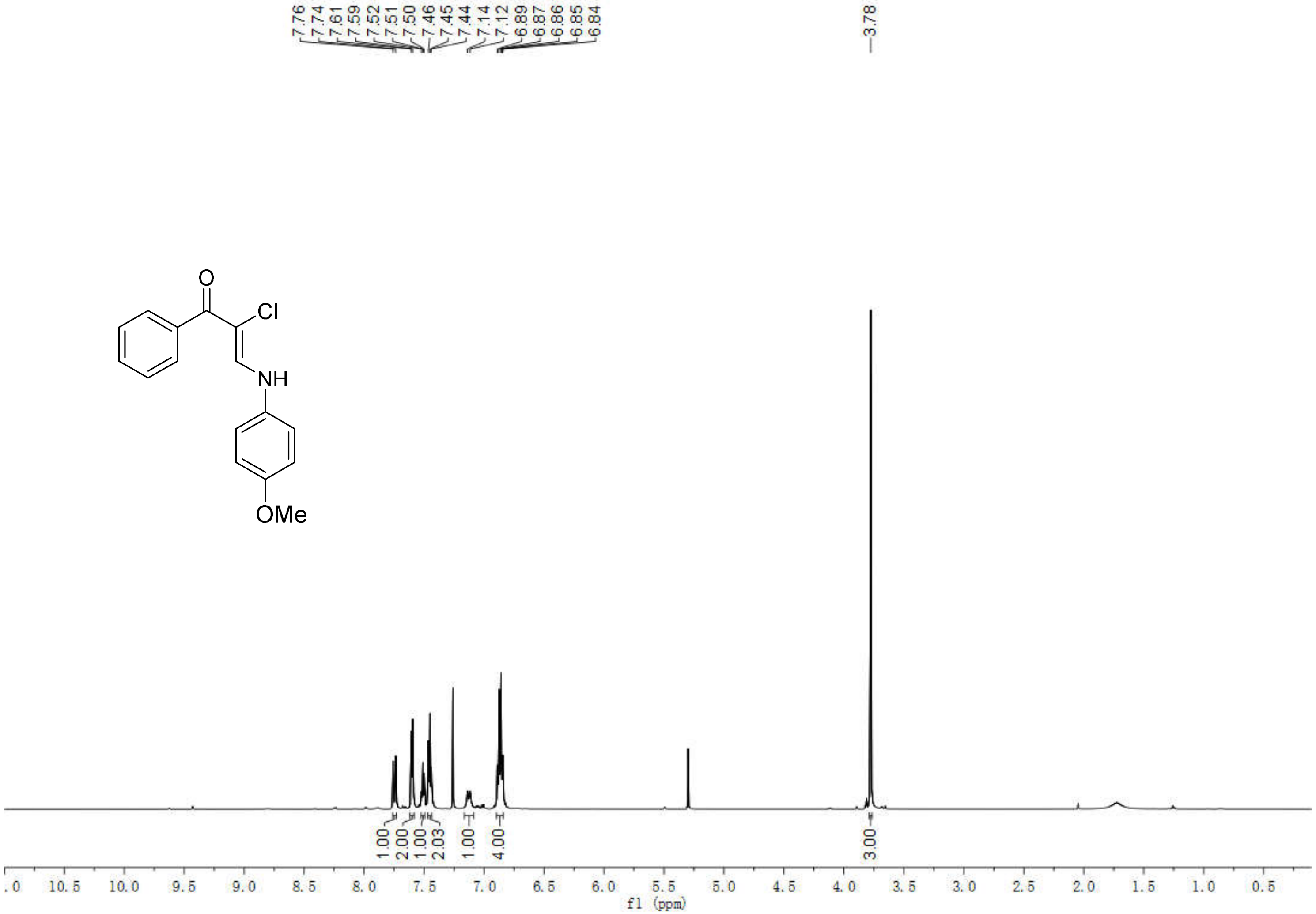

Figure S11. ^{1}H NMR (600 MHz, $CDCl_3$) spectra of compound **2d**

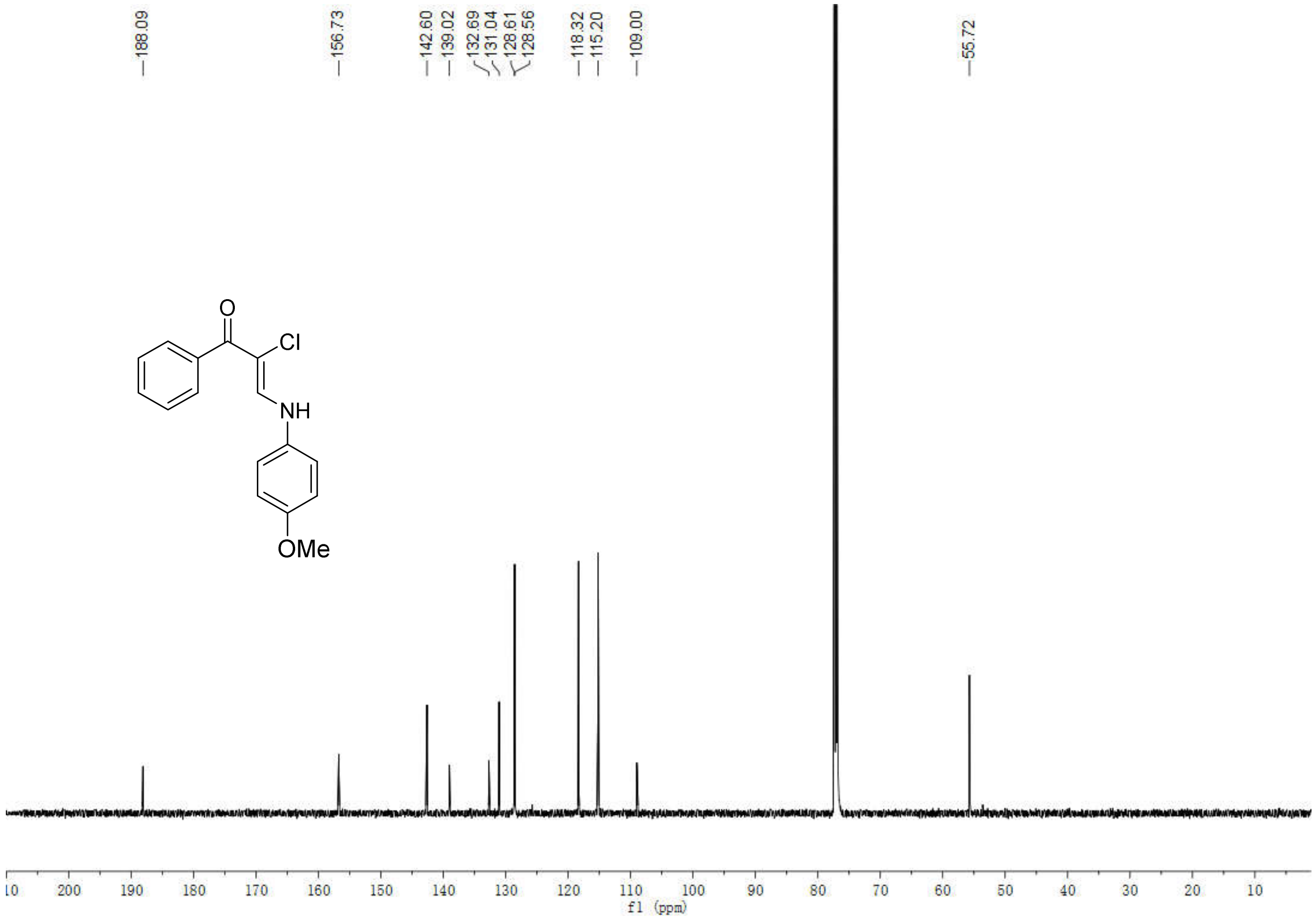

Figure S12. ^{13}C NMR (600 MHz, $CDCl_3$) spectra of compound **2d**

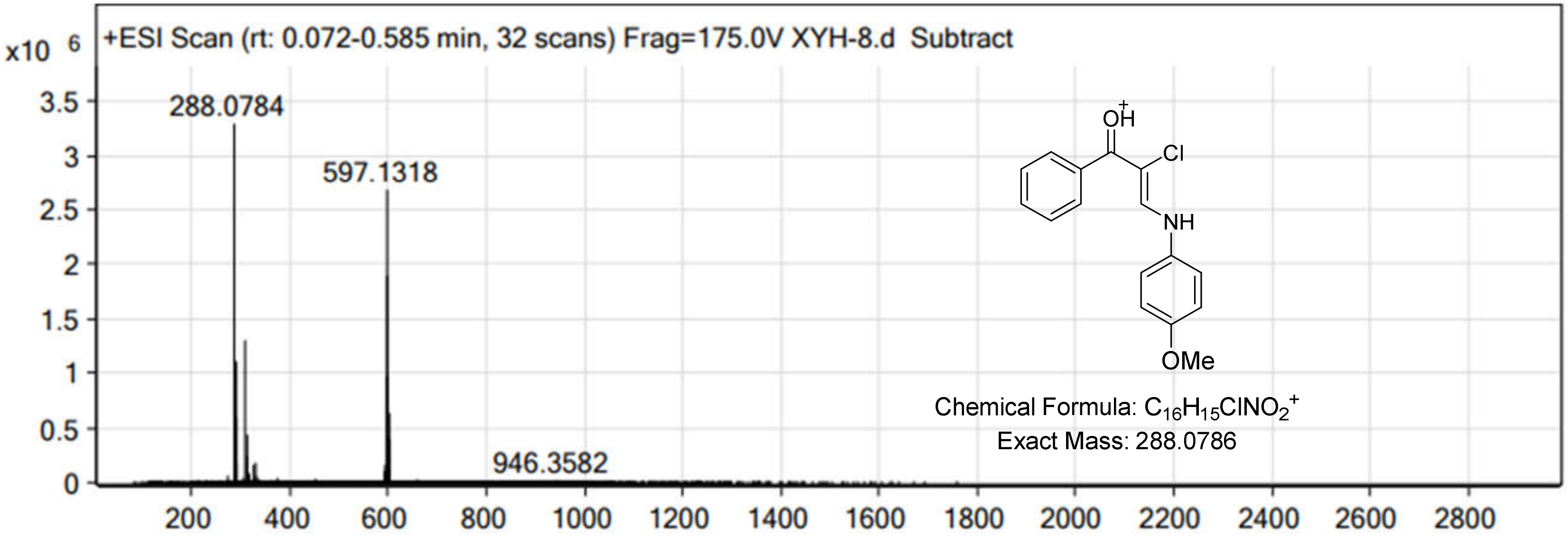

Figure S13. HRMS spectra of compound **2d**

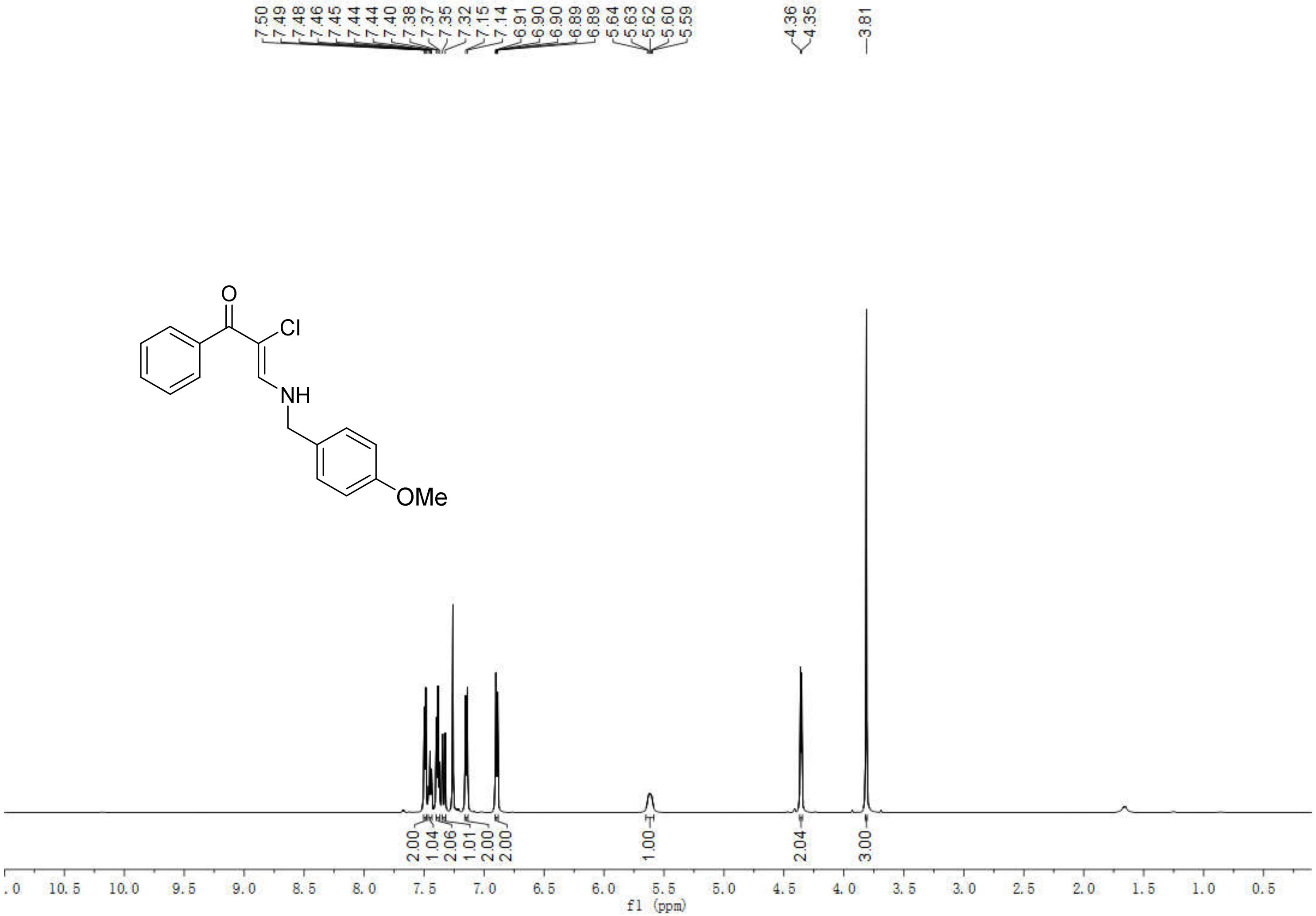

Figure S14. ^{1}H NMR (600 MHz, $CDCl_3$) spectra of compound **2e**

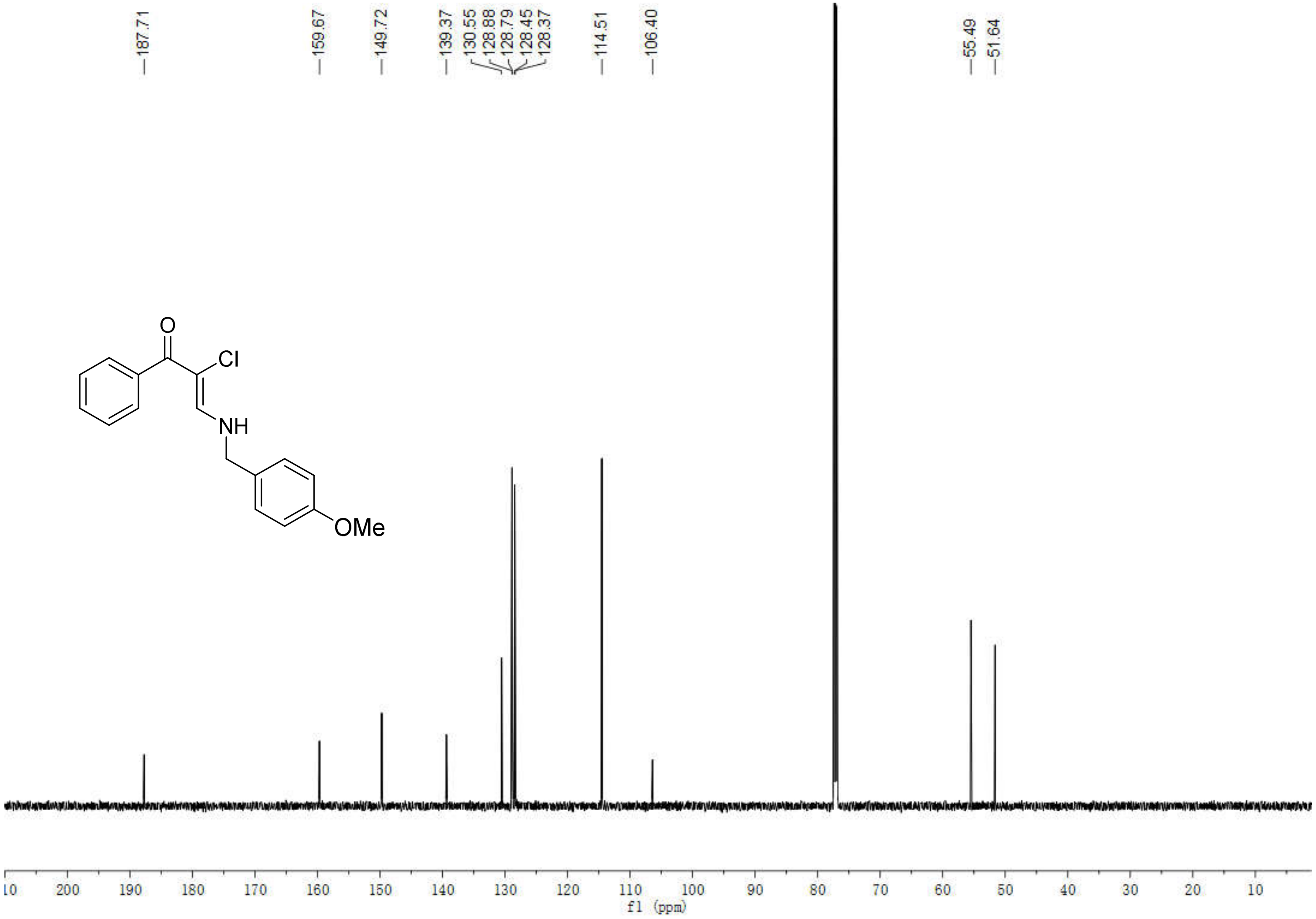

Figure S15. ^{13}C NMR (600 MHz, $CDCl_3$) spectra of compound **2e**

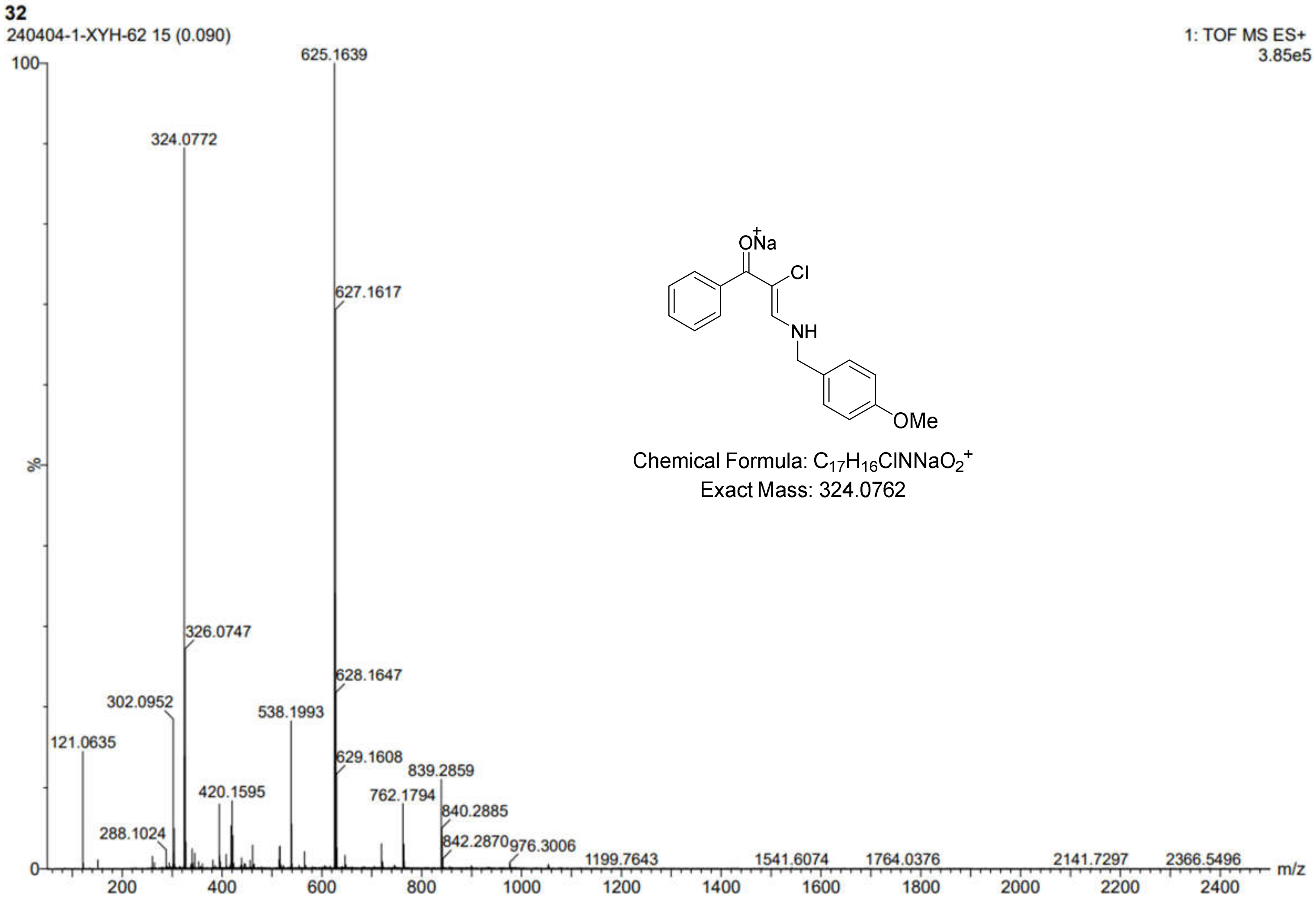

Figure S16. HRMS spectra of compound **2e**

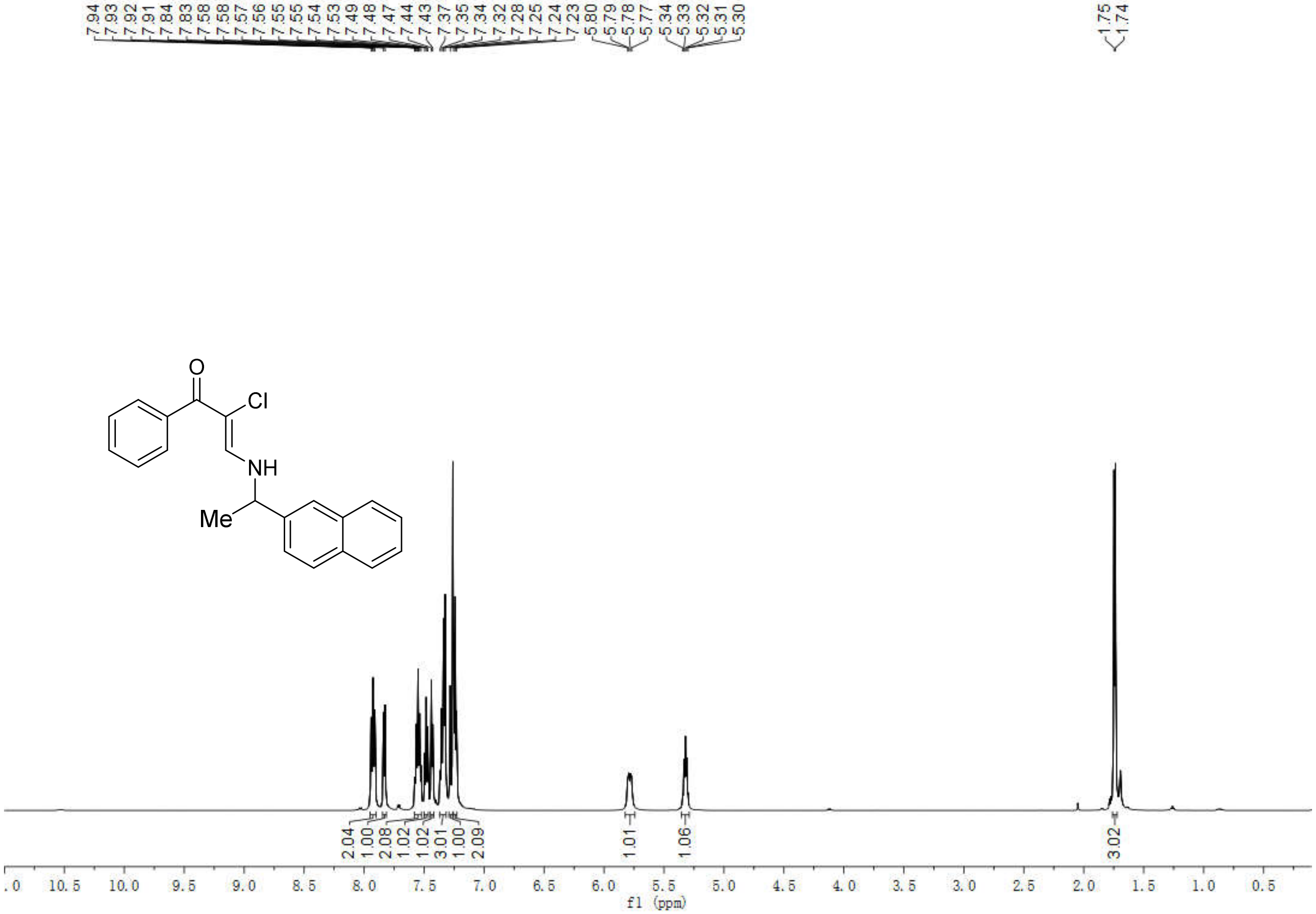

Figure S17. ^{1}H NMR (600 MHz, $CDCl_3$) spectra of compound **2f**

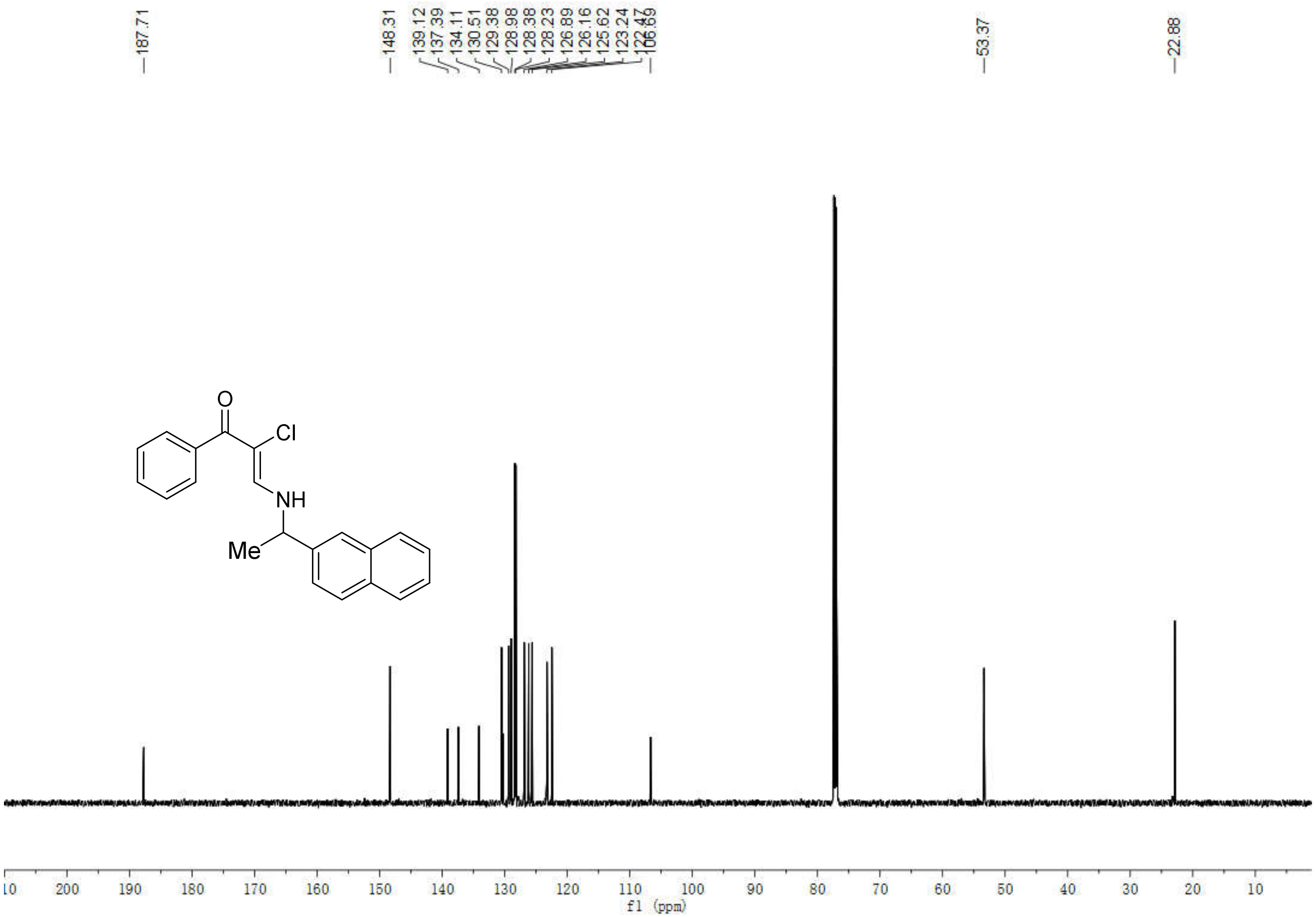

Figure S18. ^{13}C NMR (600 MHz, $CDCl_3$) spectra of compound **2f**

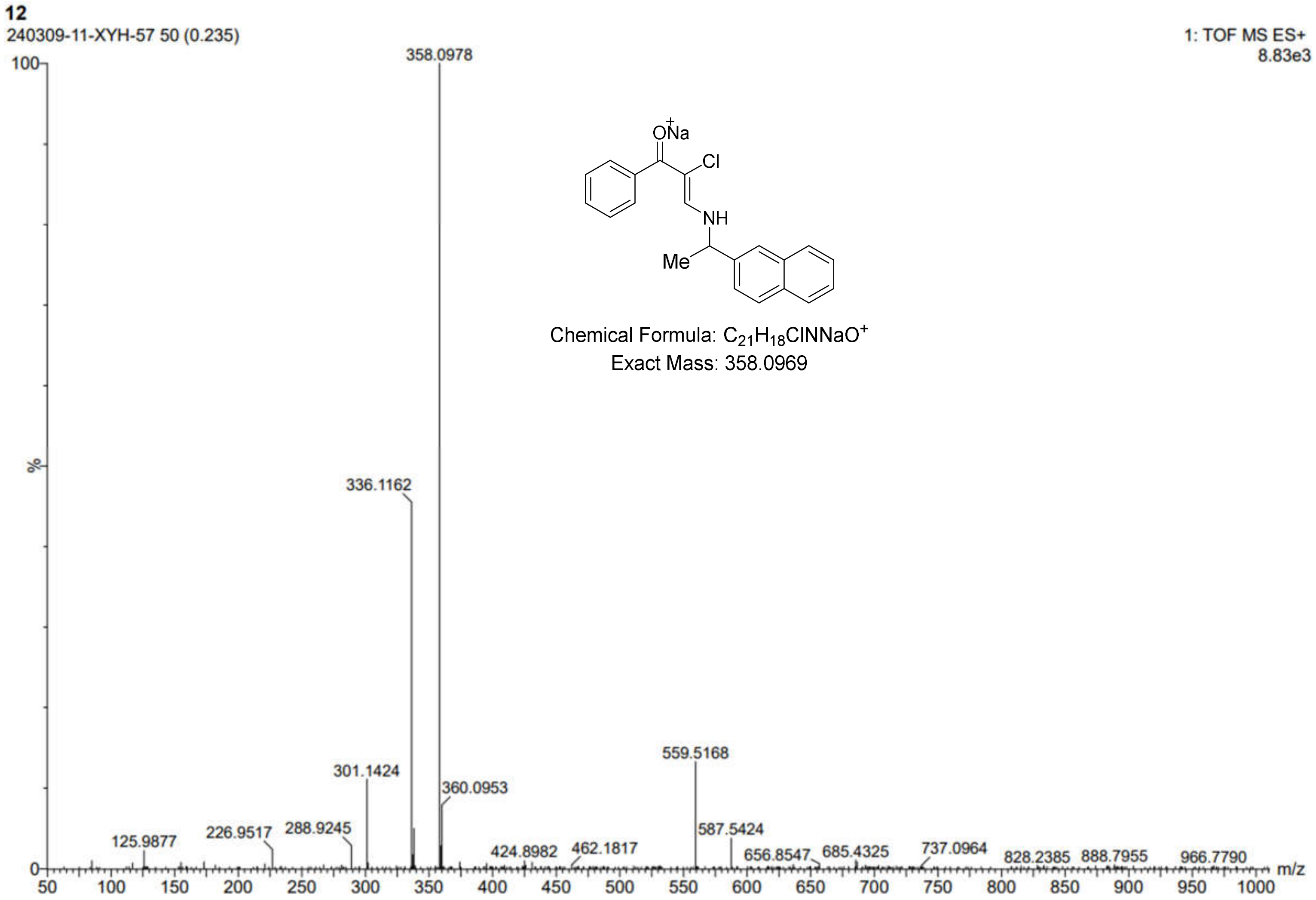

Figure S19. HRMS spectra of compound **2f**

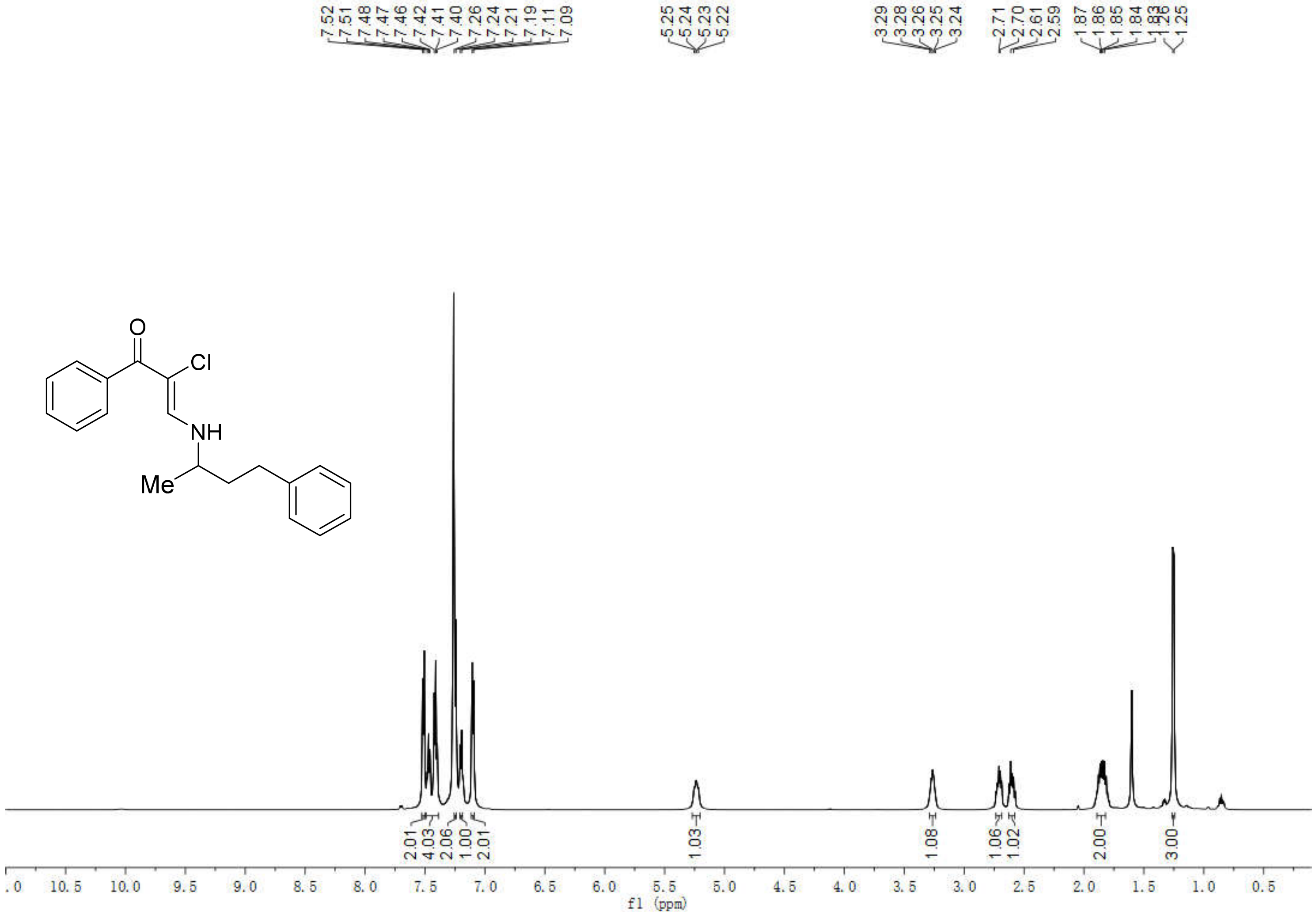

Figure S20. ^{1}H NMR (600 MHz, $CDCl_3$) spectra of compound **2g**

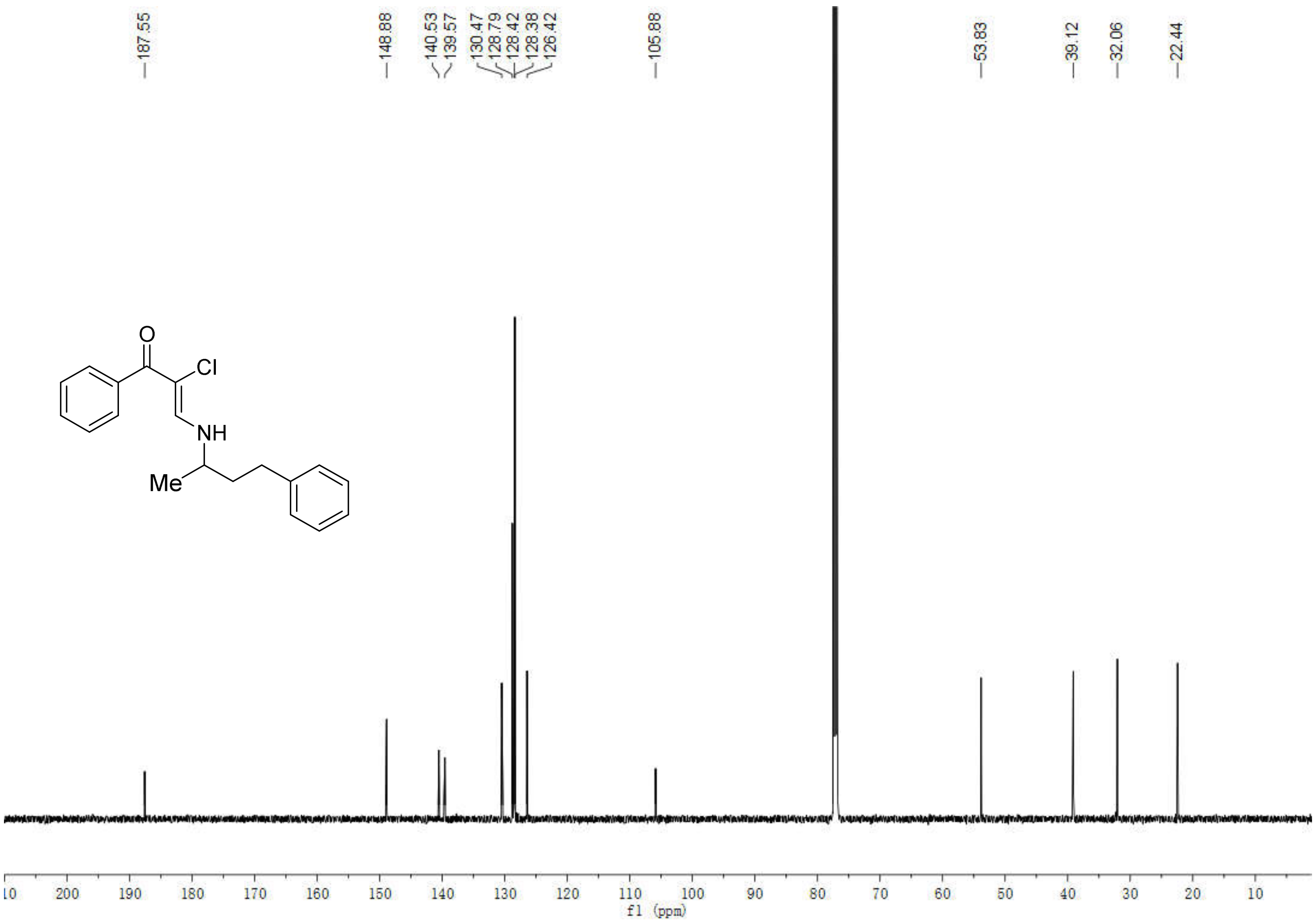

Figure S21. ^{13}C NMR (600 MHz, $CDCl_3$) spectra of compound **2g**

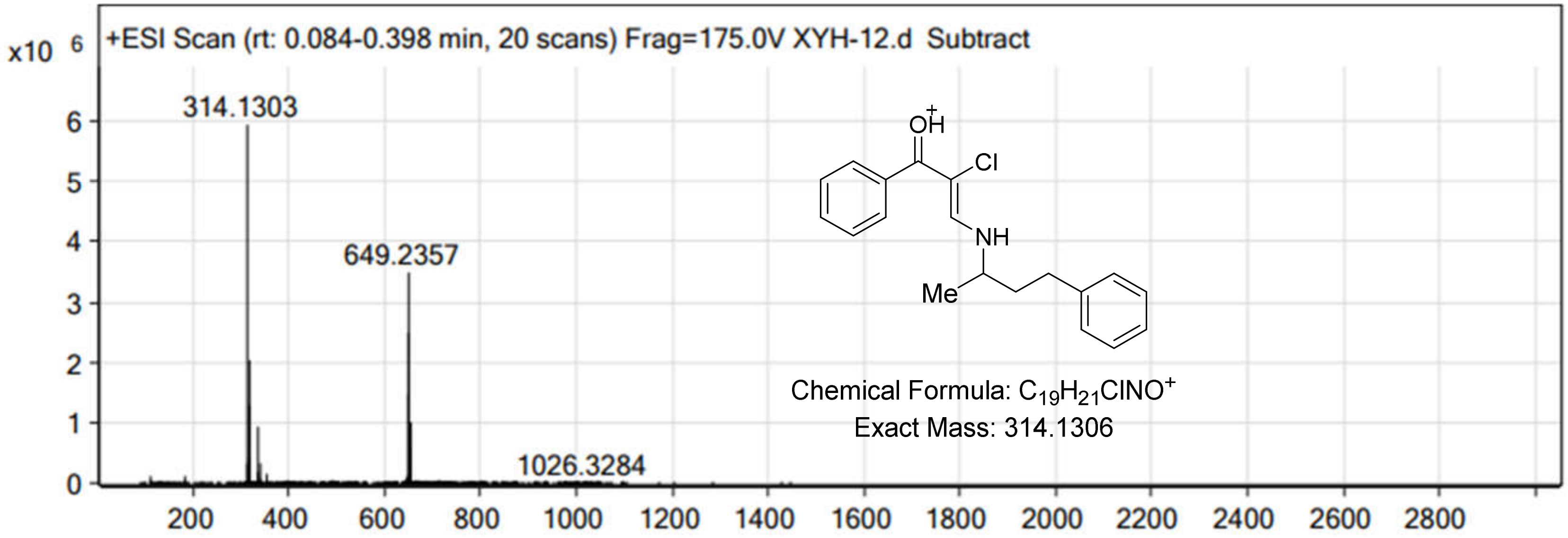

Figure S22. HRMS spectra of compound **2g**

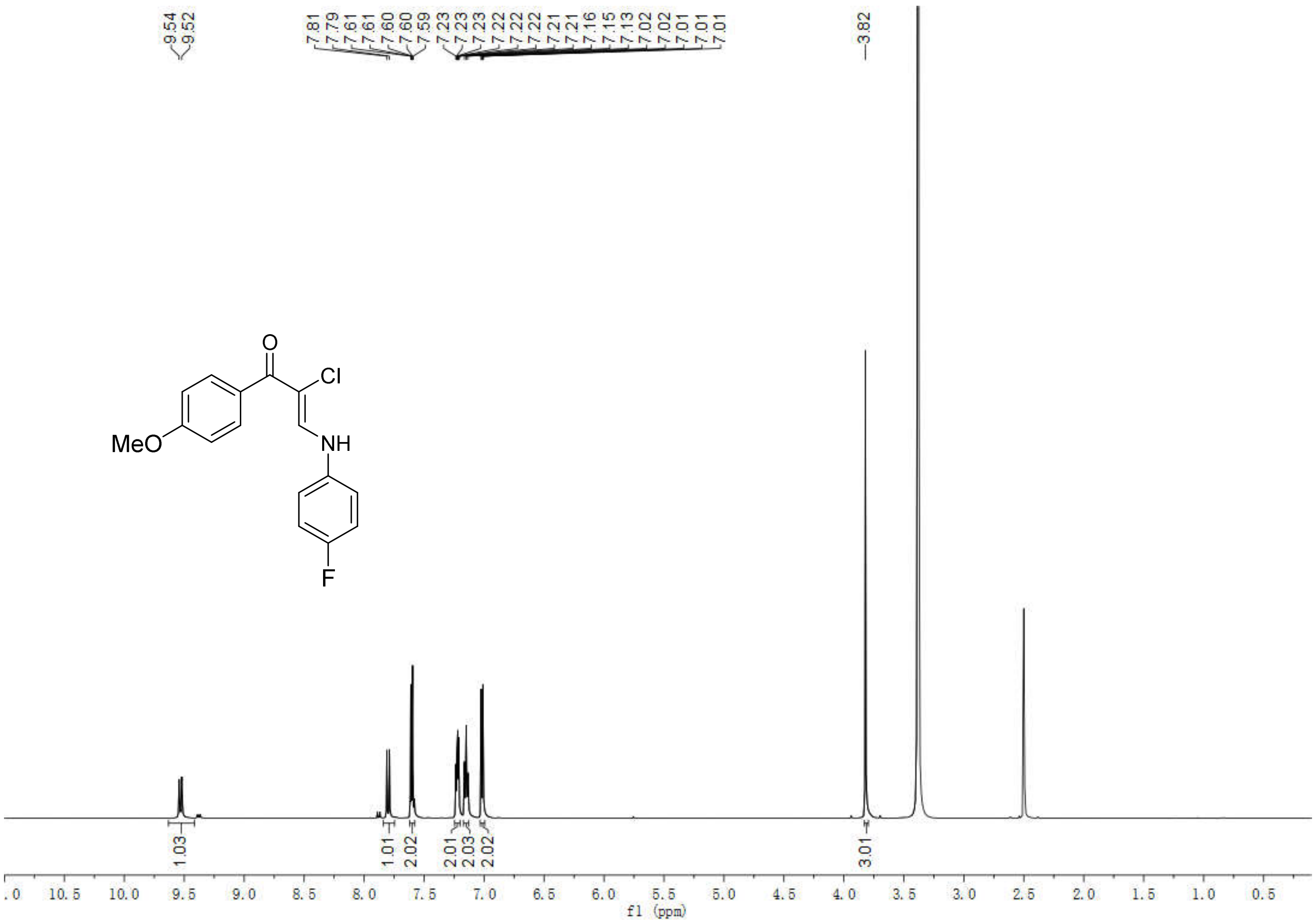

Figure S23. ^{1}H NMR (600 MHz, DMSO-d_6) spectra of compound **2h**

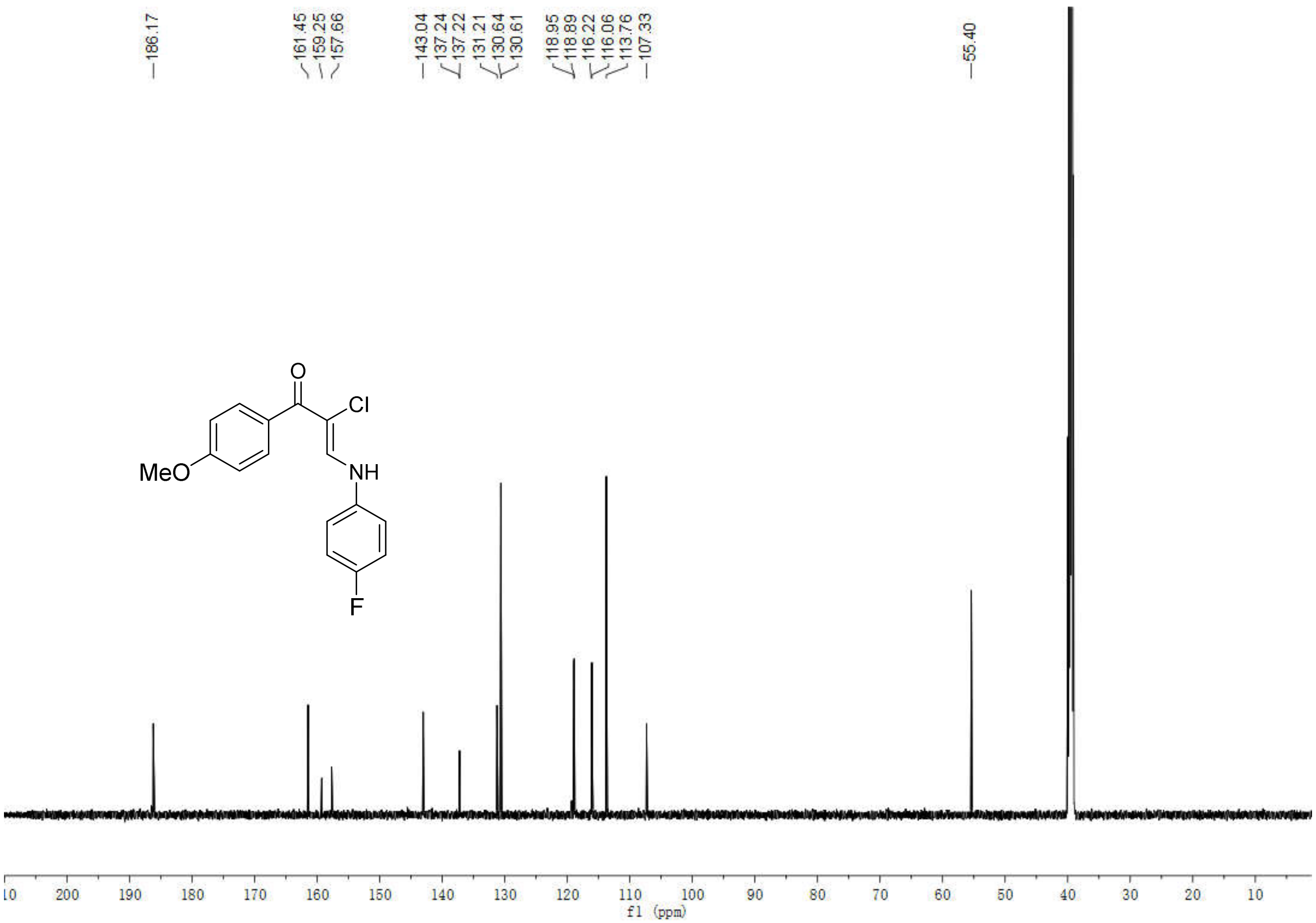

Figure S24. ^{13}C NMR (600 MHz, DMSO-d_6) spectra of compound **2h**

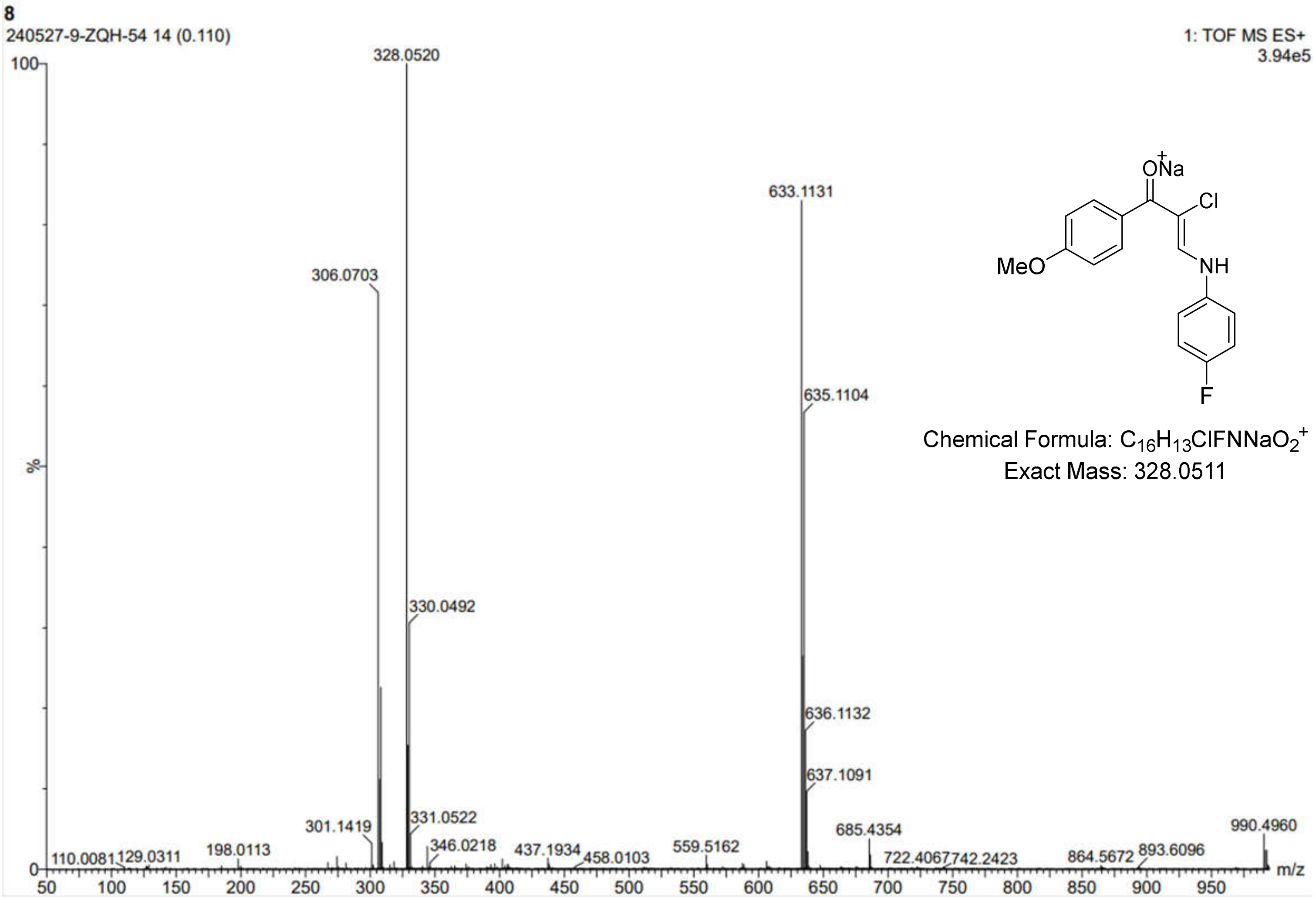

Figure S25. HRMS spectra of compound **2h**

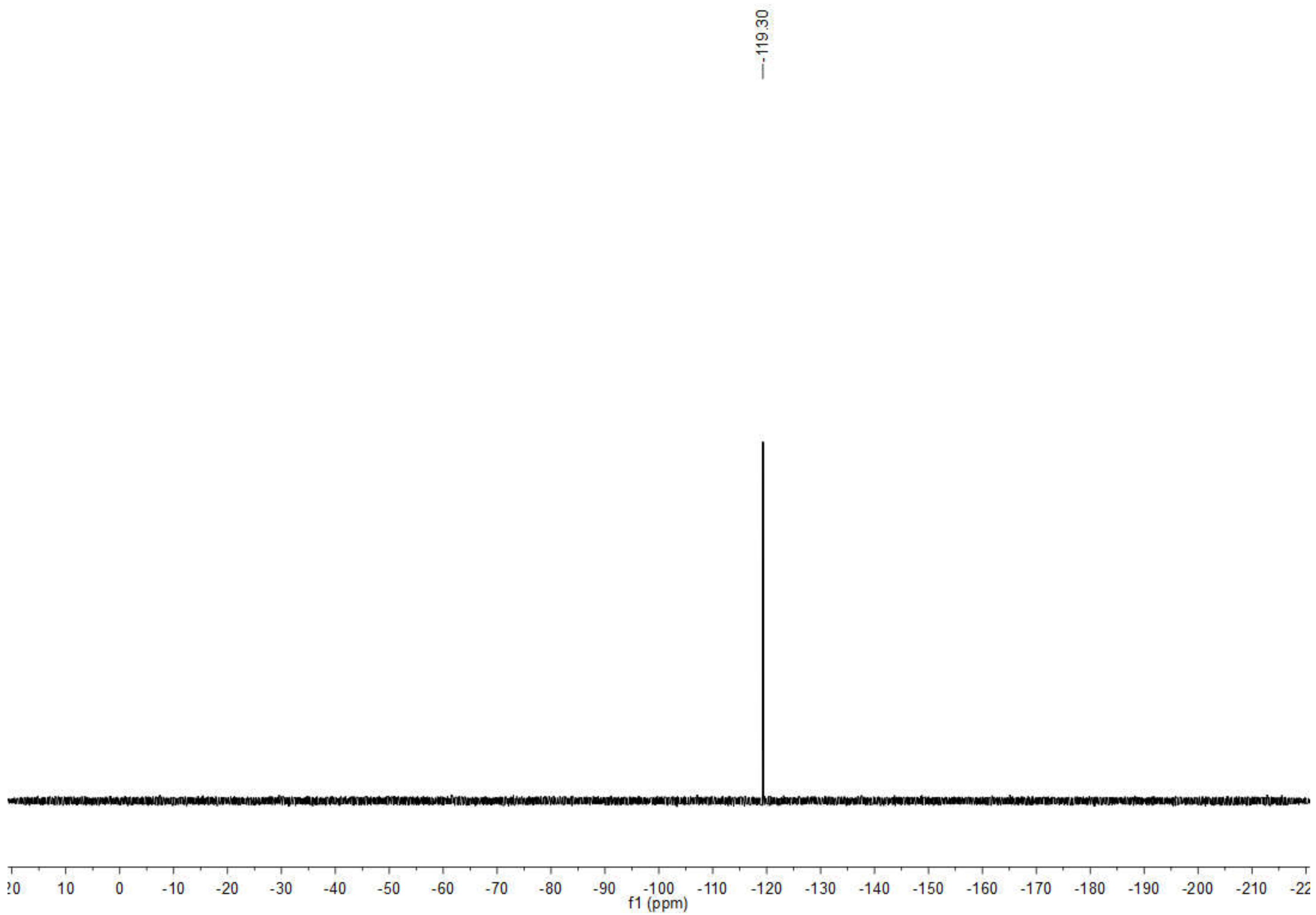

Figure S26. ^{19}F NMR (500 MHz, DMSO-d_6) spectra of compound **2h**

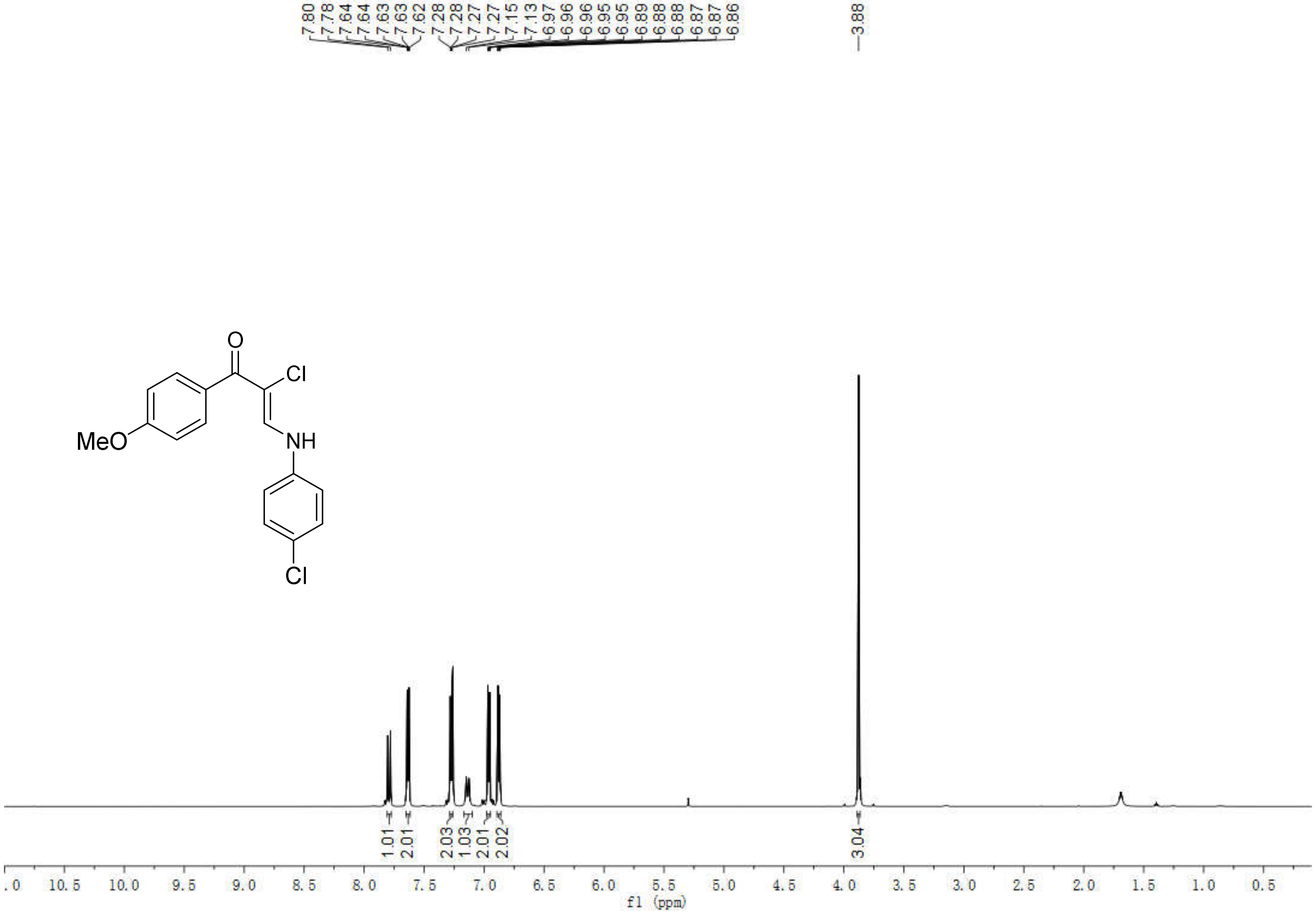

Figure S27. ^{1}H NMR (600 MHz, $CDCl_3$) spectra of compound **2i**

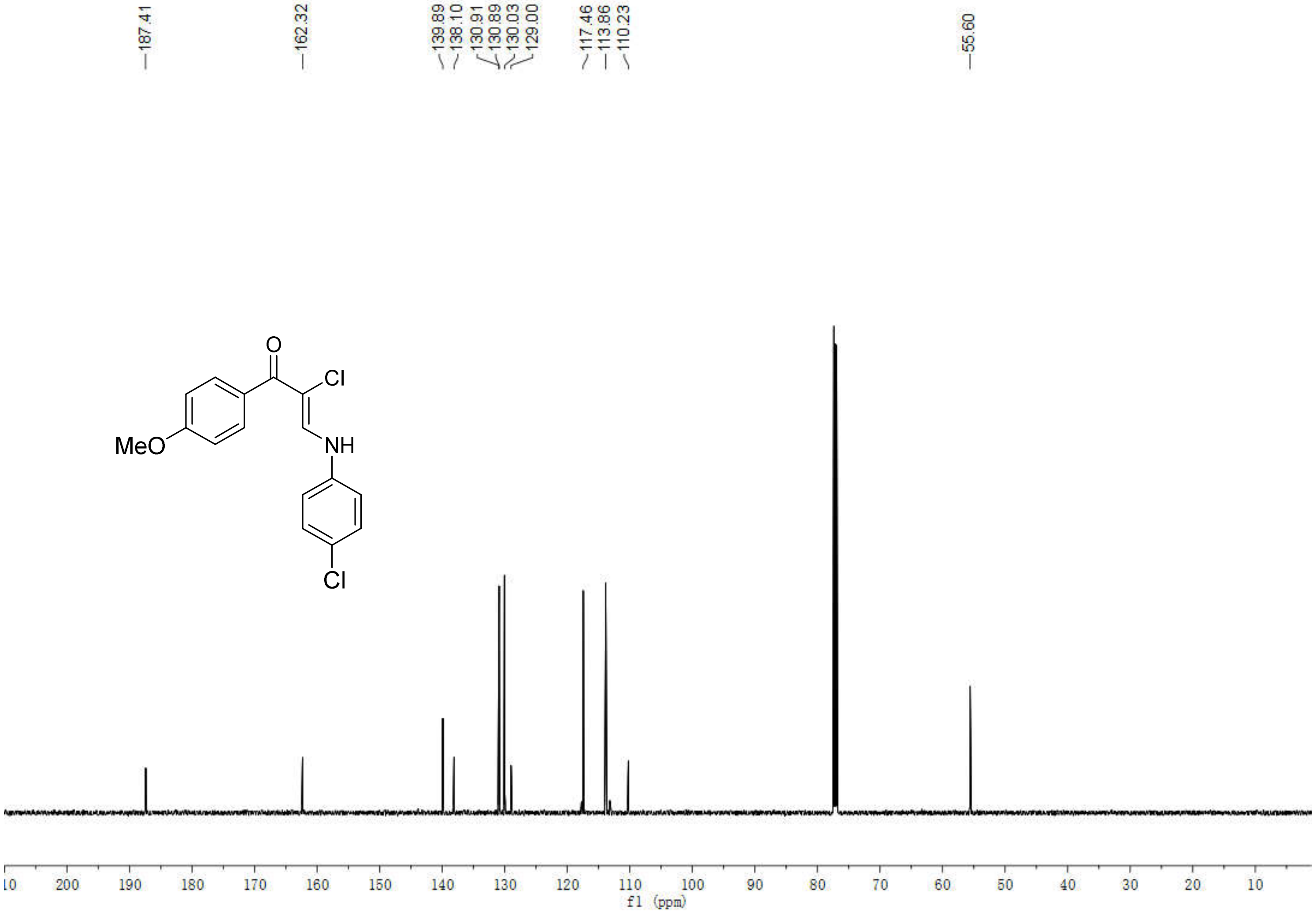

Figure S28. ^{13}C NMR (600 MHz, $CDCl_3$) spectra of compound **2i**

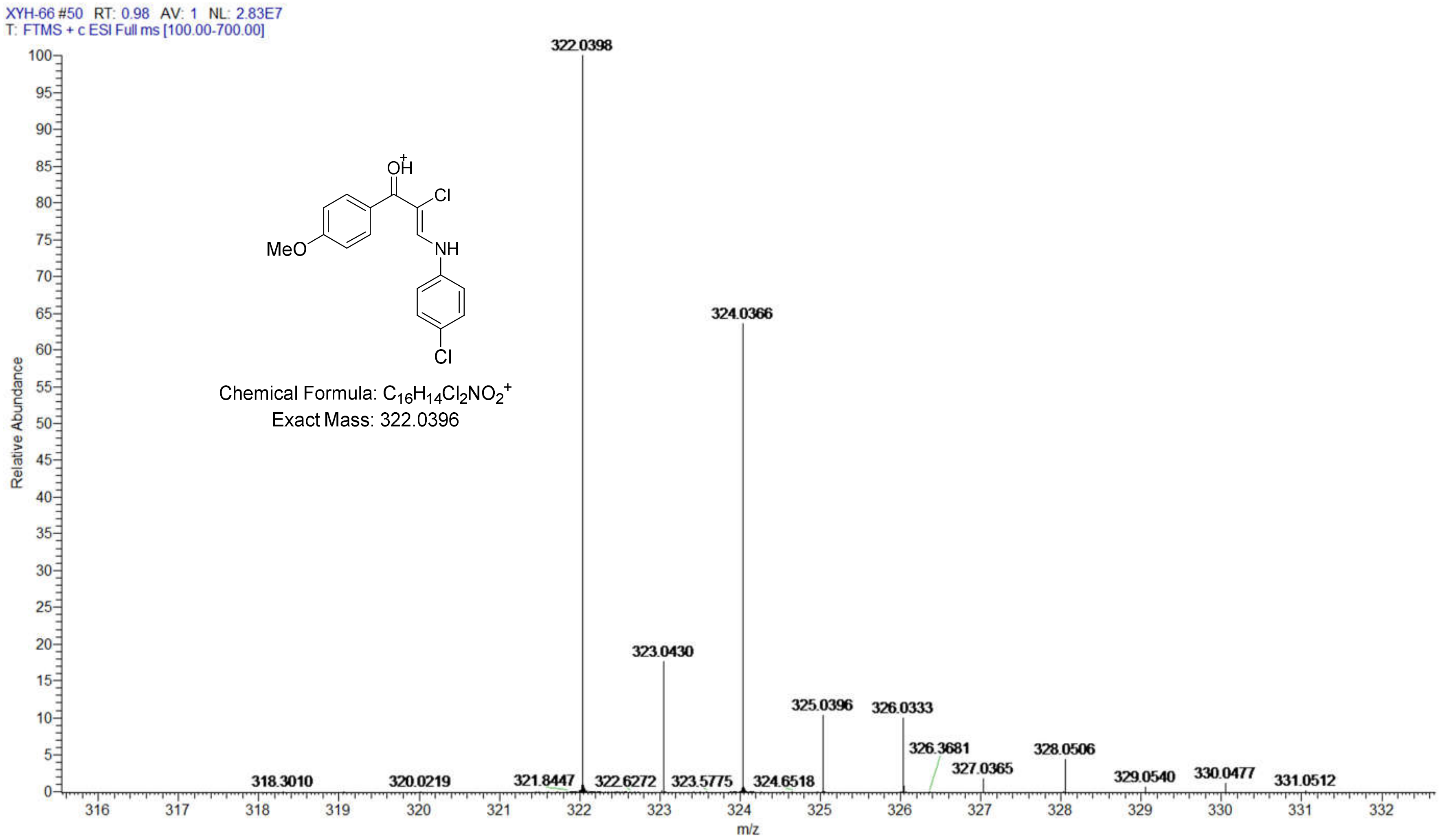

Figure S29. HRMS spectra of compound **2i**

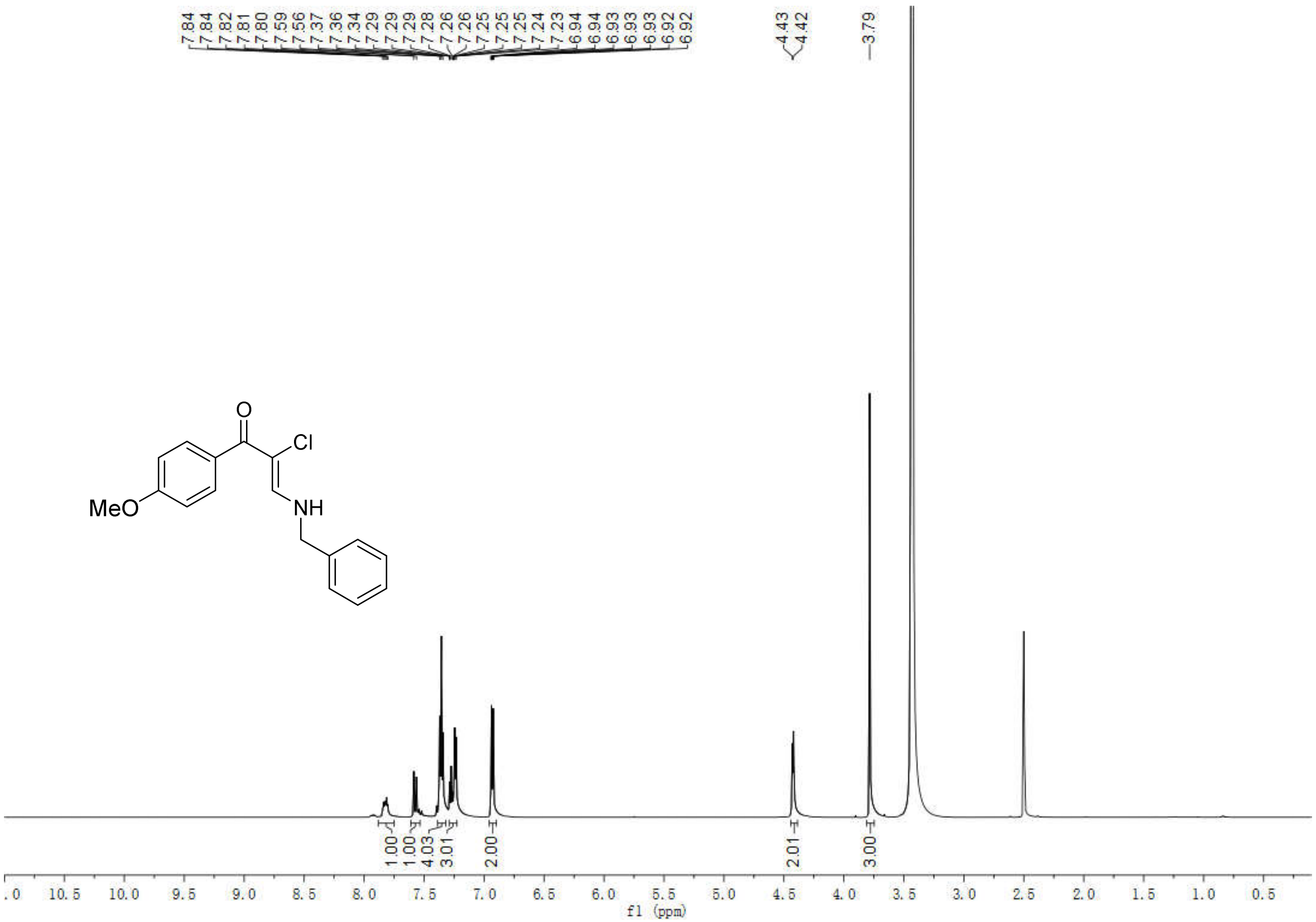

Figure S30. ¹H NMR (600 MHz, DMSO-d_6) spectra of compound **2j**

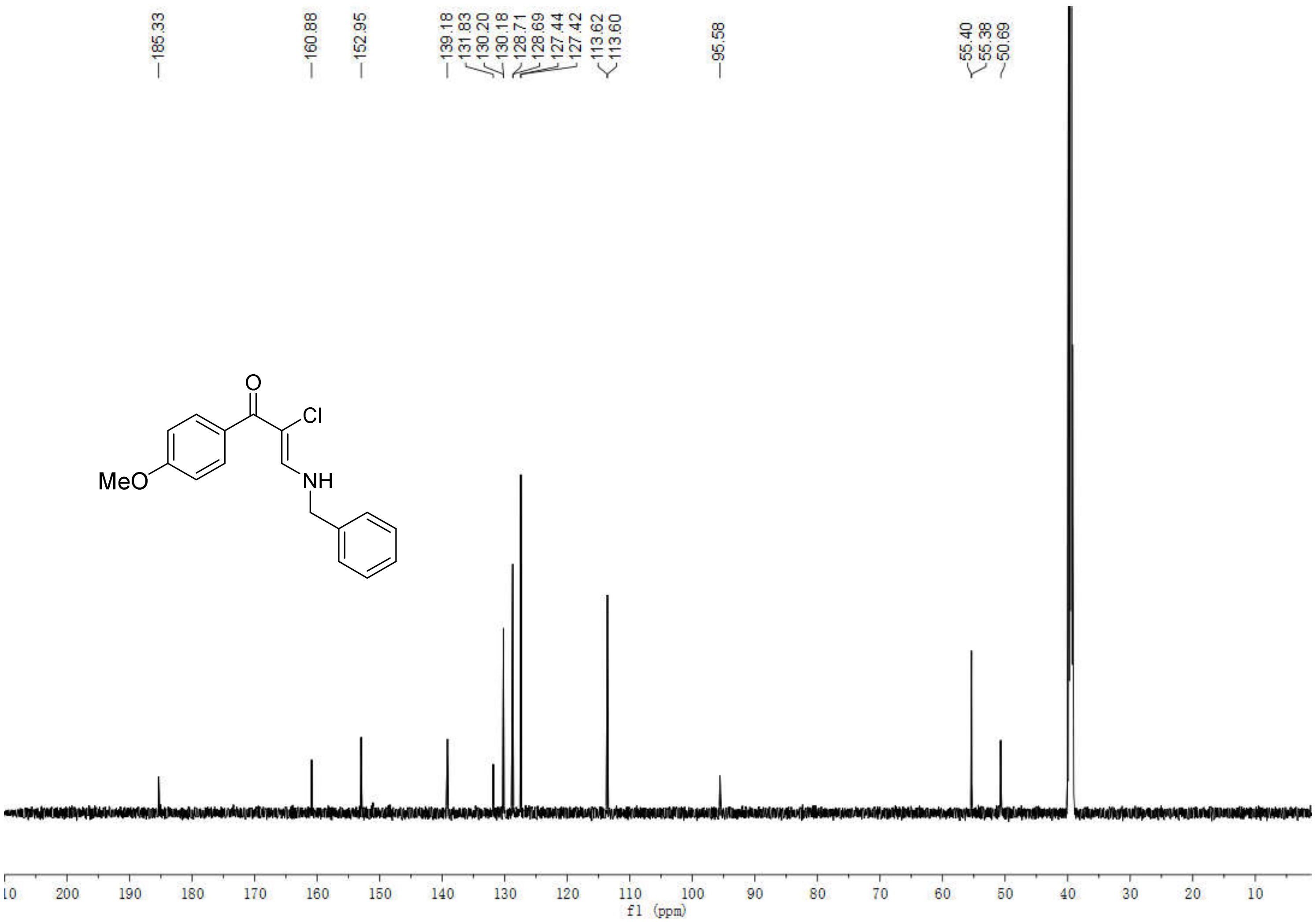

Figure S31. ^{13}C NMR (600 MHz, DMSO-d_6) spectra of compound **2j**

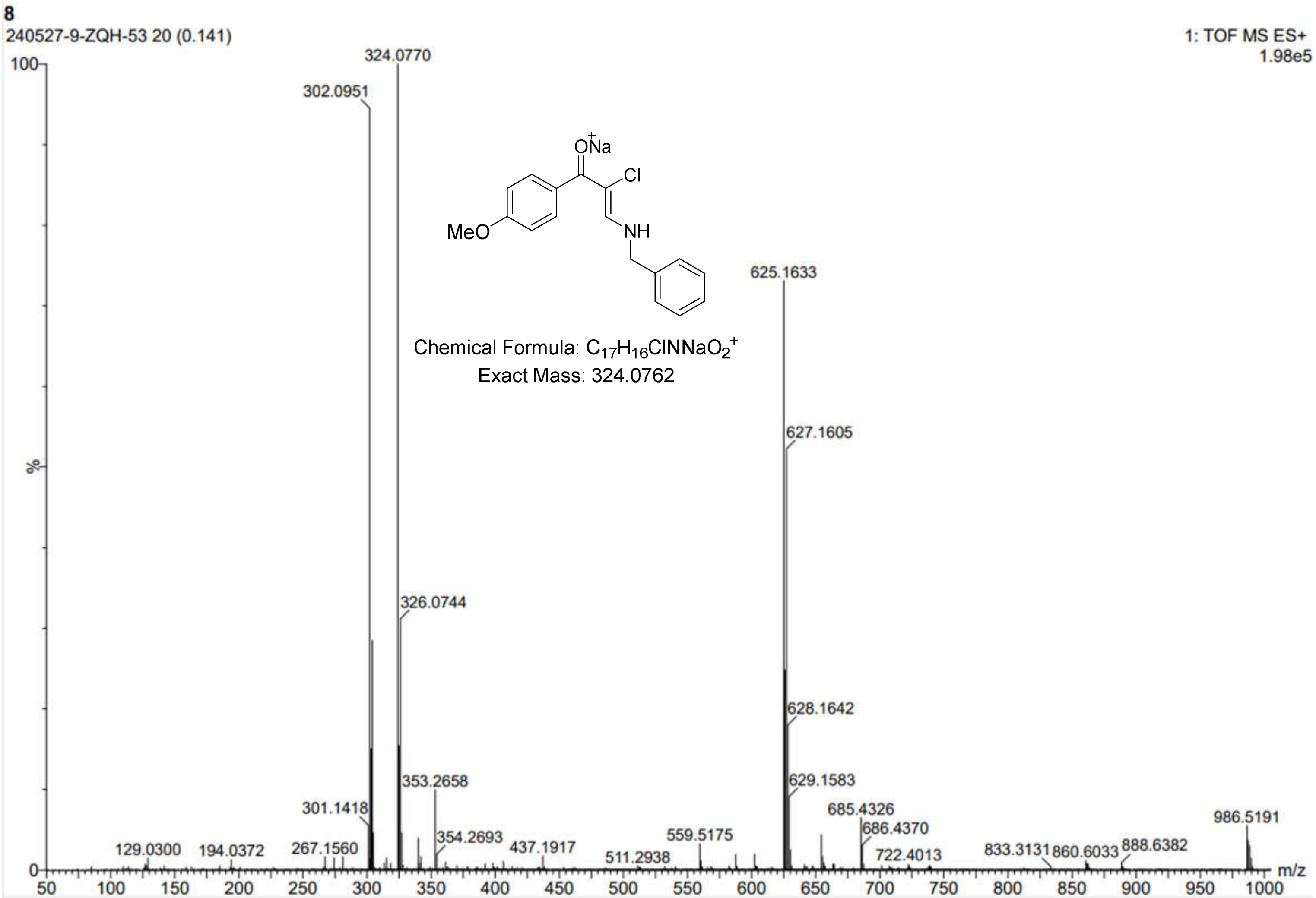

Figure S32. HRMS spectra of compound **2j**

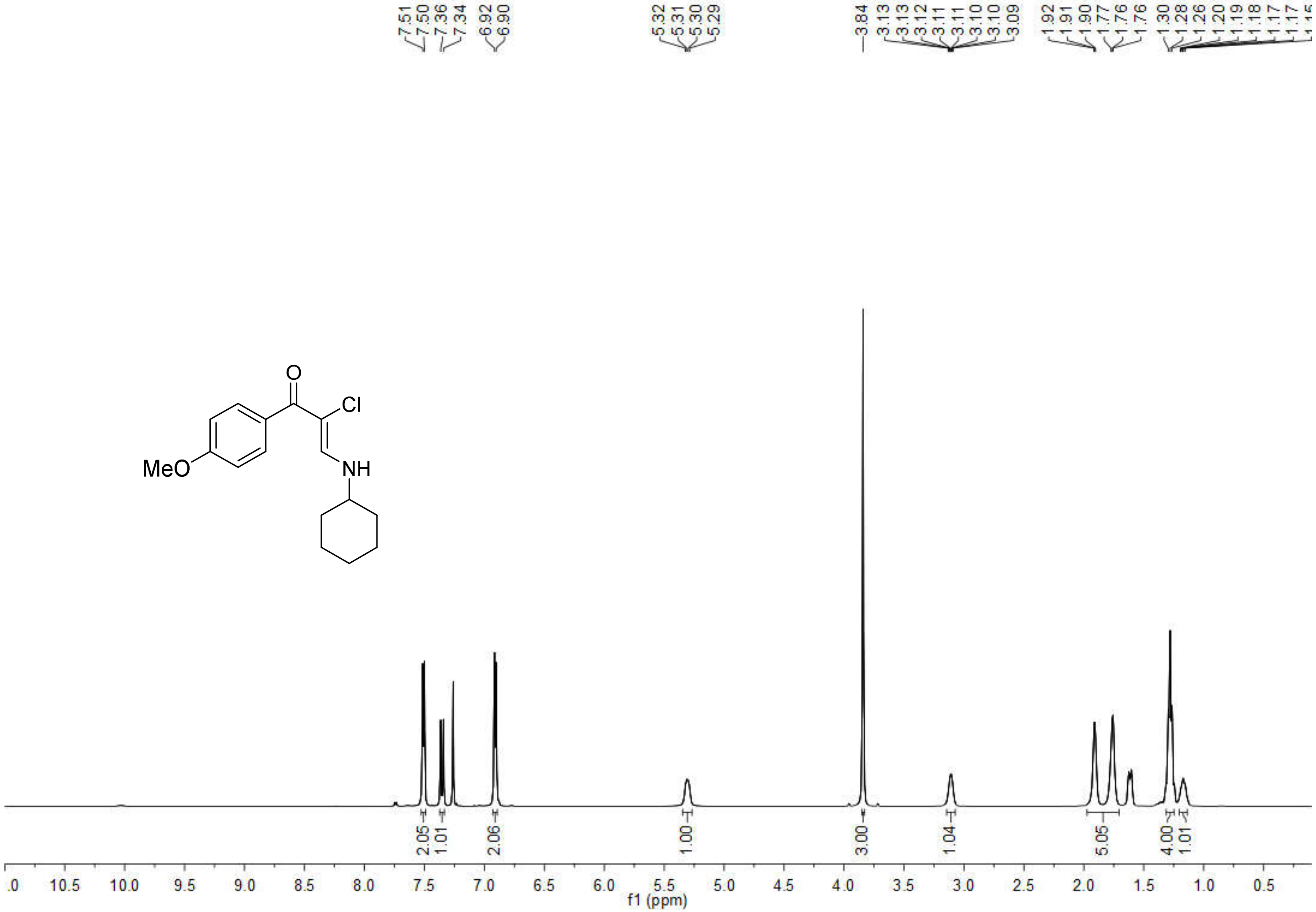

Figure S33. ^{1}H NMR (600 MHz, $CDCl_3$) spectra of compound **2k**

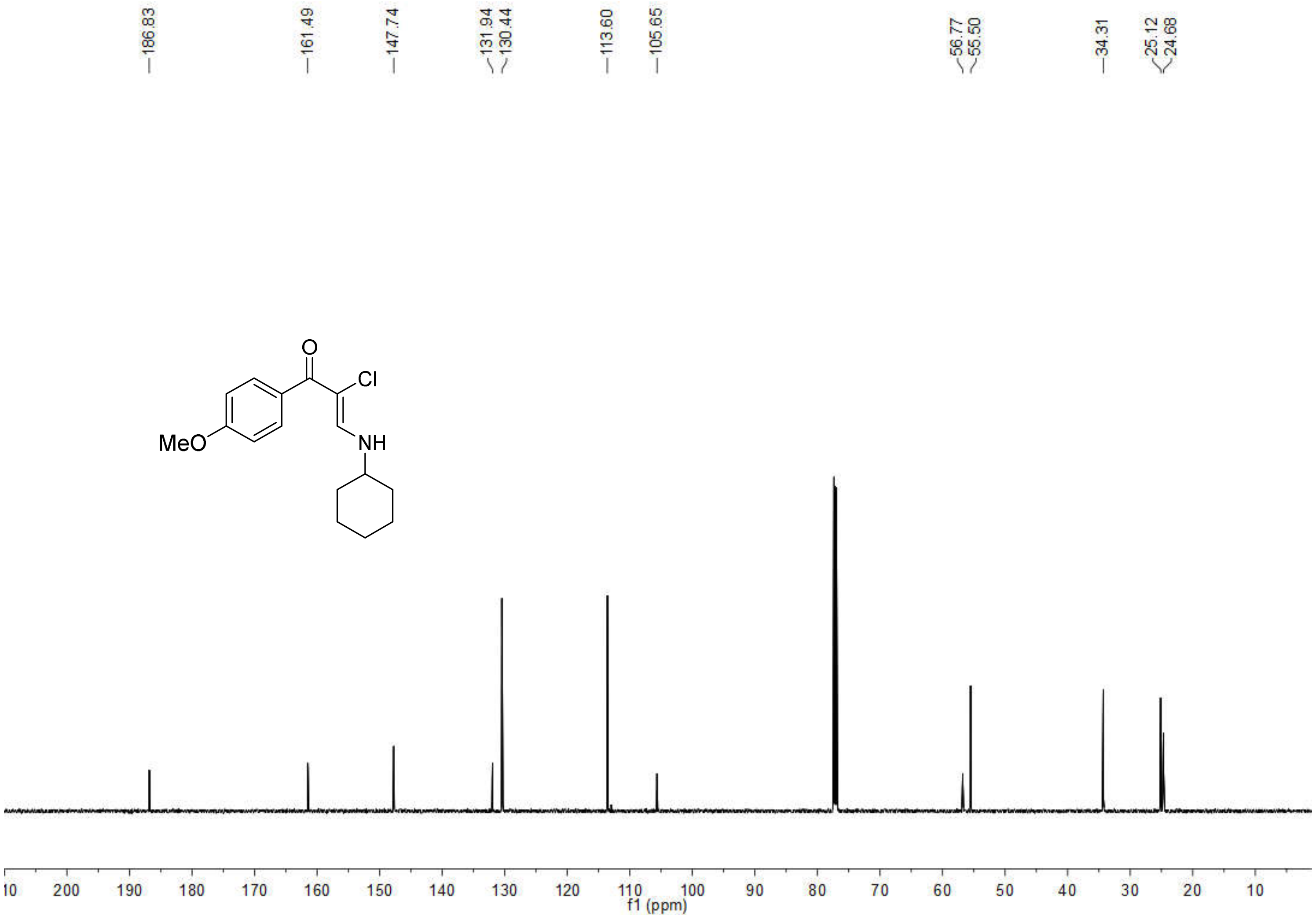

Figure S34. ^{13}C NMR (600 MHz, $CDCl_3$) spectra of compound **2k**

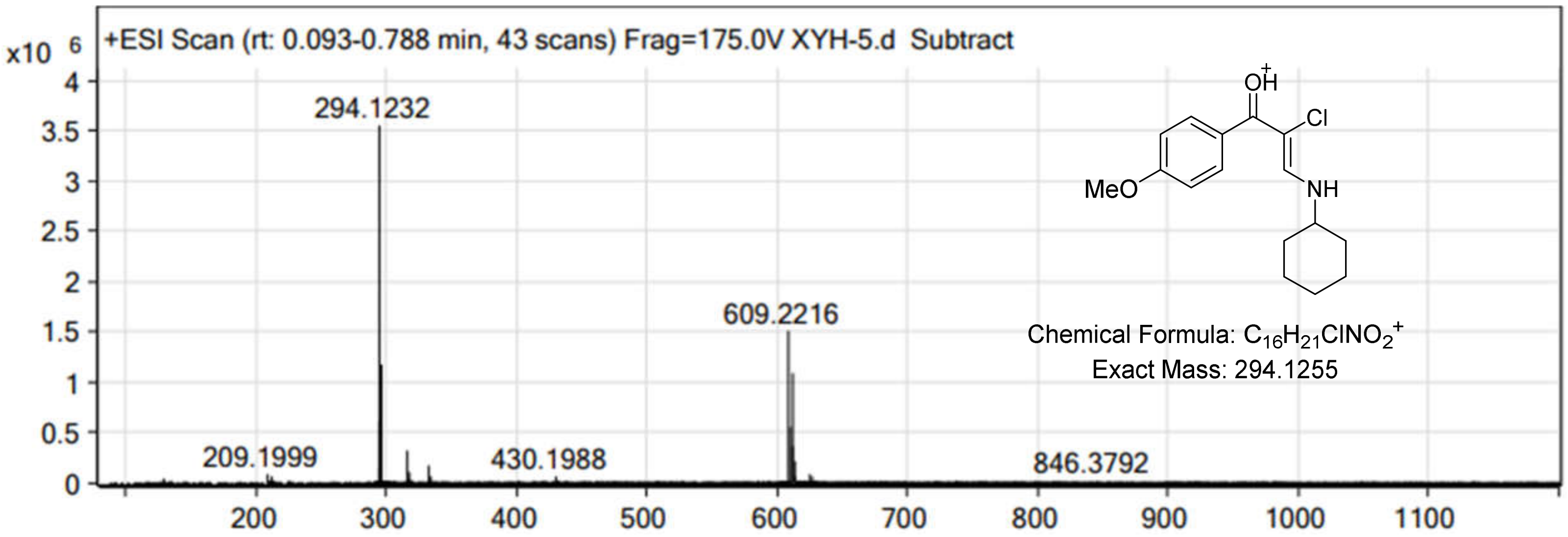

Figure S35. HRMS spectra of compound **2k**

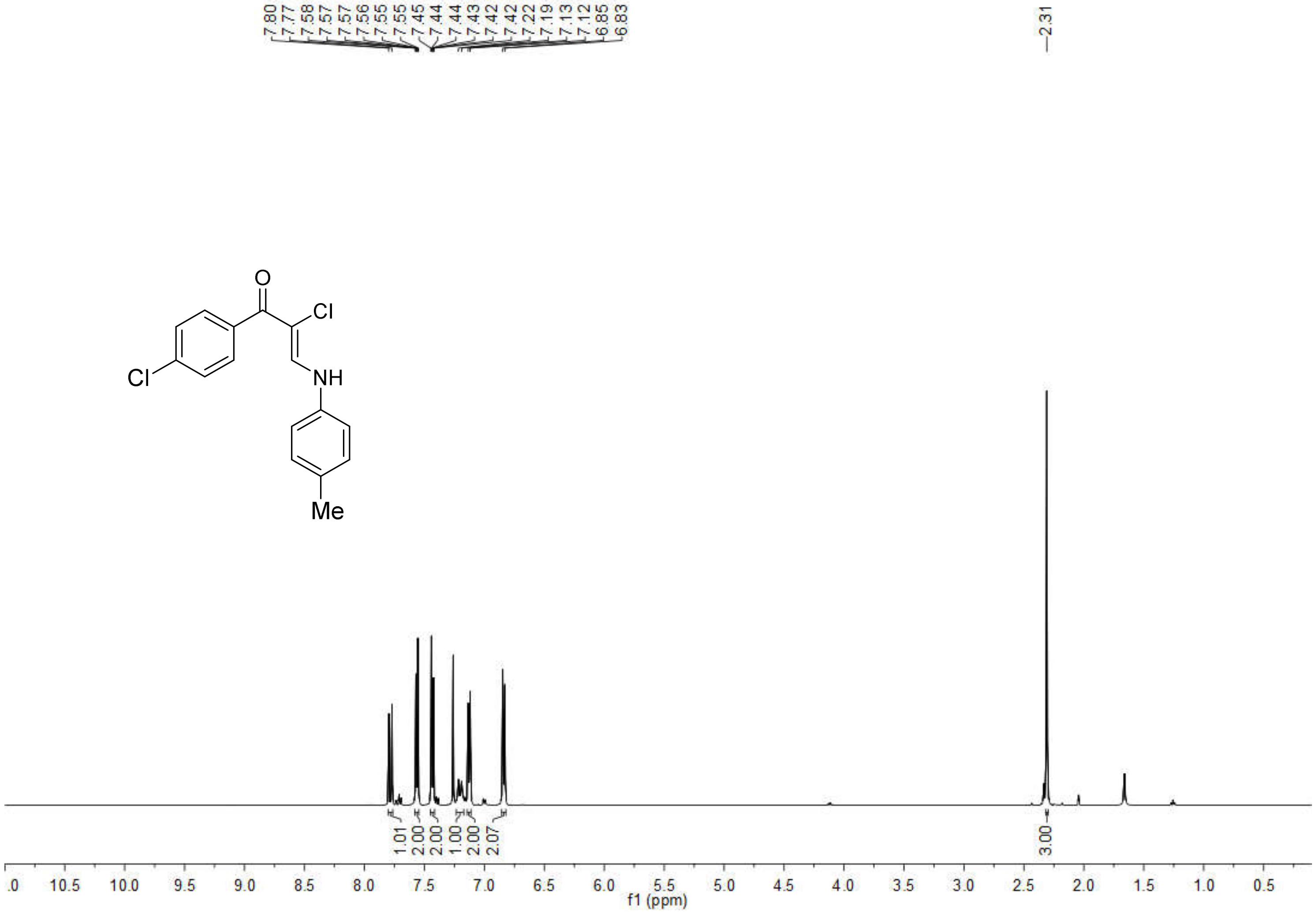

Figure S36. ^{1}H NMR (500 MHz, $CDCl_3$) spectra of compound **2l**

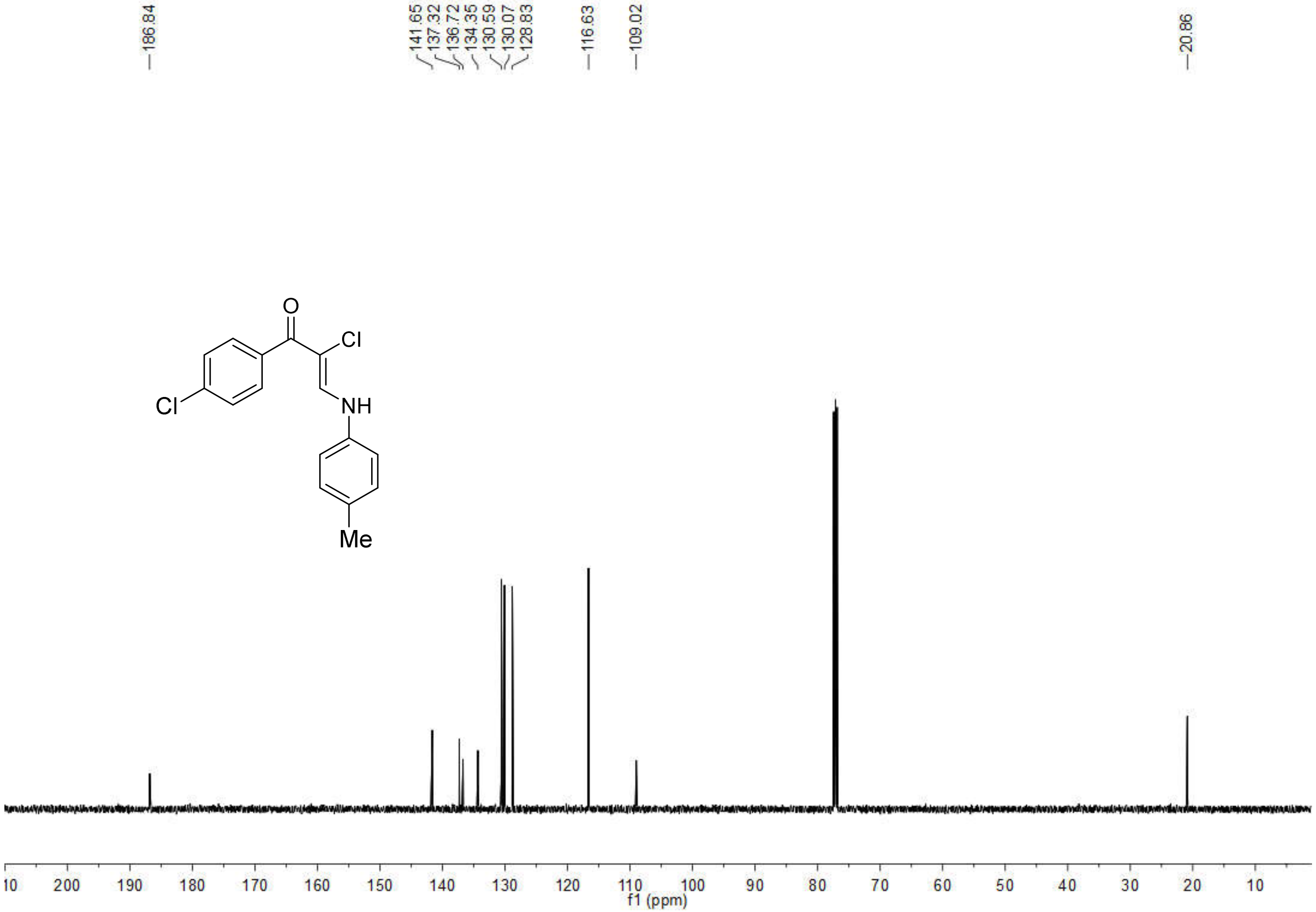

Figure S37. ^{13}C NMR (500 MHz, $CDCl_3$) spectra of compound **2l**

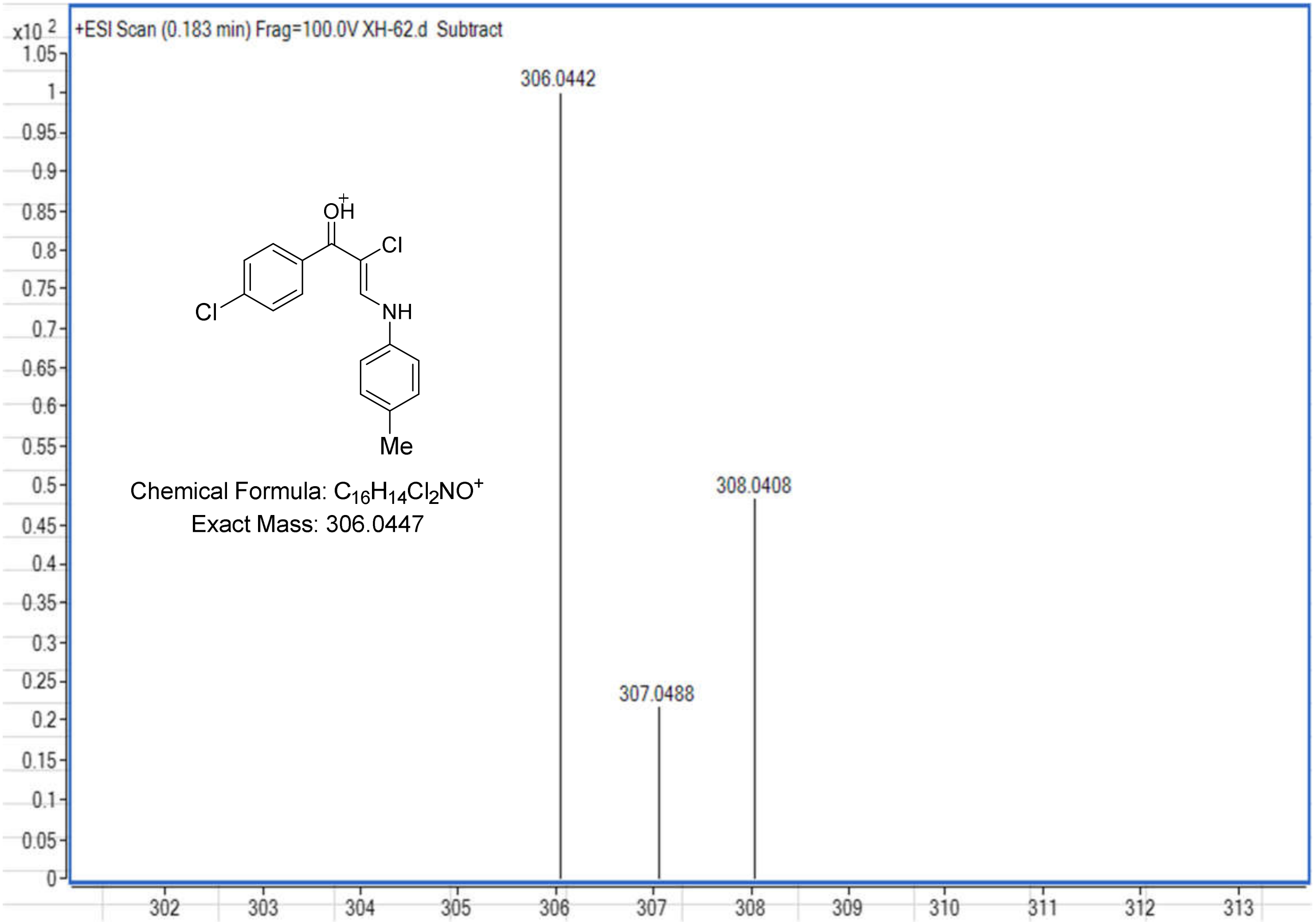

Figure S38. HRMS spectra of compound **2l**

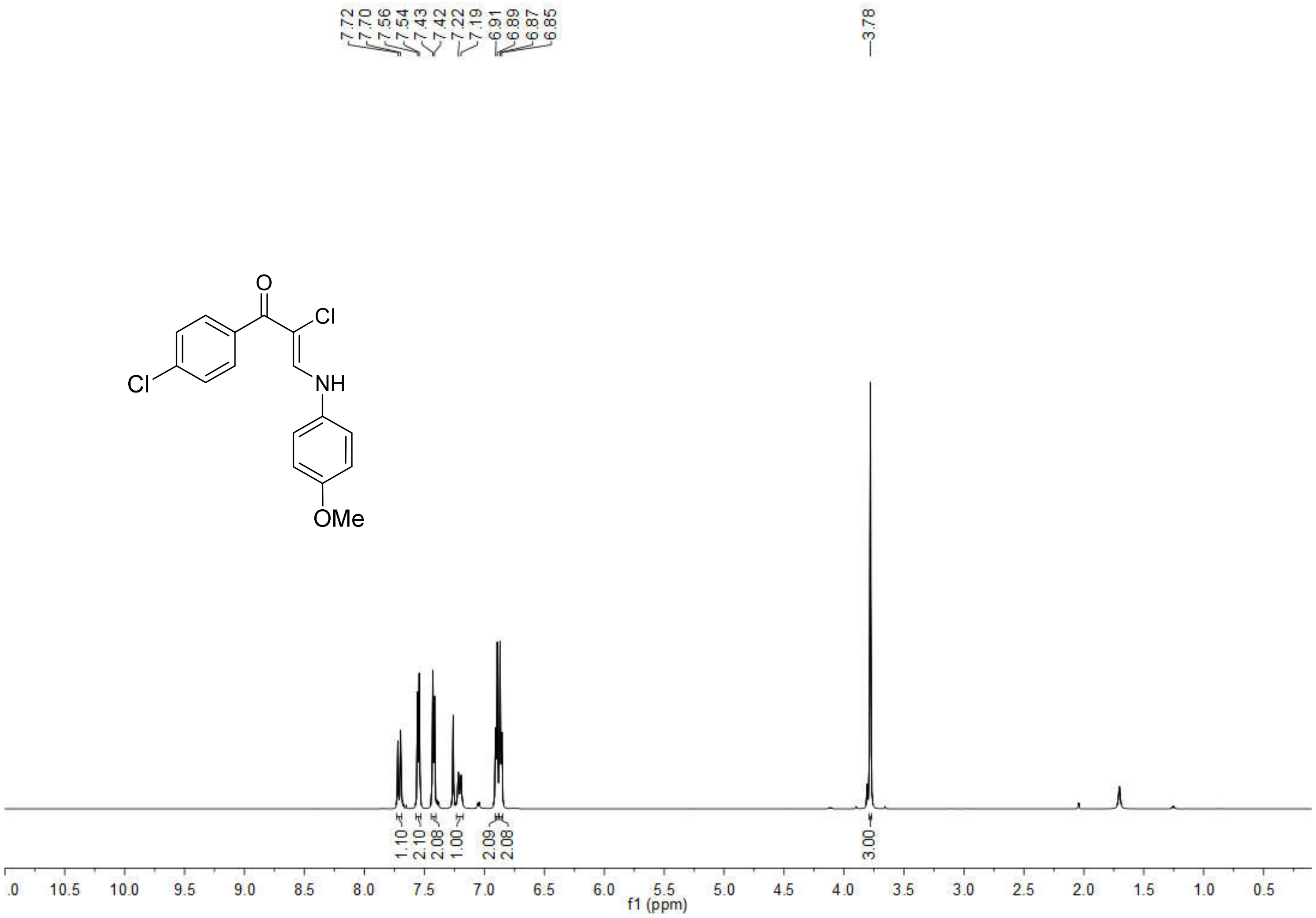

Figure S39. ^{1}H NMR (600 MHz, $CDCl_3$) spectra of compound **2m**

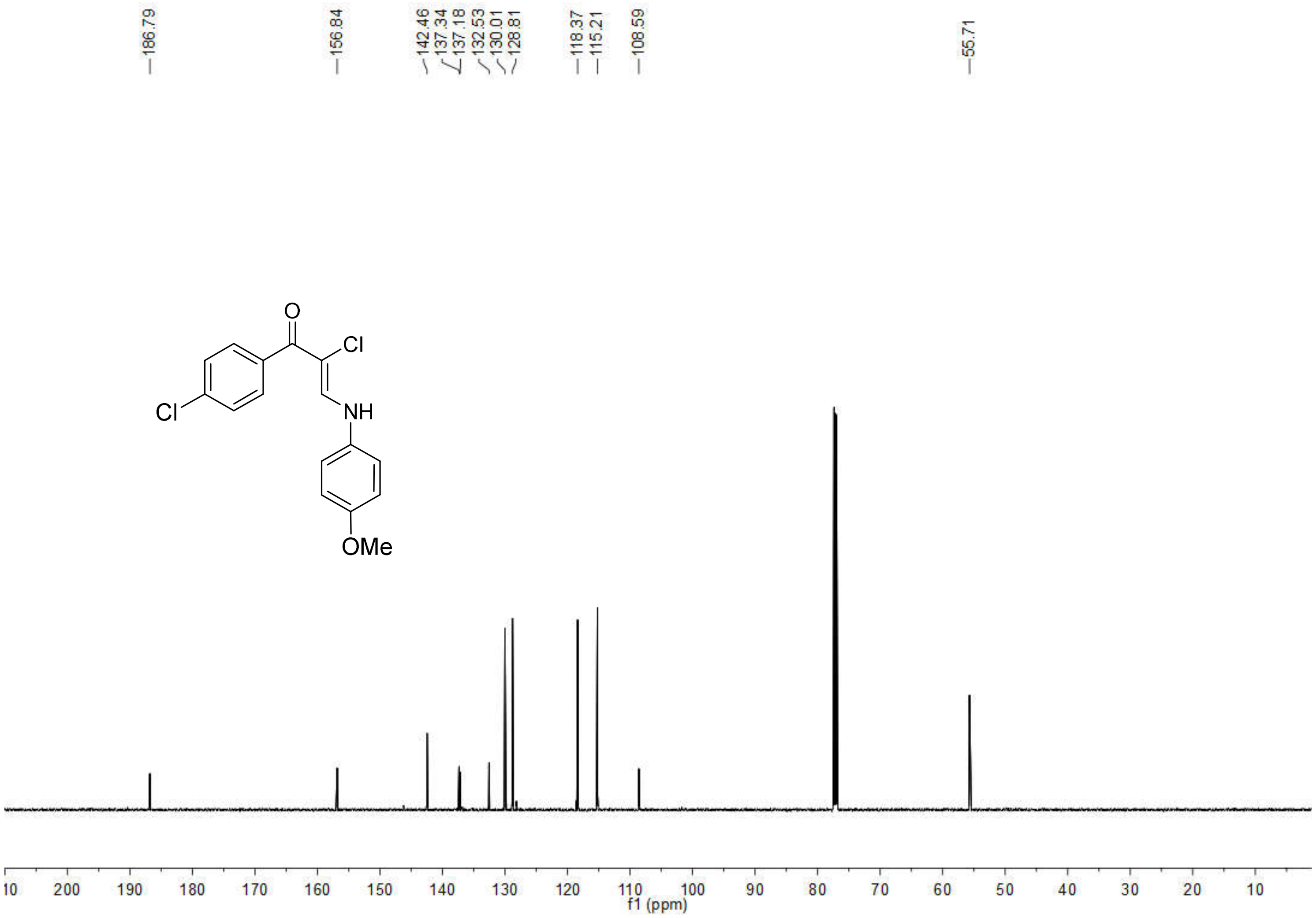

Figure S40. ^{13}C NMR (600 MHz, $CDCl_3$) spectra of compound **2m**

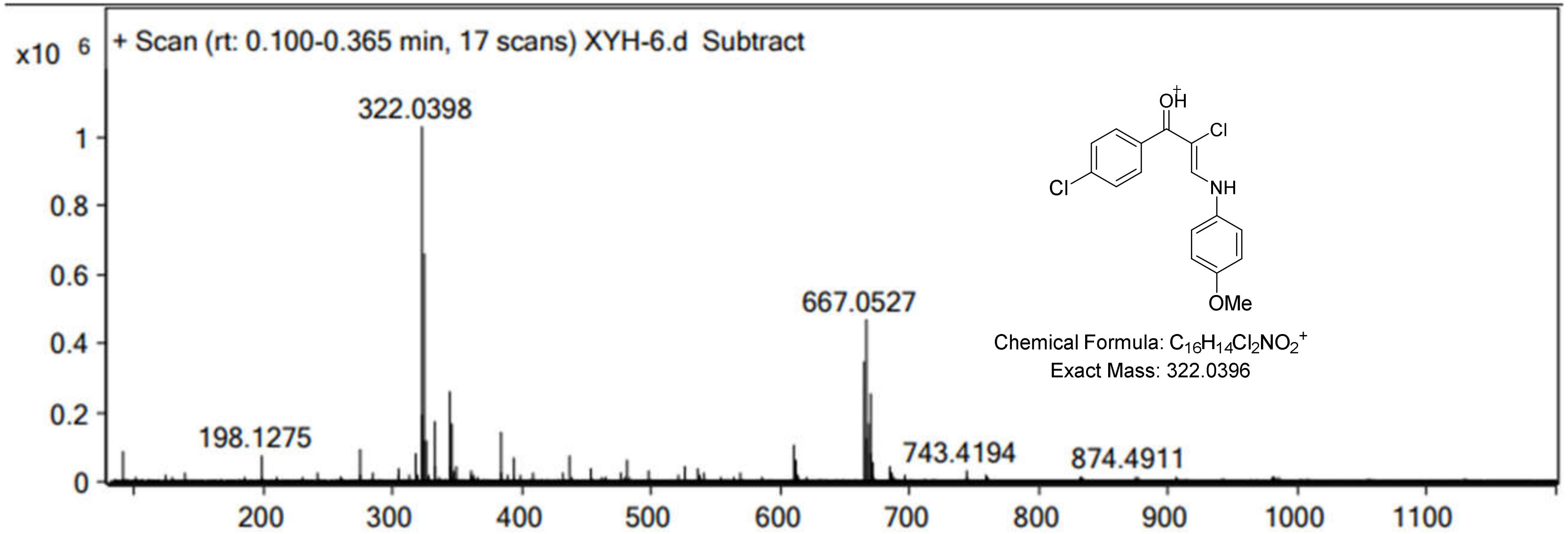

Figure S41. HRMS spectra of compound **2m**

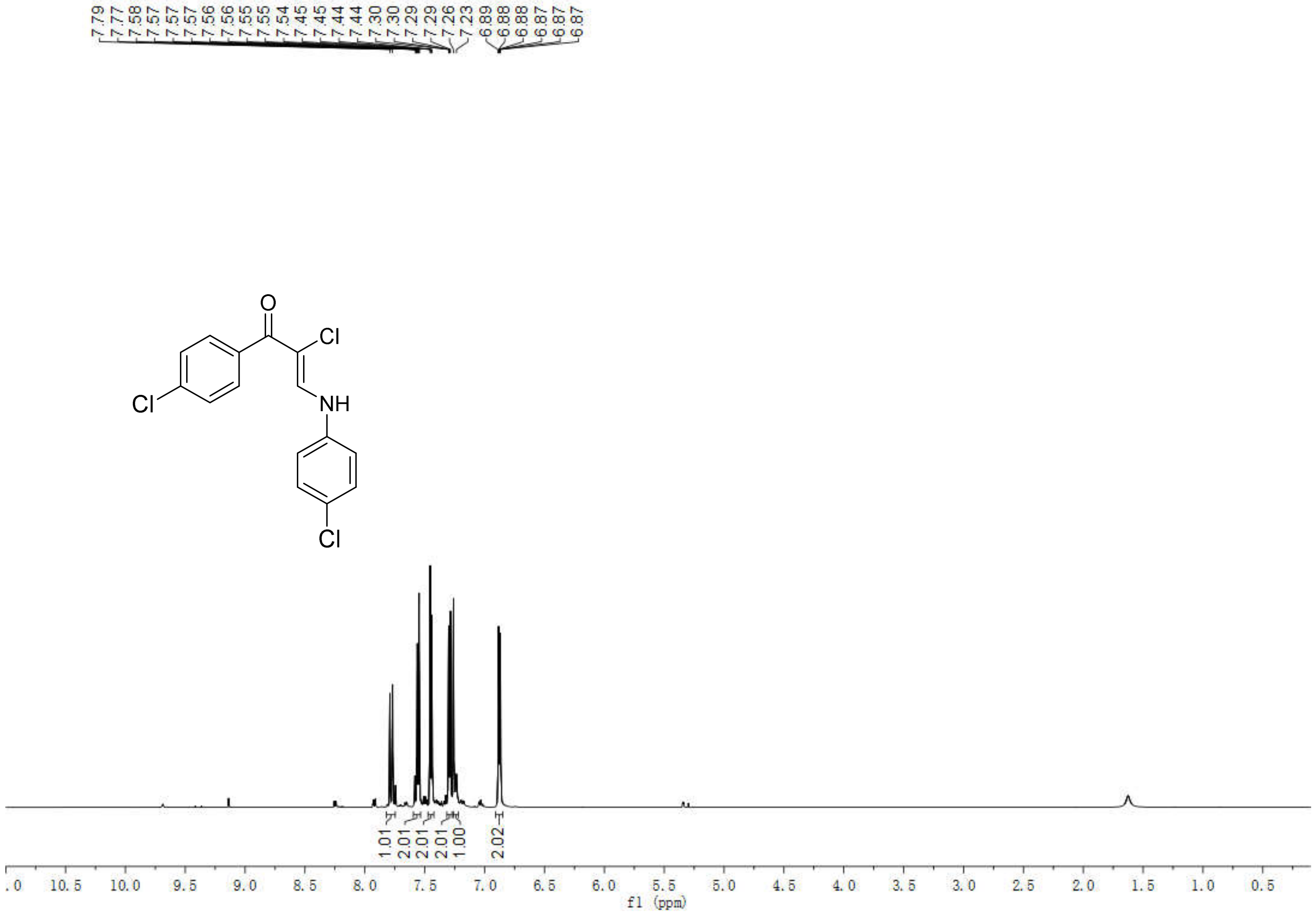

Figure S42. ^{1}H NMR (600 MHz, $CDCl_3$) spectra of compound **2n**

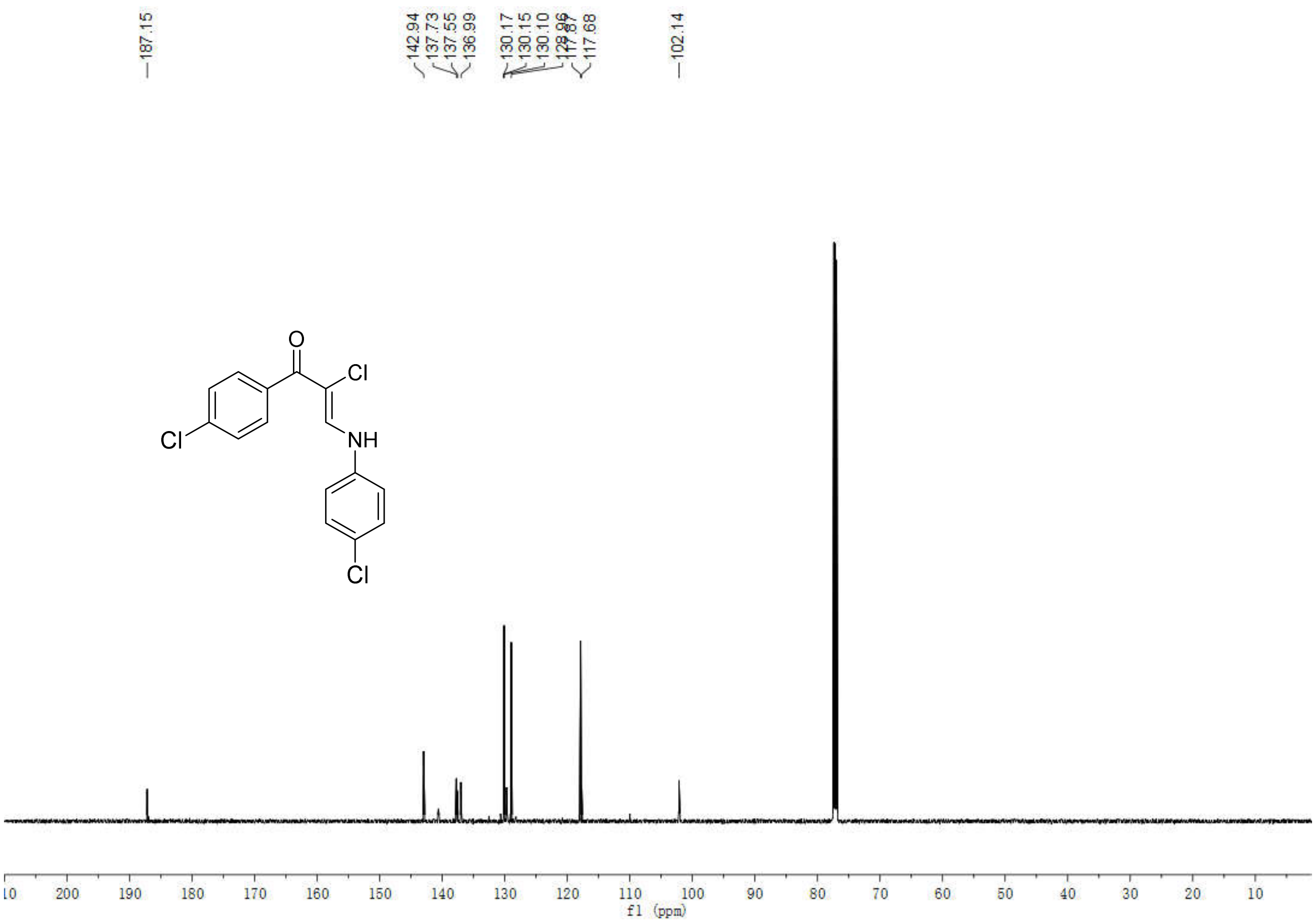

Figure S43. ^{13}C NMR (600 MHz, $CDCl_3$) spectra of compound **2n**

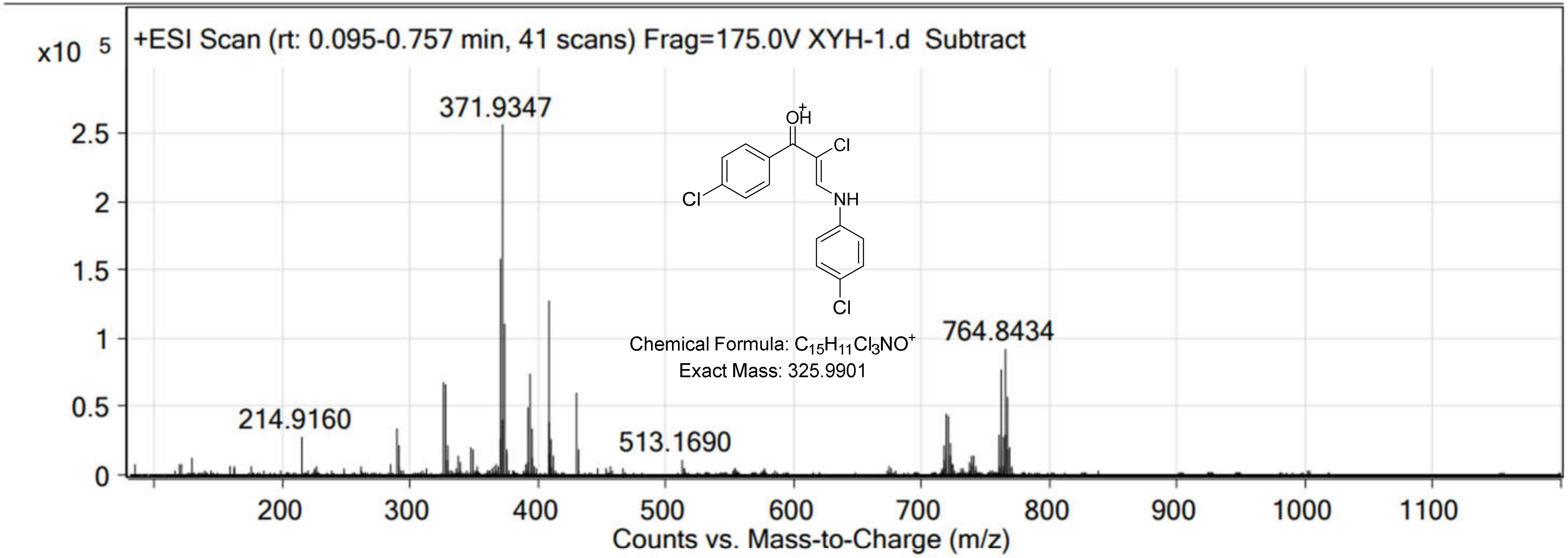

Peak List

m/z	z	Abund
290.0113	1	33456.68
325.9878	1	67744.57

Figure S44. HRMS spectra of compound **2m**

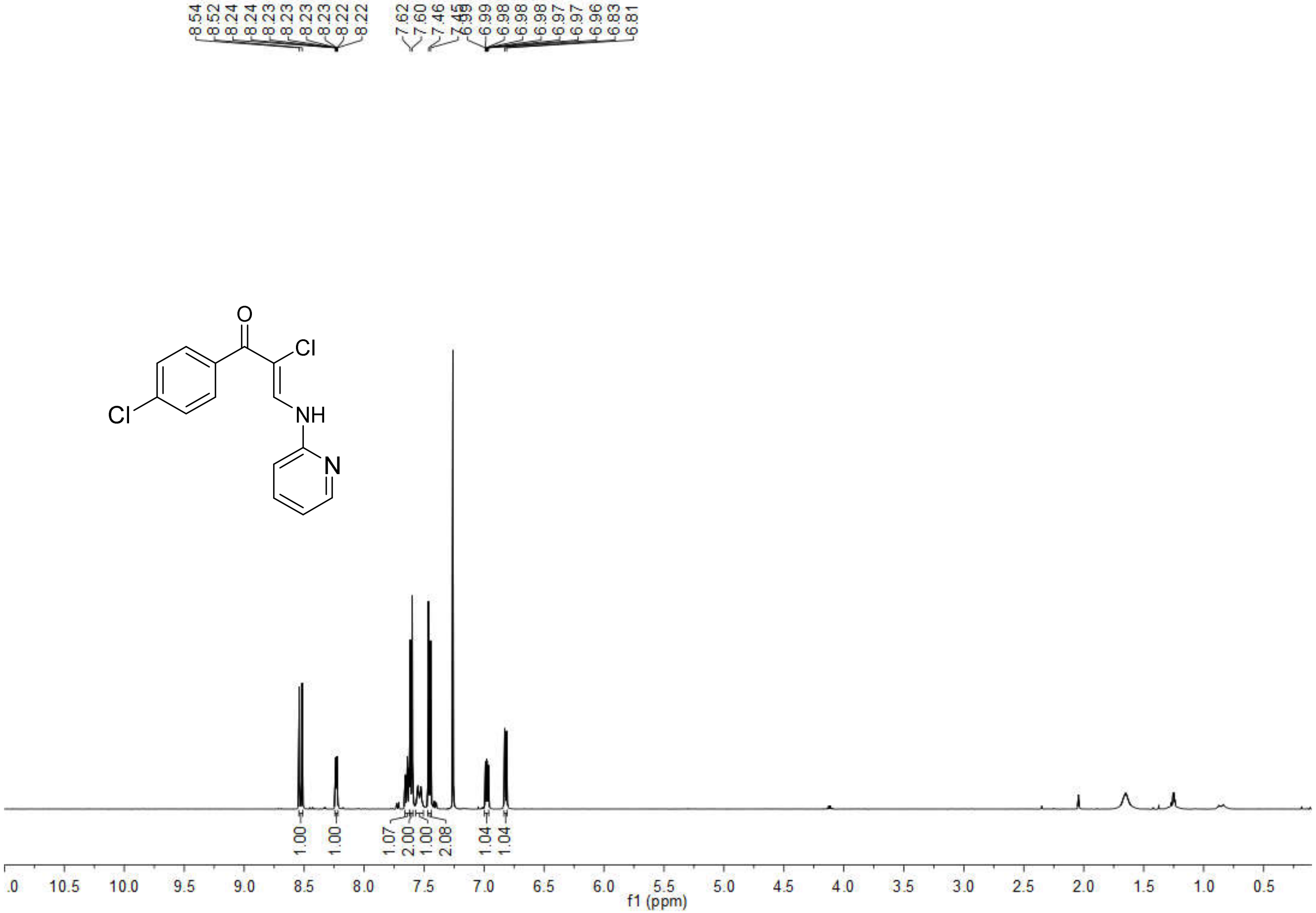

Figure S45. ^{1}H NMR (500 MHz, $CDCl_3$) spectra of compound **2o**

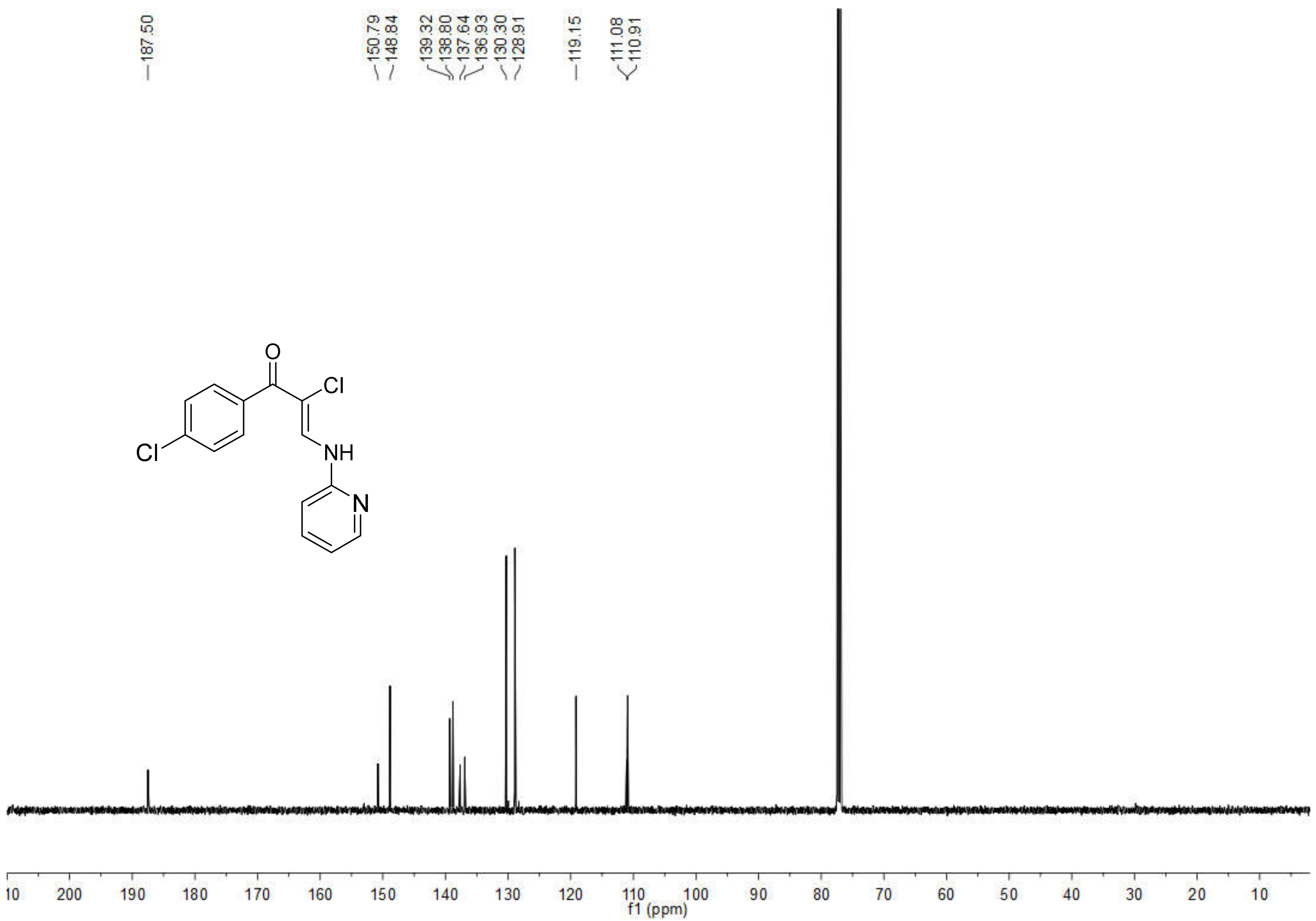

Figure S46. ^{13}C NMR (500 MHz, $CDCl_3$) spectra of compound **2o**

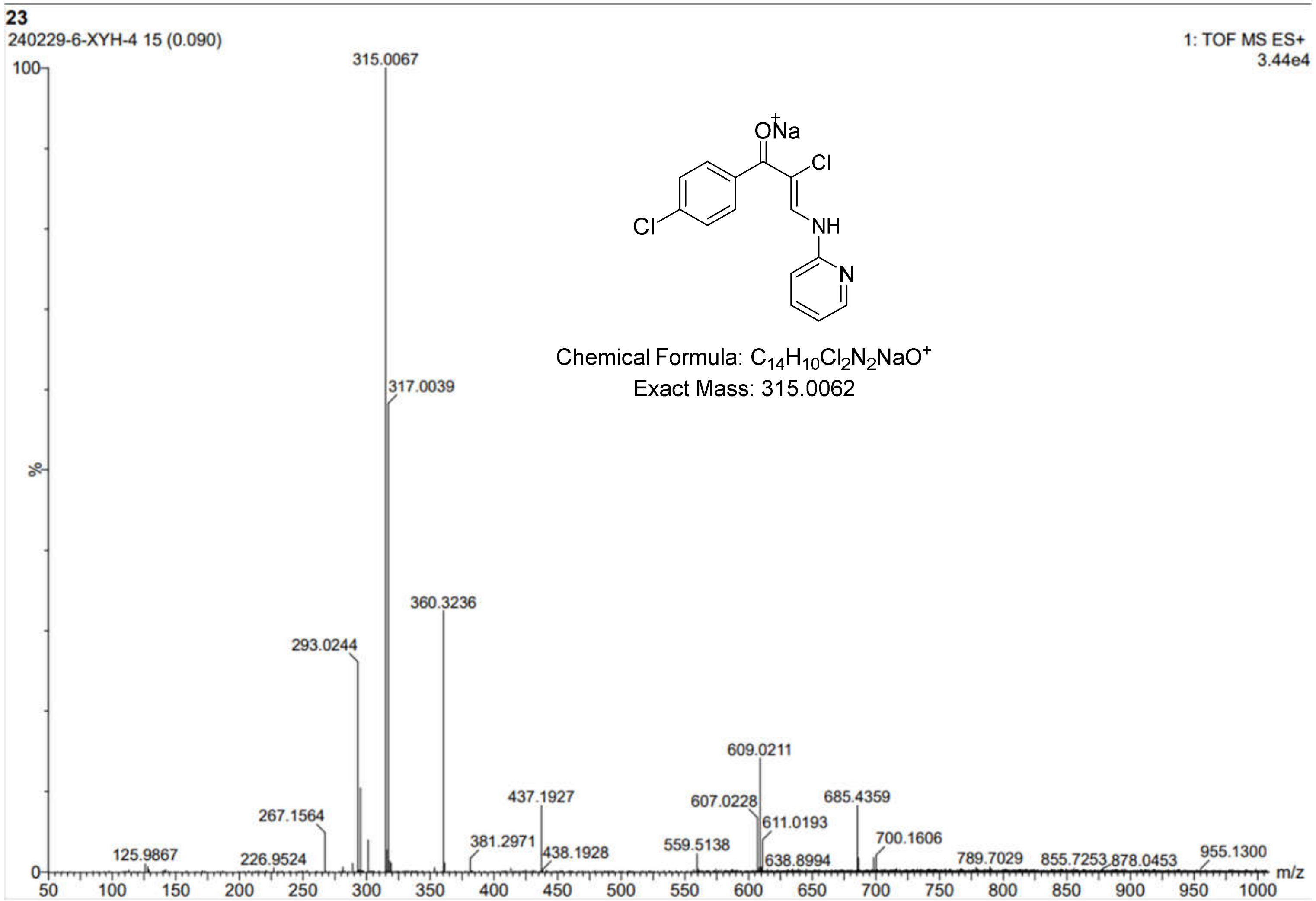

Figure S47. HRMS spectra of compound **2o**

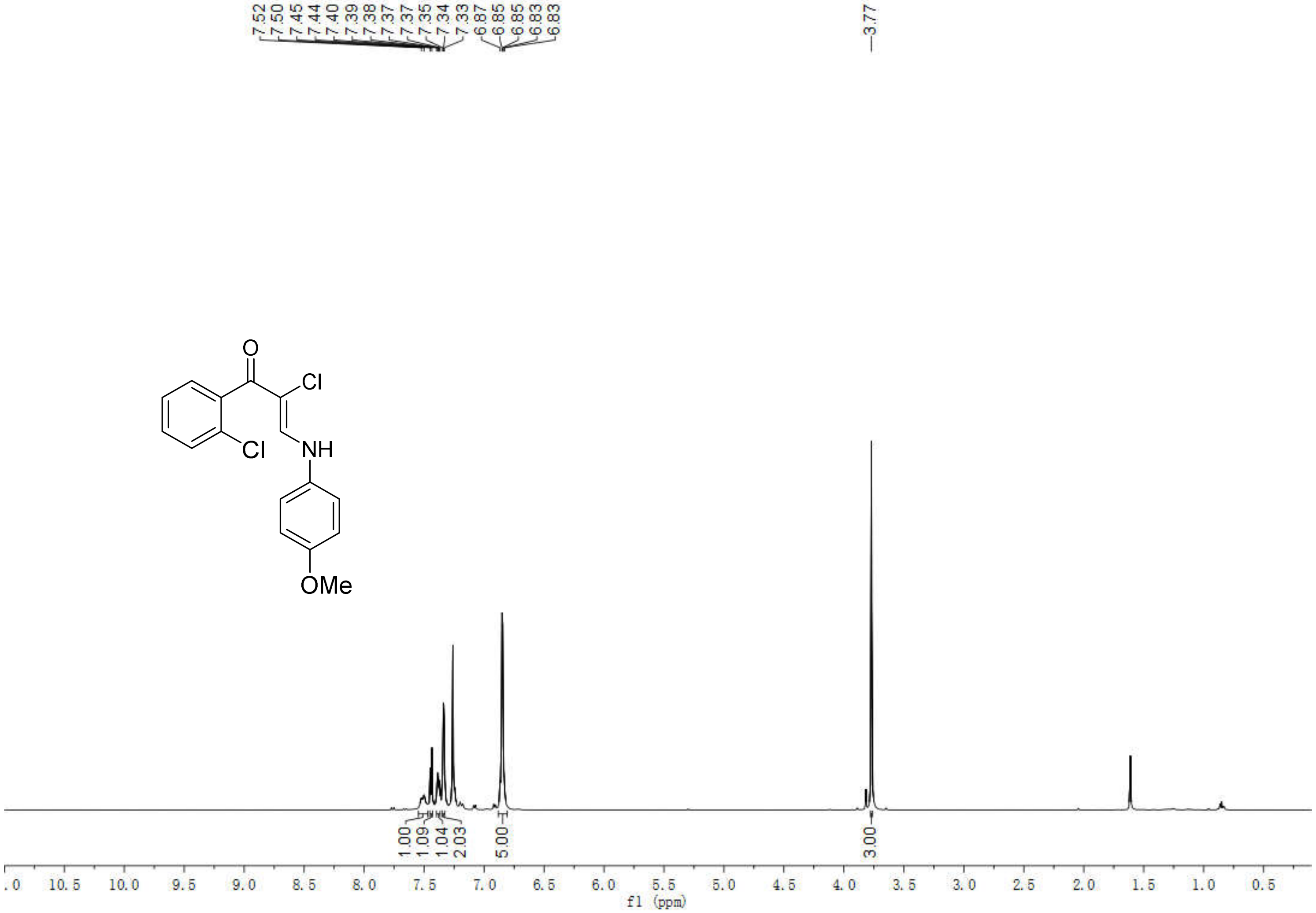

Figure S48. ^{1}H NMR (600 MHz, $CDCl_3$) spectra of compound **2p**

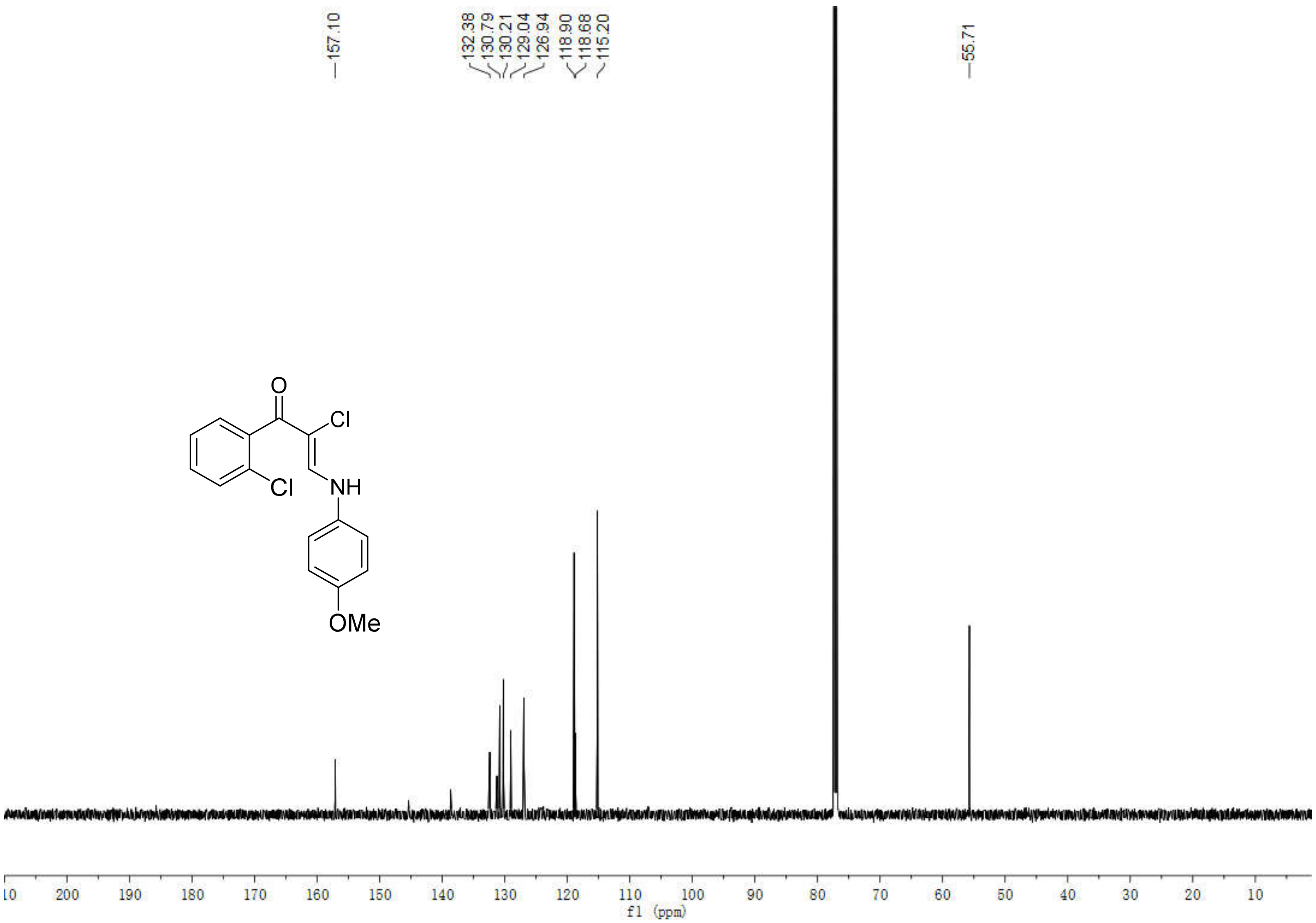

Figure S49. ^{13}C NMR (600 MHz, $CDCl_3$) spectra of compound **2p**

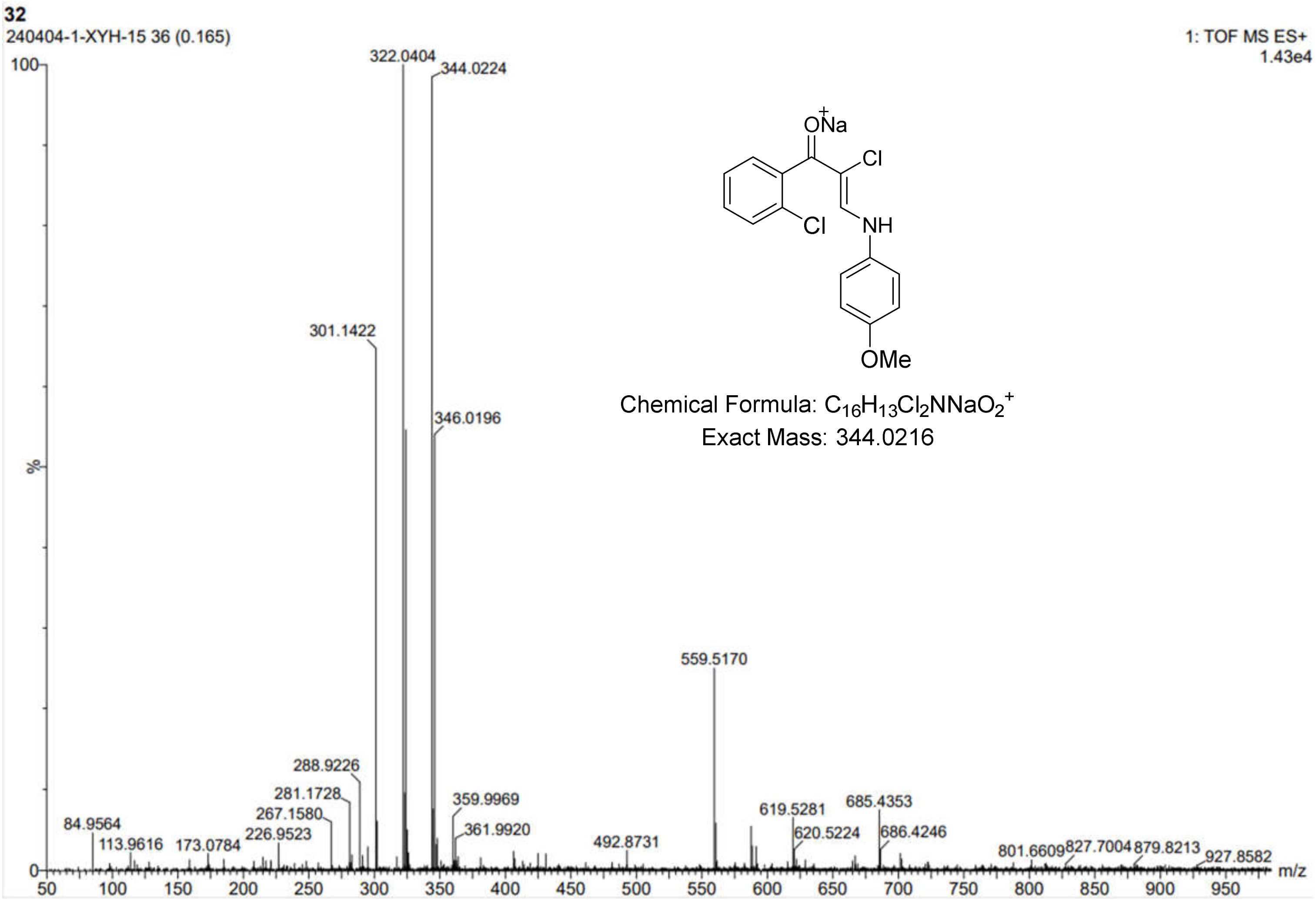

Figure S50. HRMS spectra of compound **2p**

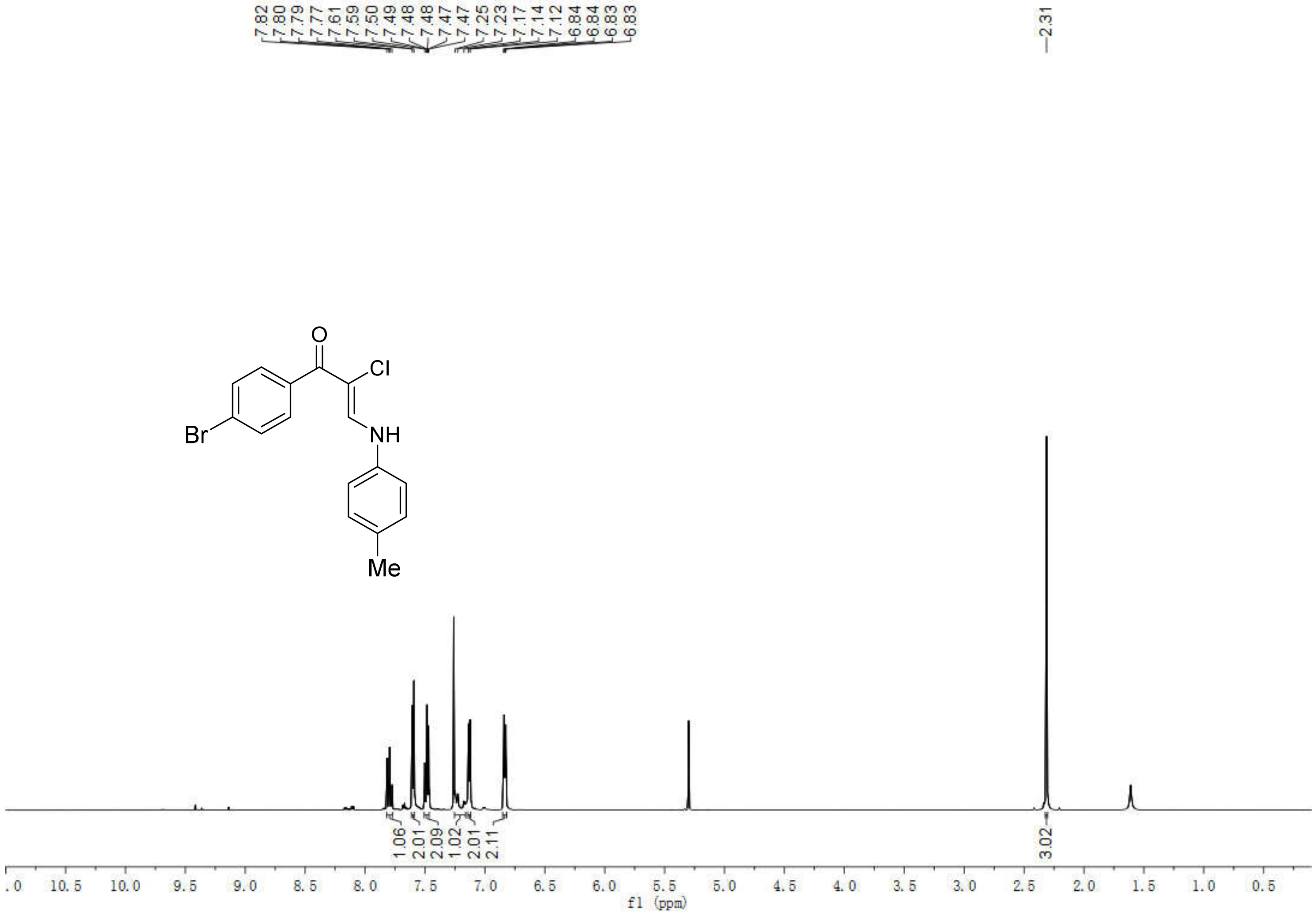

Figure S51. ^{1}H NMR (600 MHz, $CDCl_3$) spectra of compound **2q**

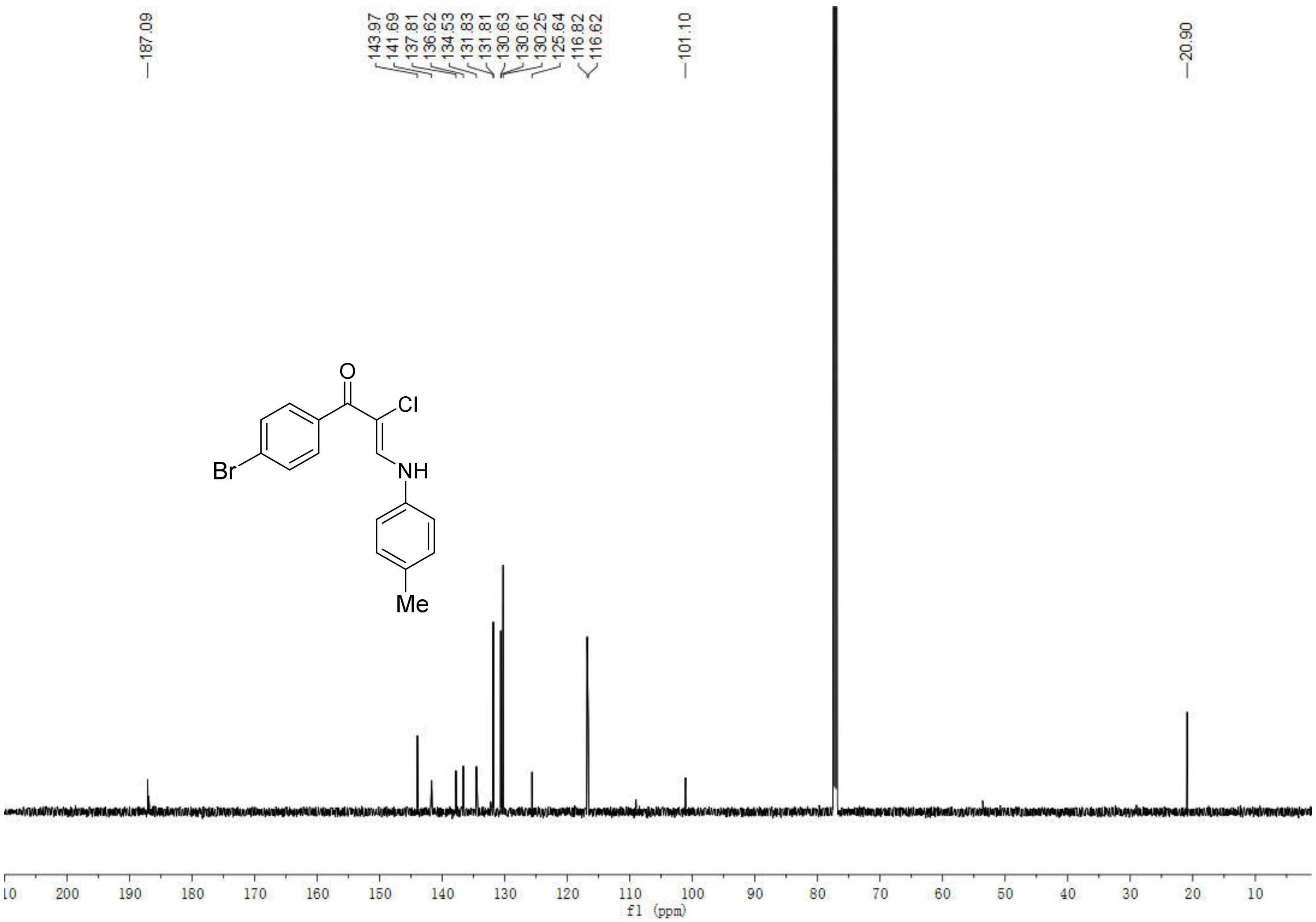

Figure S52. ^{13}C NMR (600 MHz, $CDCl_3$) spectra of compound **2q**

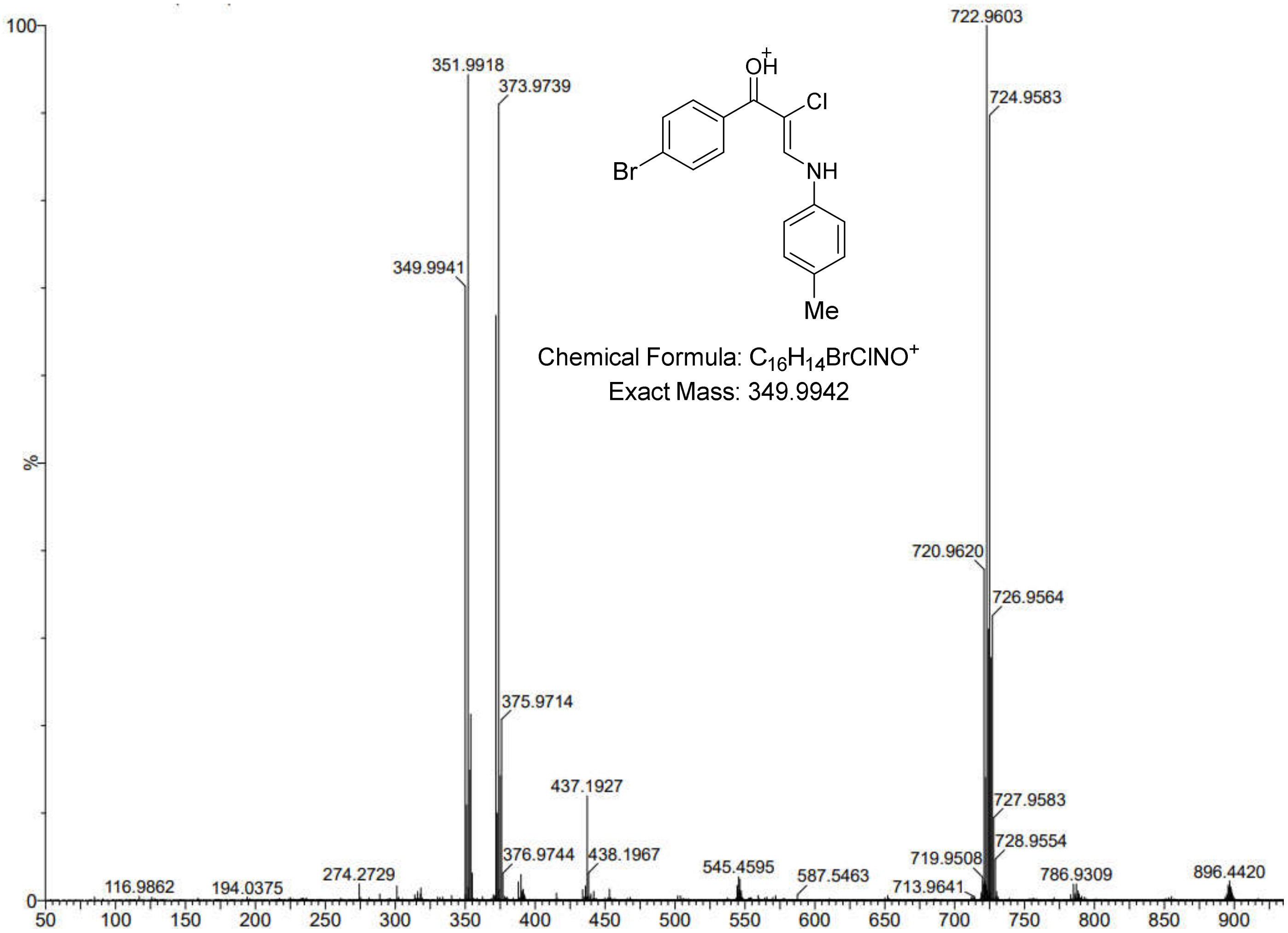

Figure S53. HRMS spectra of compound **2q**

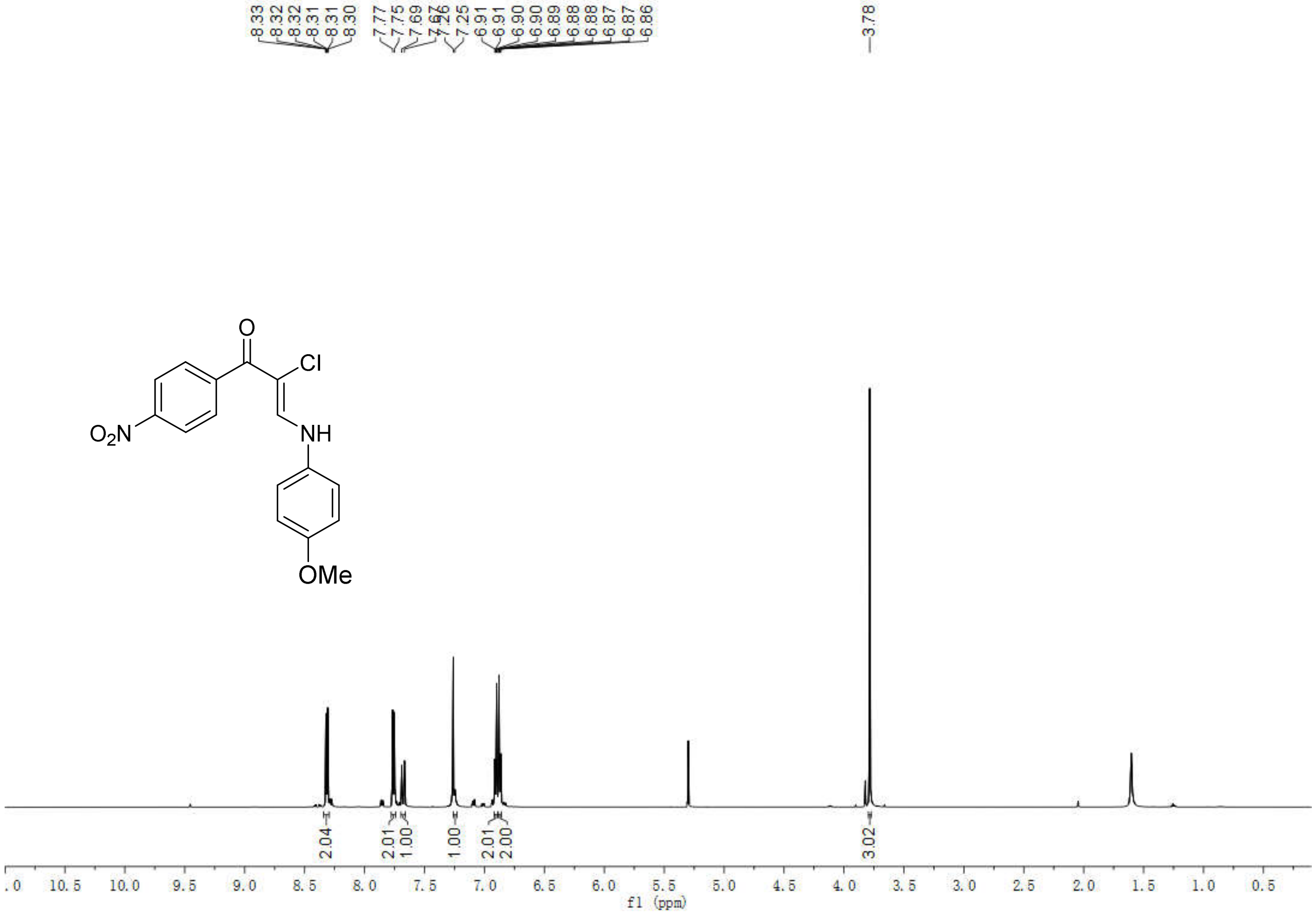

Figure S54. ^{1}H NMR (600 MHz, $CDCl_3$) spectra of compound **2r**

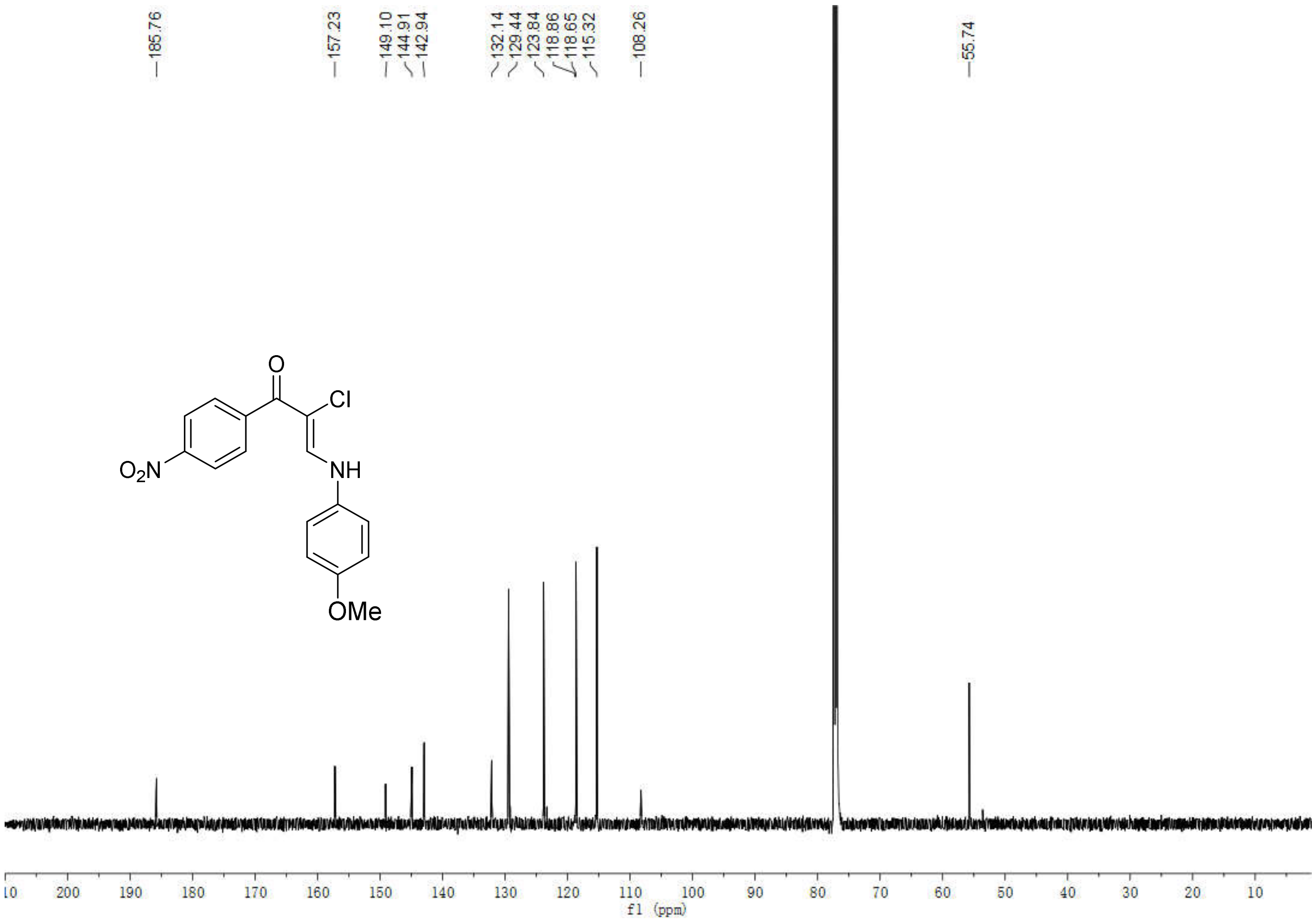

Figure S55. ^{13}C NMR (600 MHz, $CDCl_3$) spectra of compound **2r**

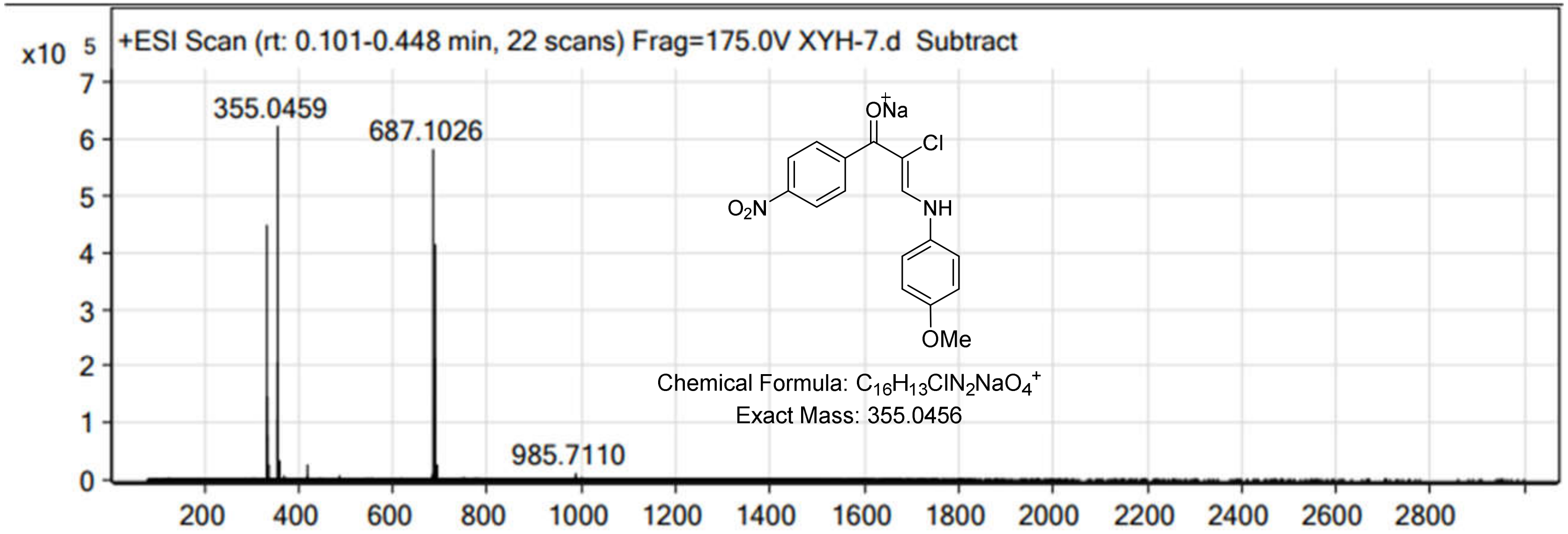

Figure S56. HRMS spectra of compound **2r**

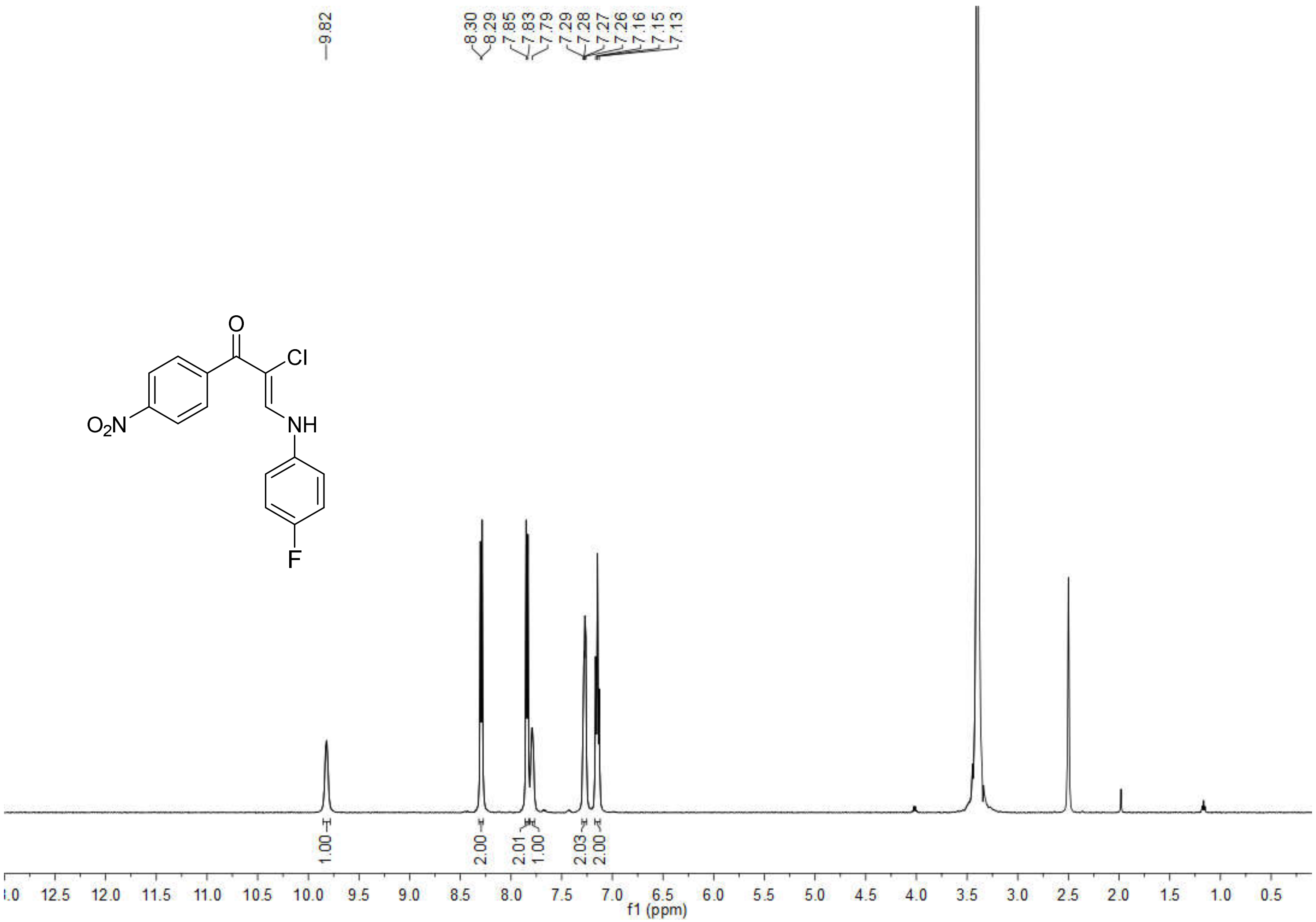

Figure S57. ^{1}H NMR (500 MHz, DMSO-d_6) spectra of compound **2s**

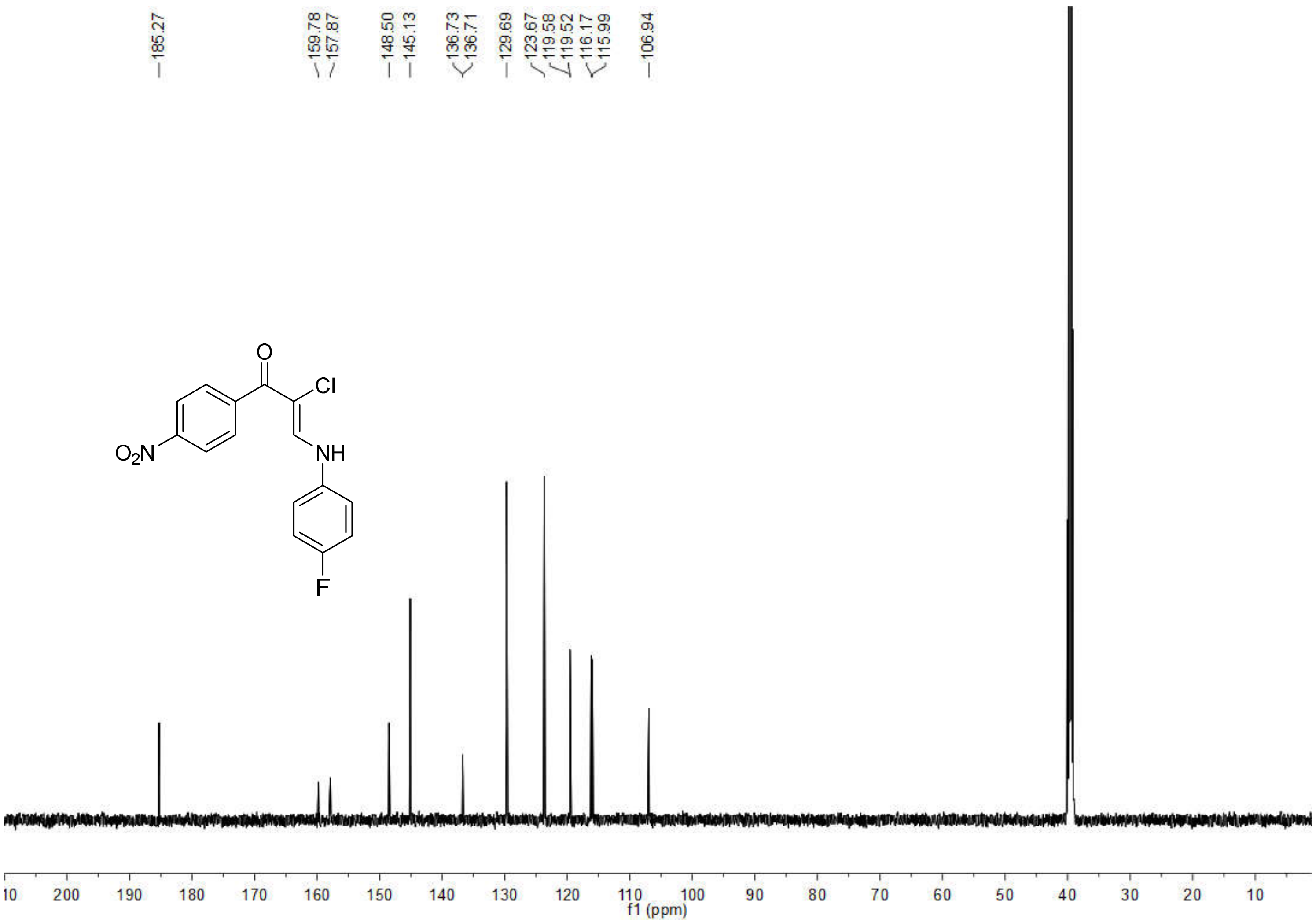

Figure S58. ^{13}C NMR (500 MHz, DMSO-d_6) spectra of compound **2s**

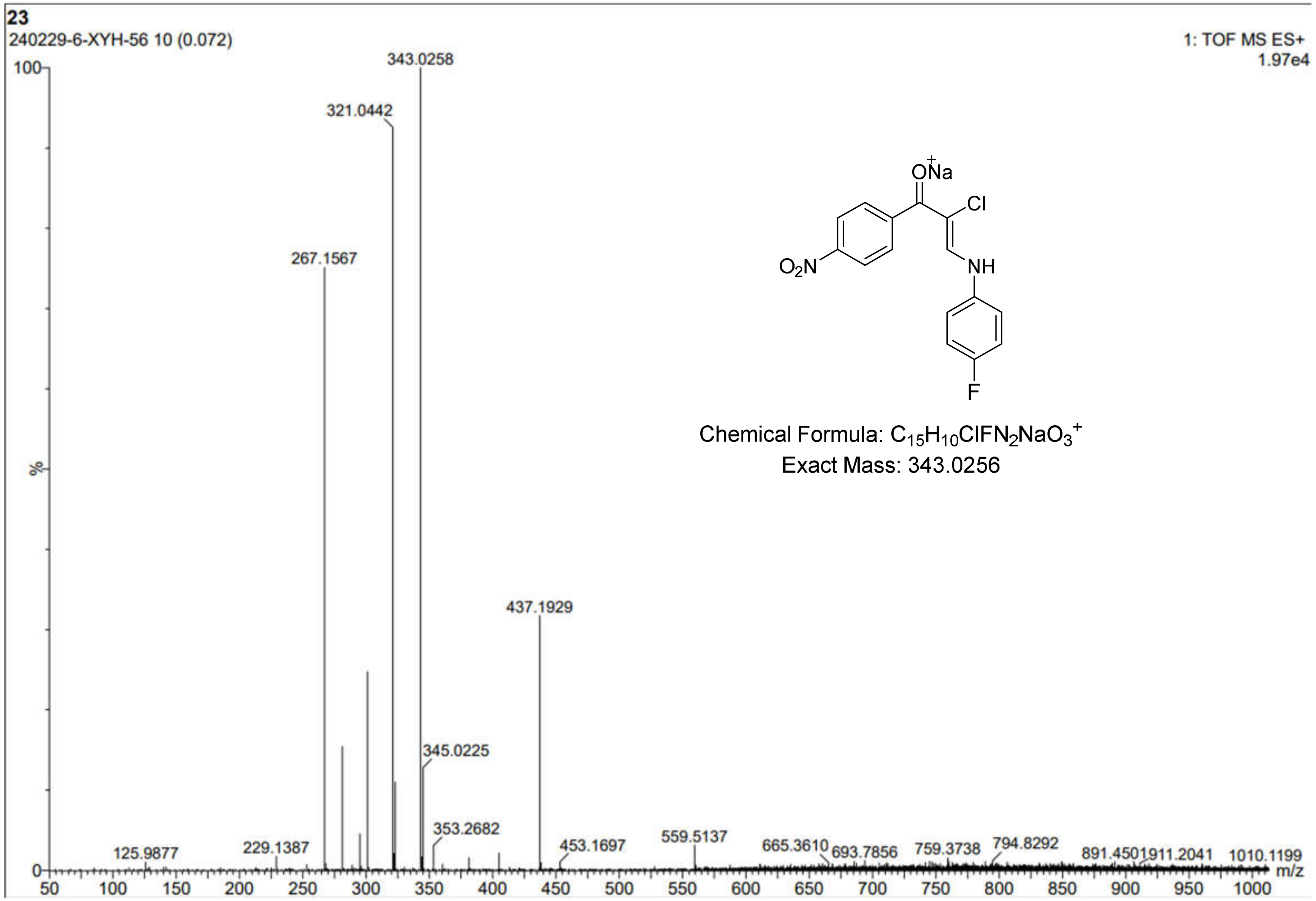

Figure S59. HRMS spectra of compound **2s**

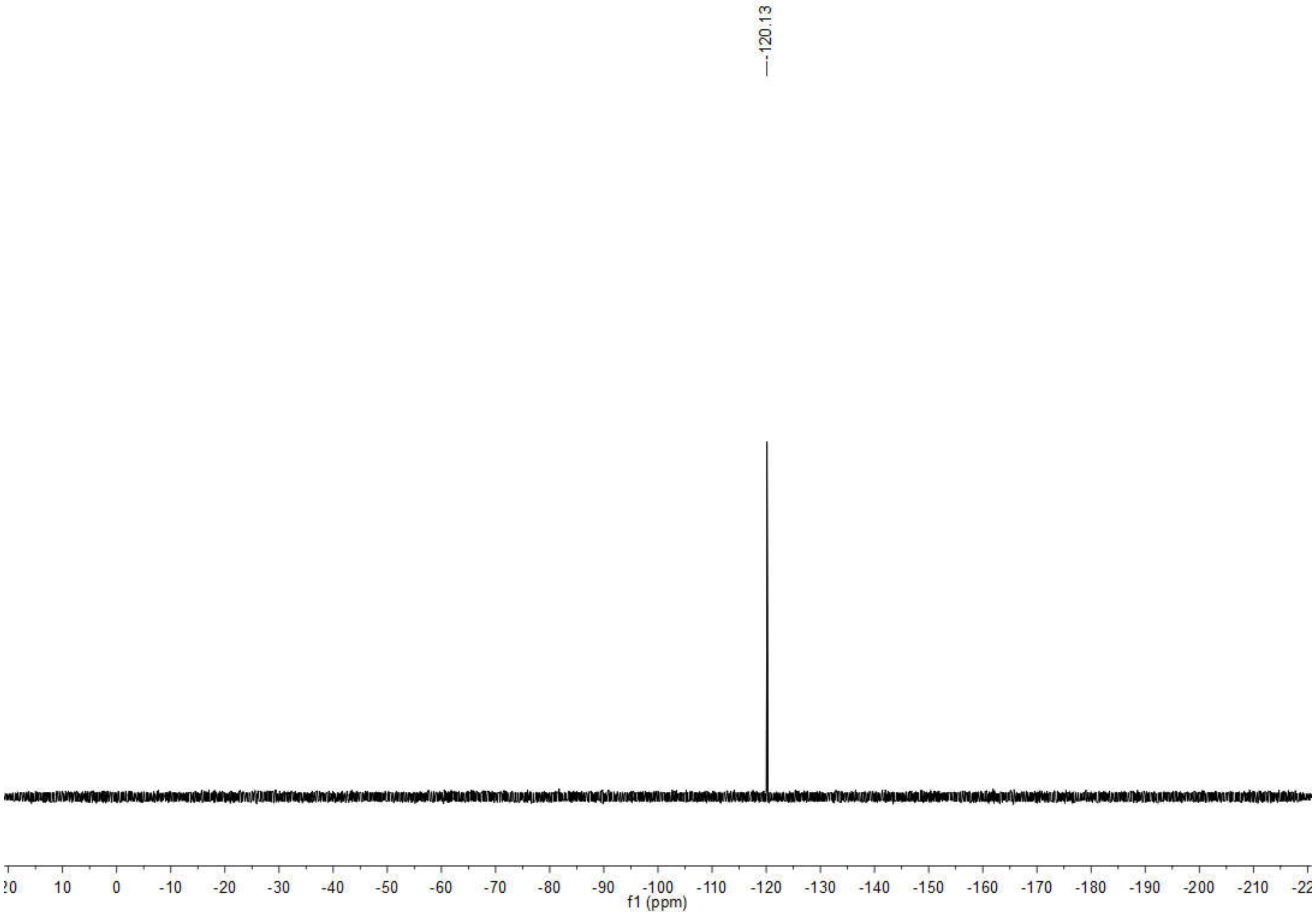

Figure S60. ^{19}F NMR (500 MHz, DMSO-d_6) spectra of compound **2s**

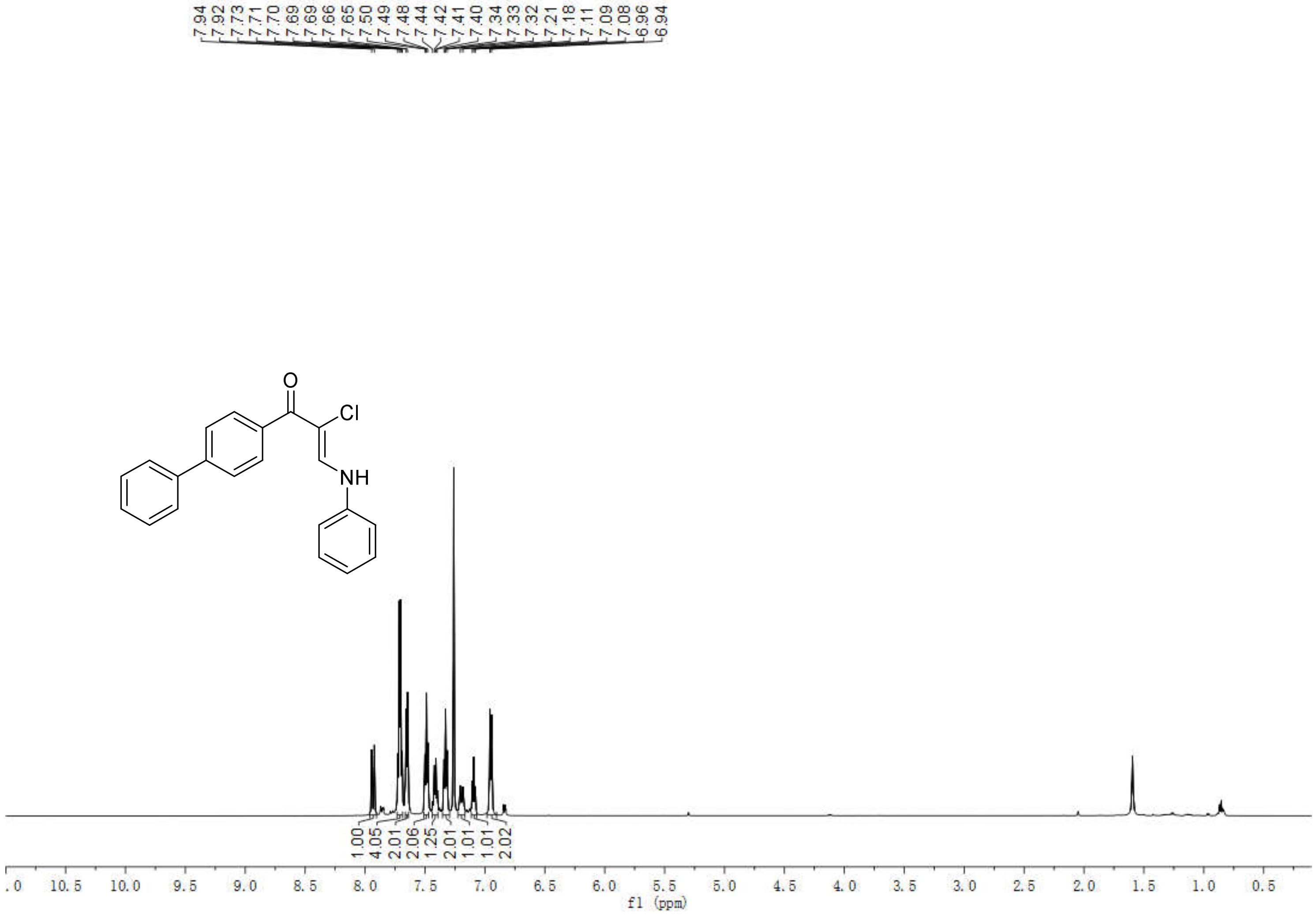

Figure S61. ^{1}H NMR (600 MHz, $CDCl_3$) spectra of compound **2t**

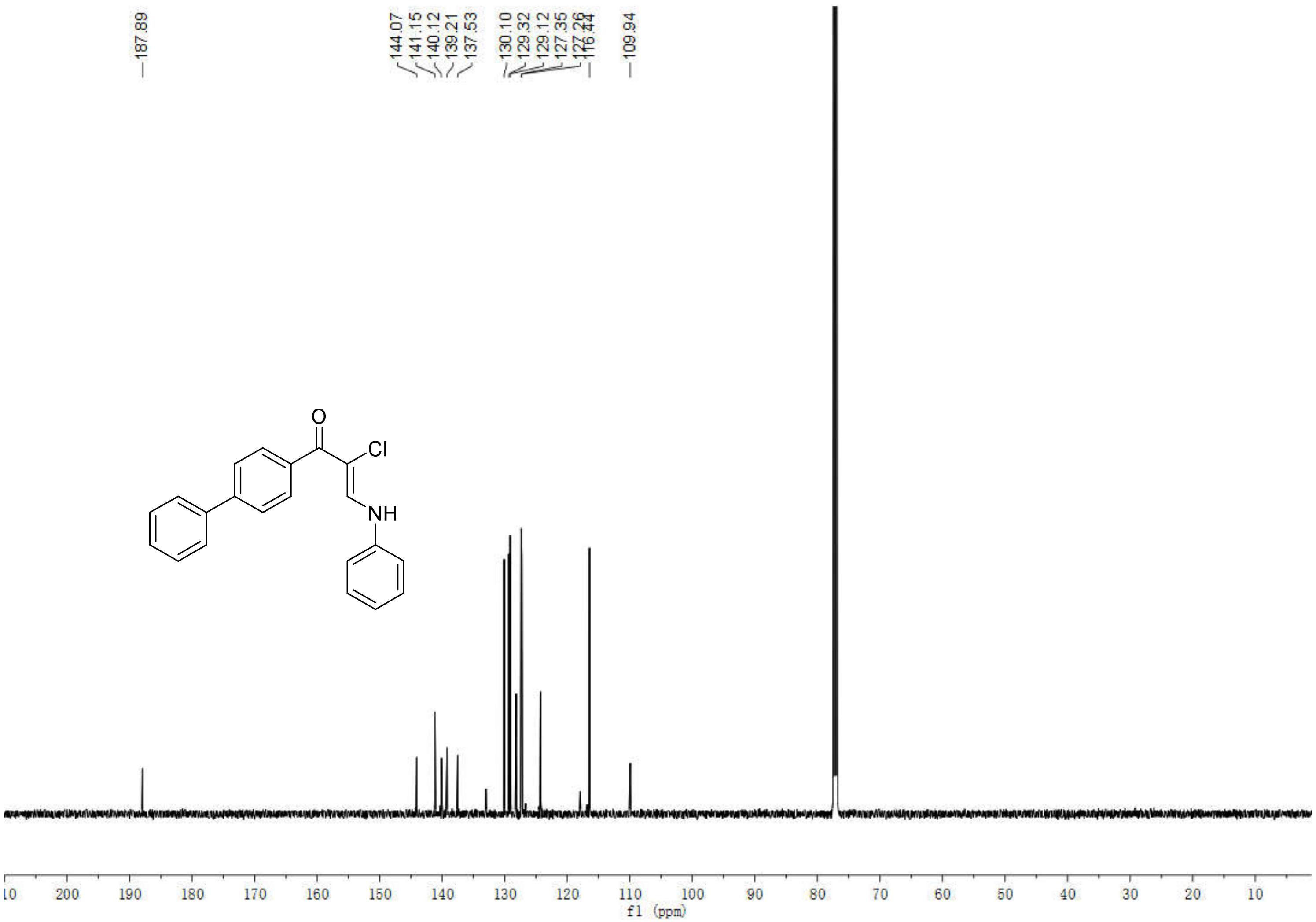

Figure S62. ^{13}C NMR (600 MHz, $CDCl_3$) spectra of compound **2t**

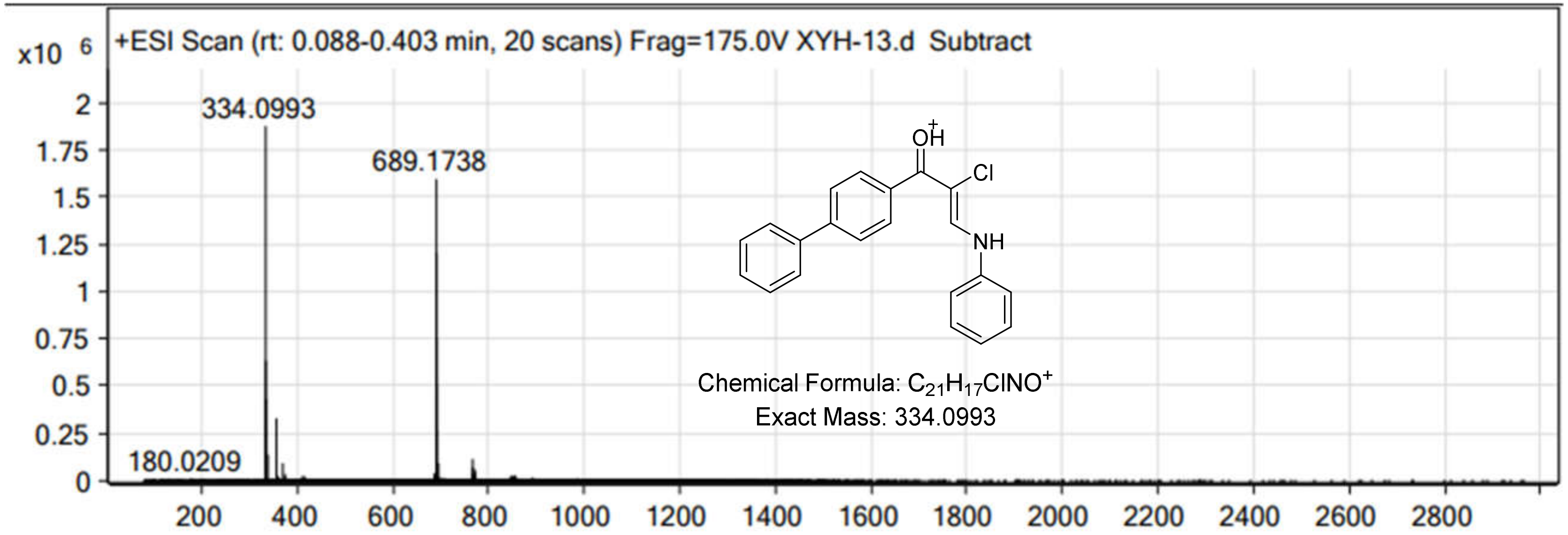

Figure S63. HRMS spectra of compound **2t**

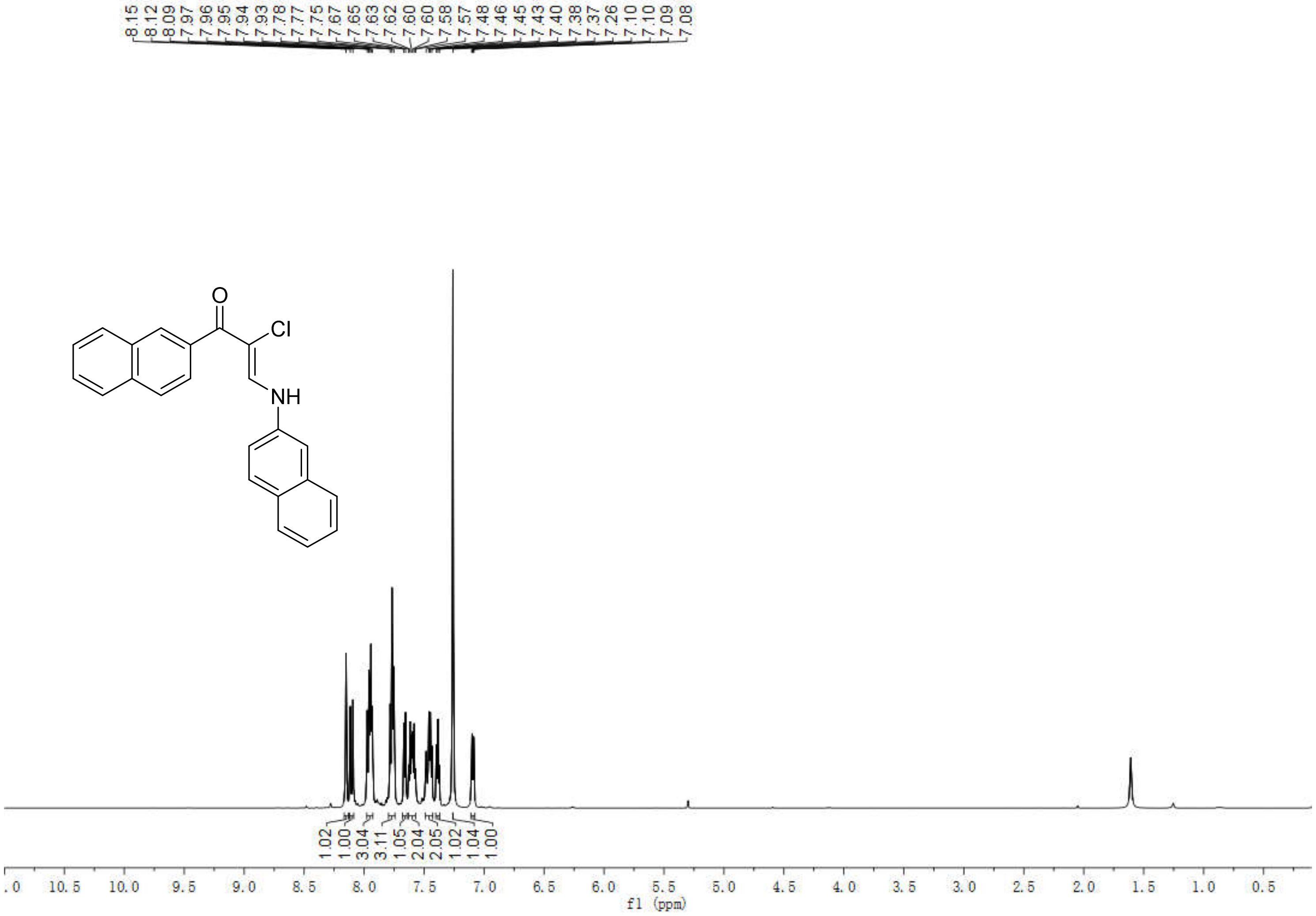

Figure S64. ^{1}H NMR (600 MHz, $CDCl_3$) spectra of compound **2u**

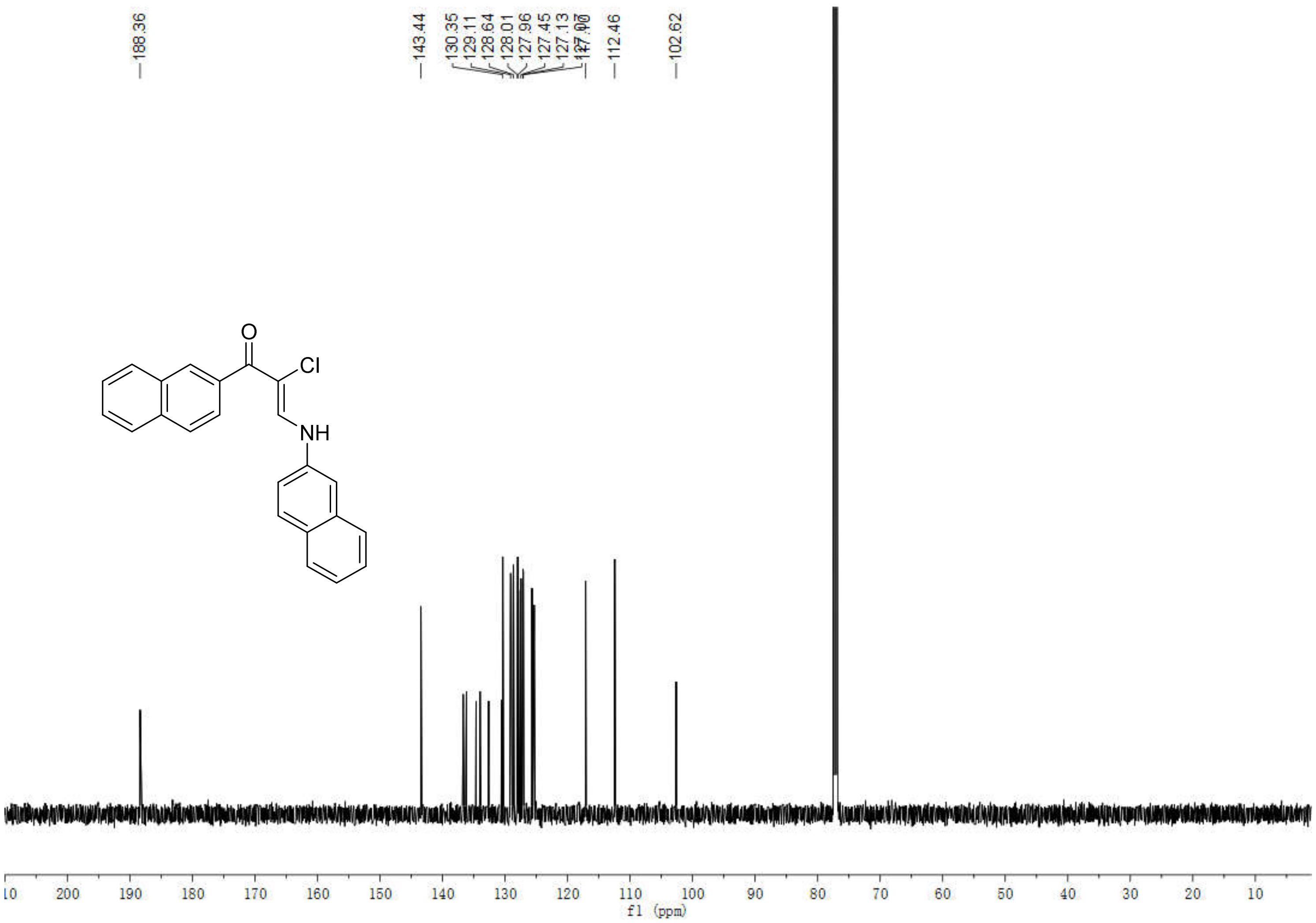

Figure S65 ^{13}C NMR (600 MHz, $CDCl_3$) spectra of compound **2u**

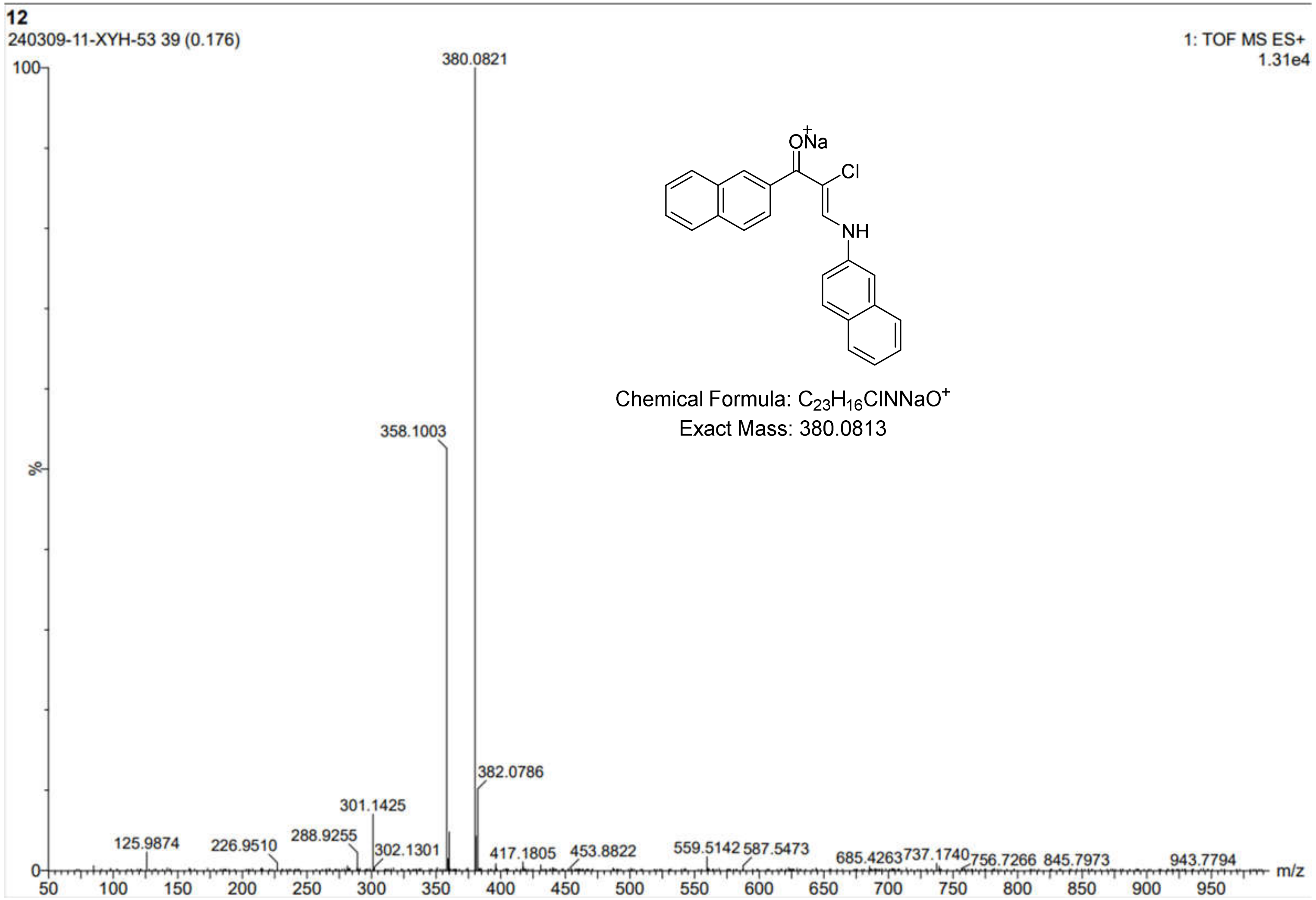

Figure S66. HRMS spectra of compound **2u**

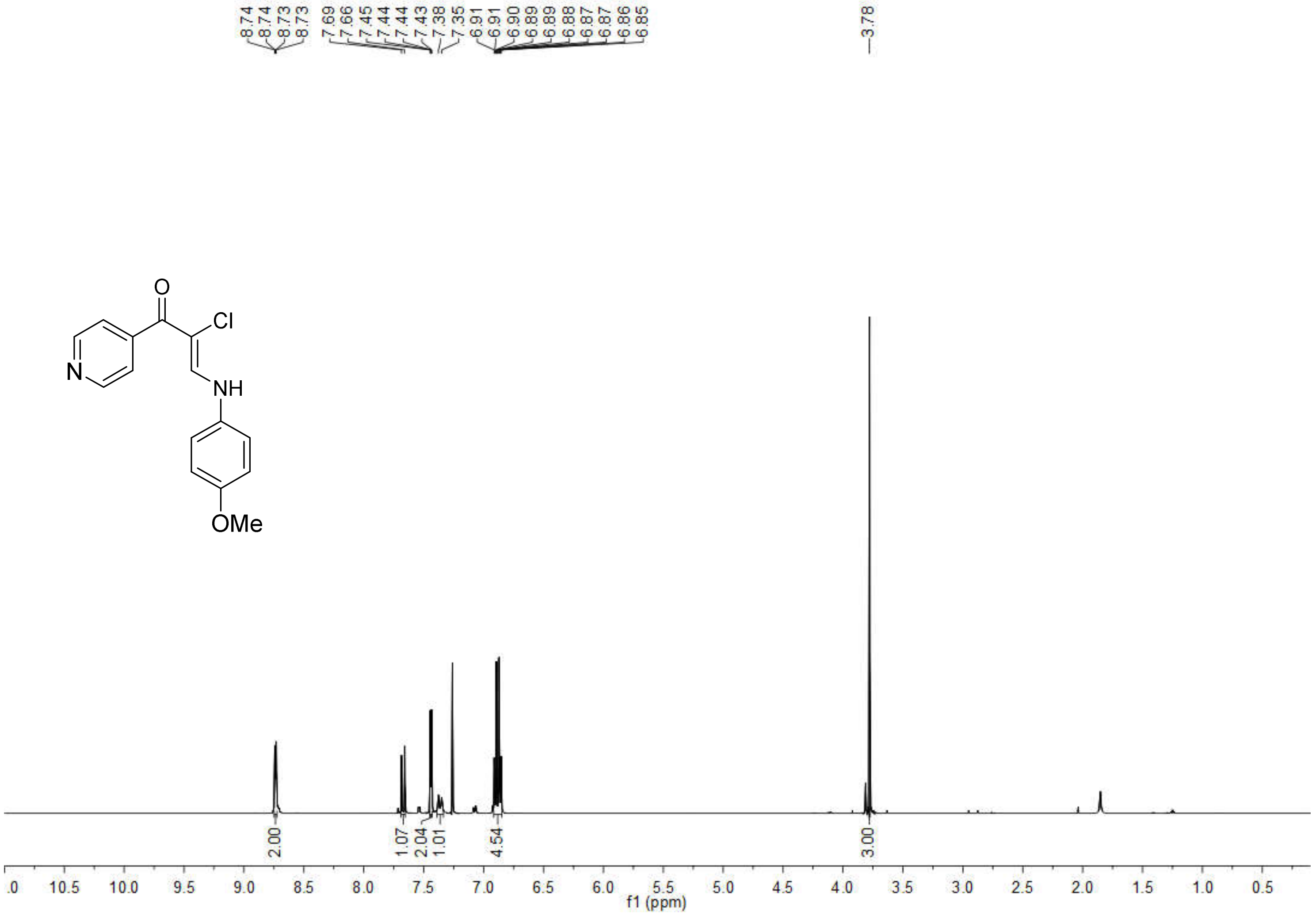

Figure S67. ^{1}H NMR (500 MHz, $CDCl_3$) spectra of compound **2v**

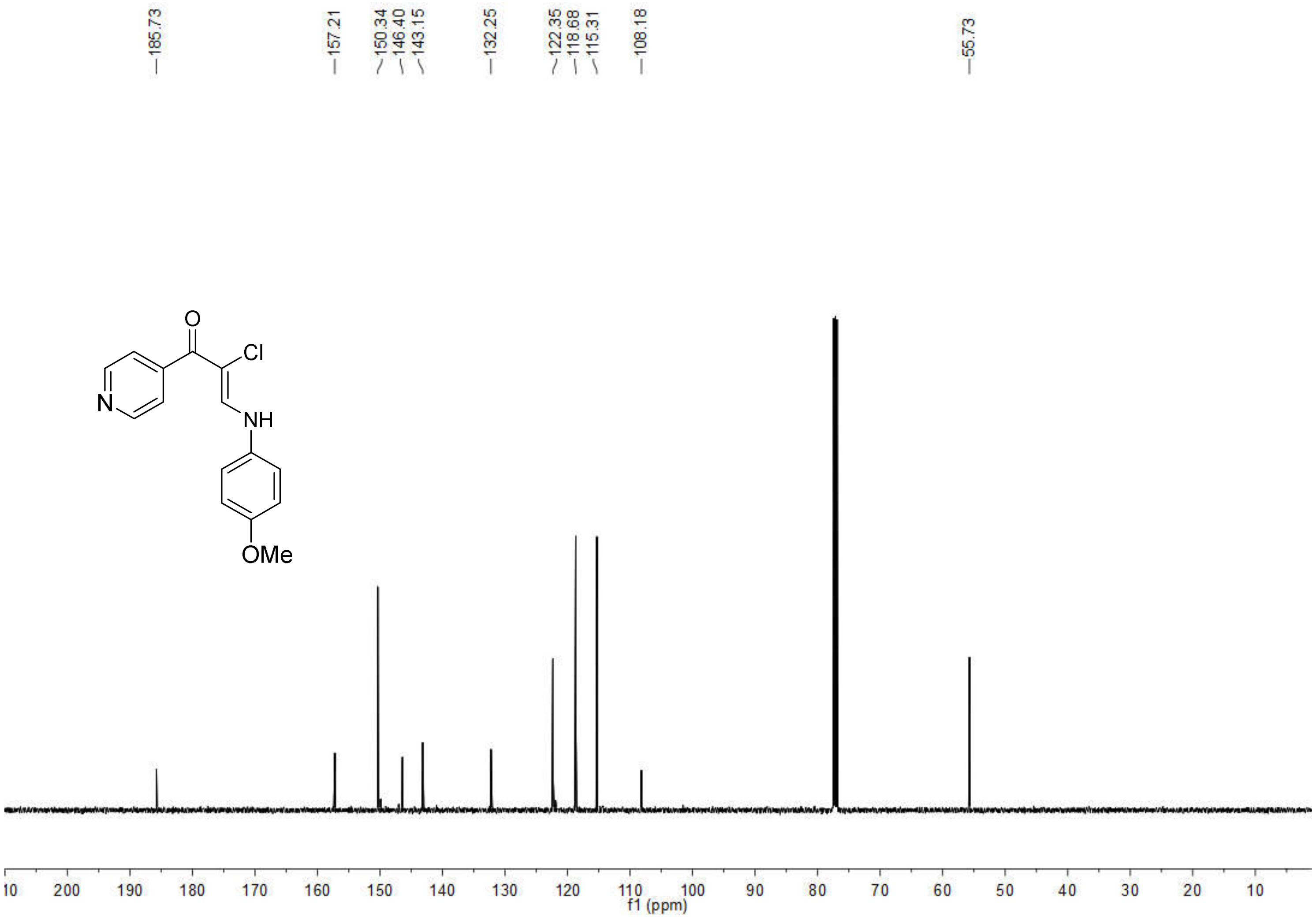

Figure S68. ^{13}C NMR (500 MHz, $CDCl_3$) spectra of compound **2v**

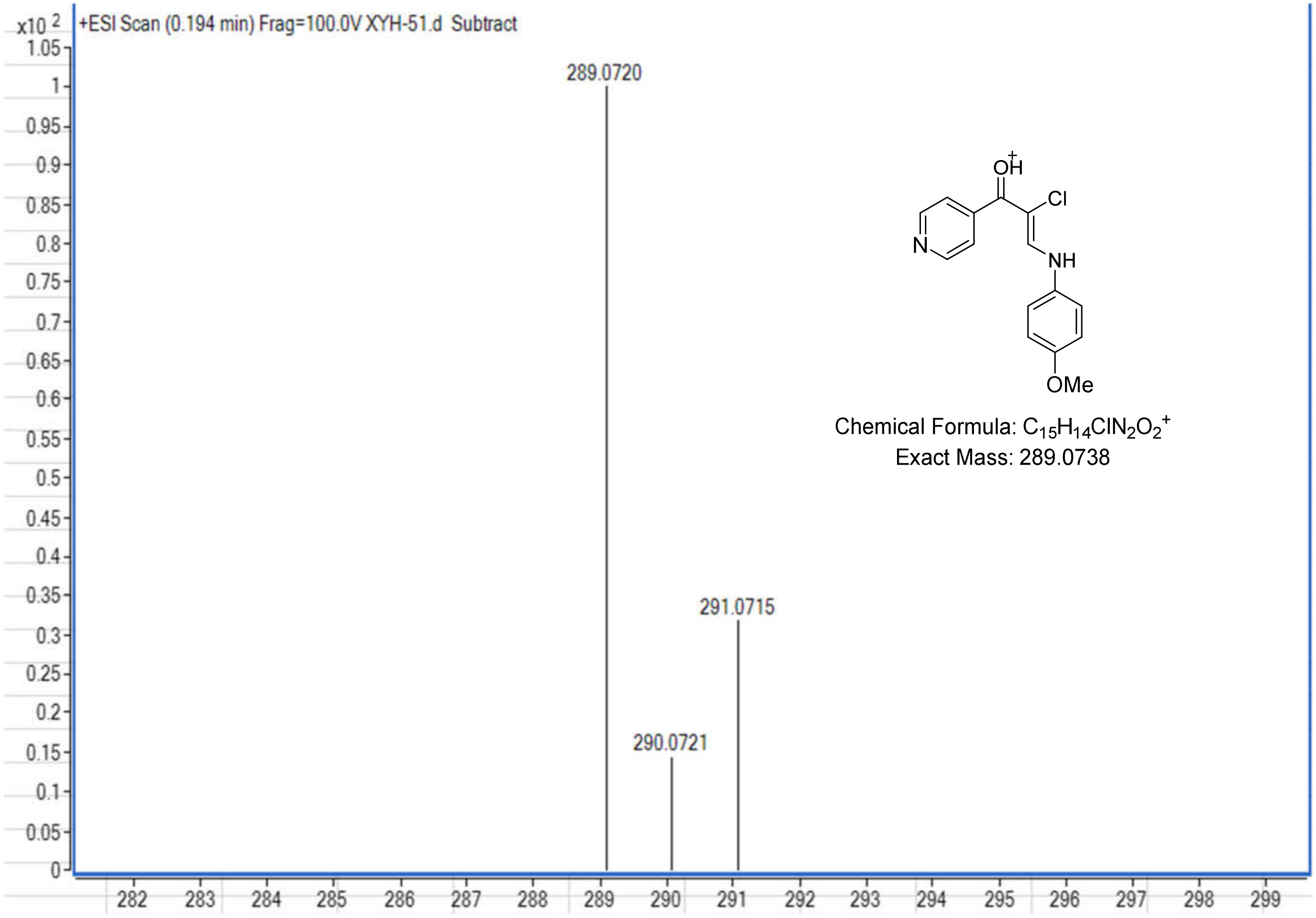

Figure S69. HRMS spectra of compound **2v**

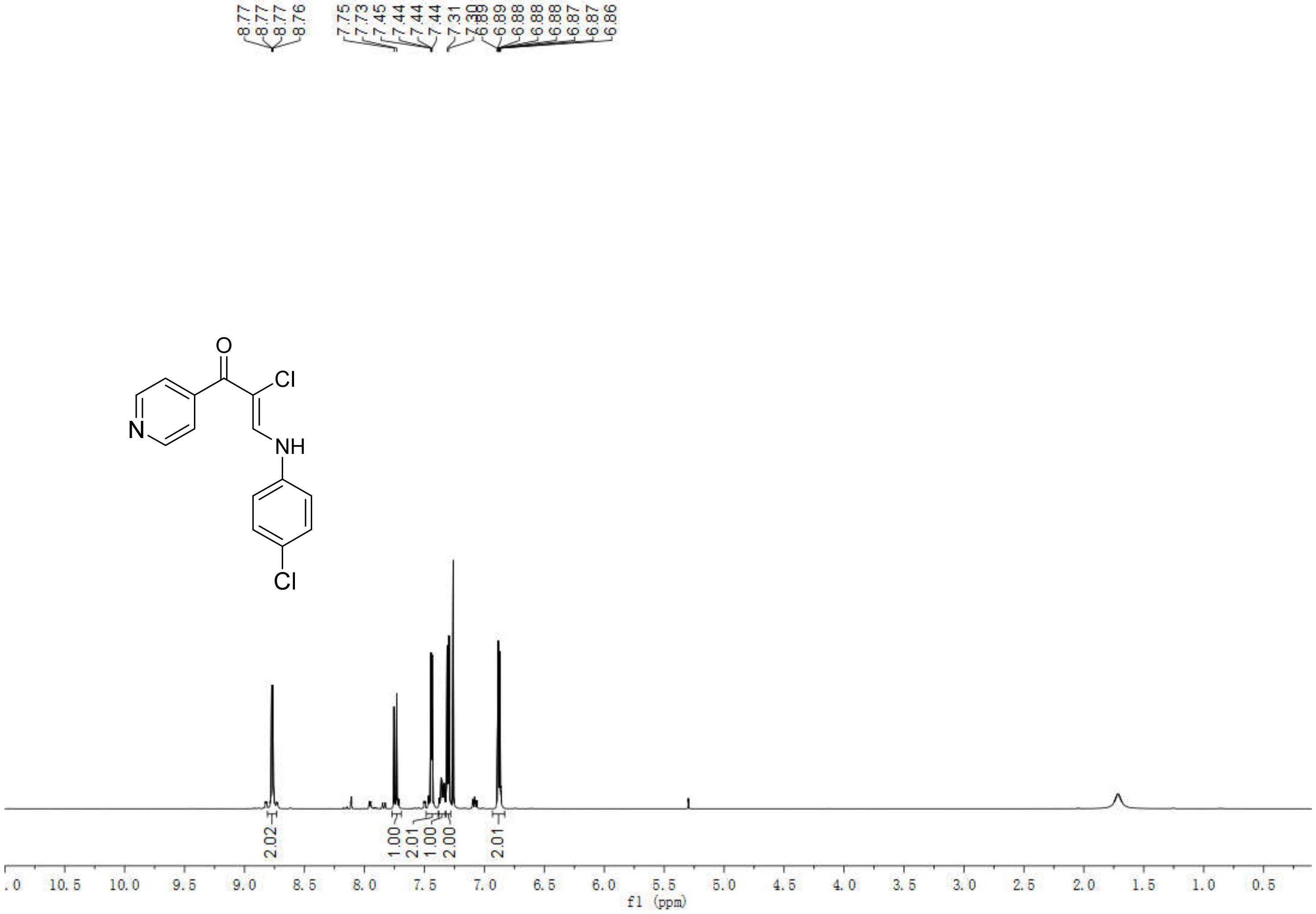

Figure S70. ¹H NMR (600 MHz, $CDCl_3$) spectra of compound **2w**

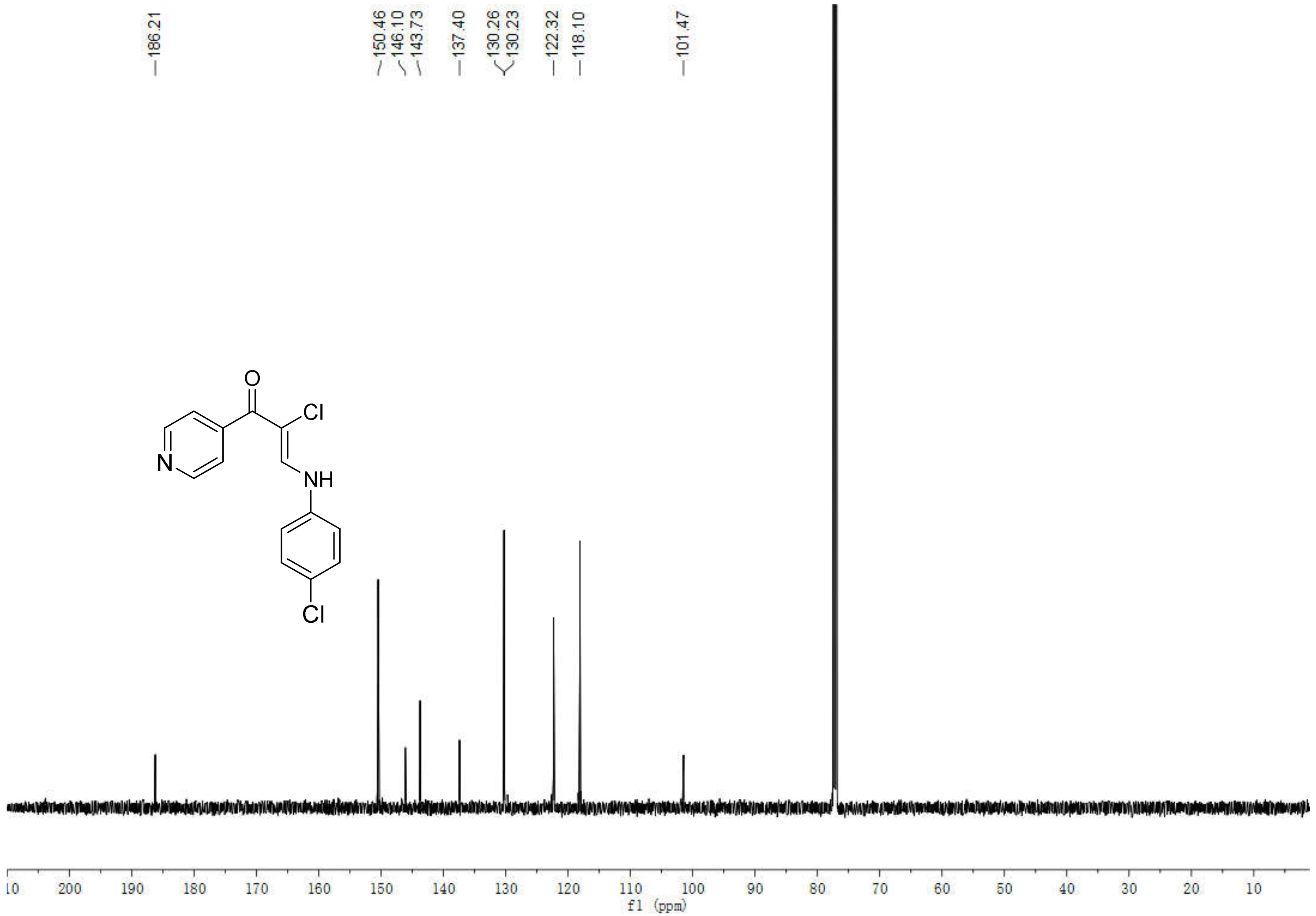

Figure S71. ^{13}C NMR (600 MHz, $CDCl_3$) spectra of compound **2w**

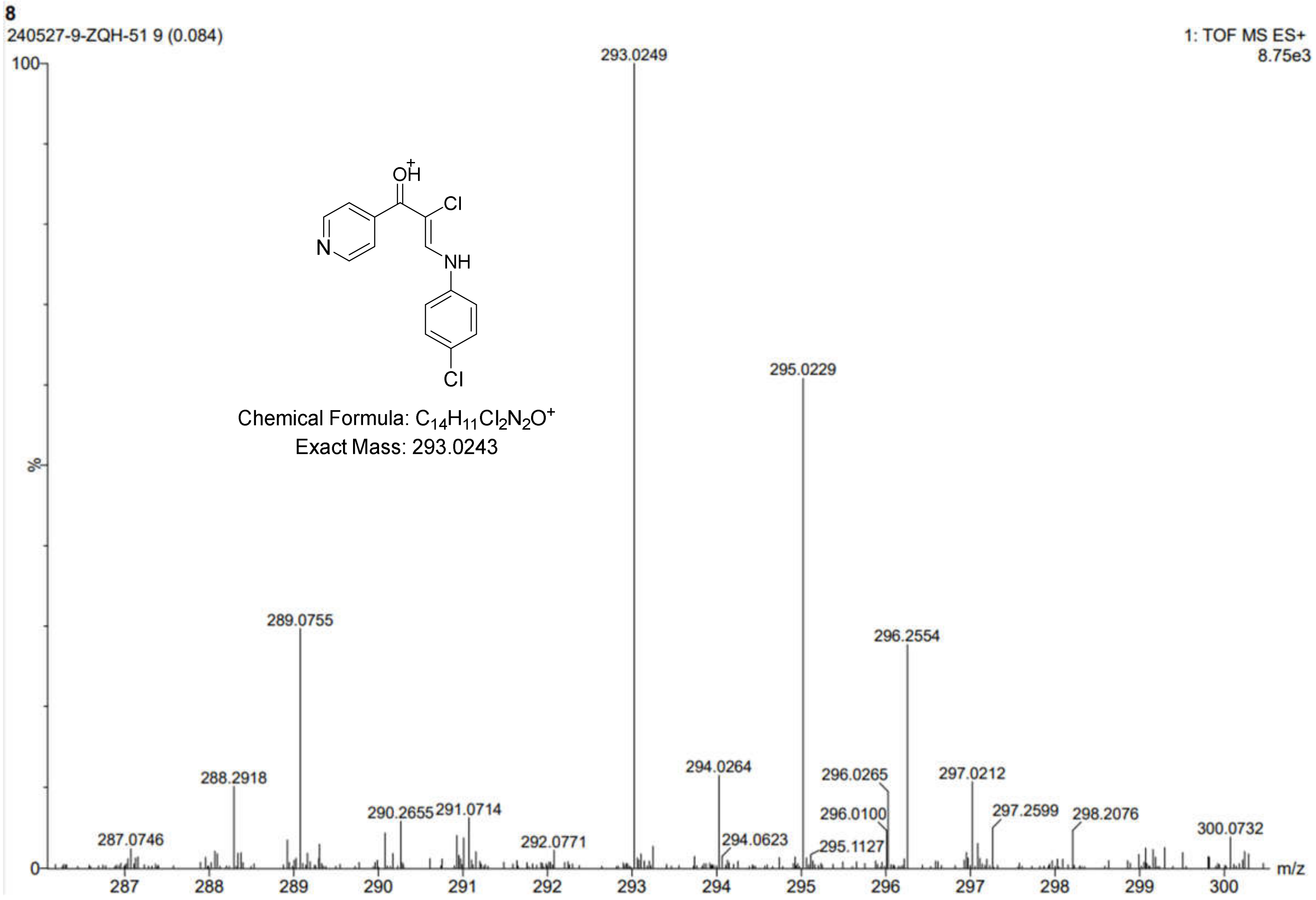

Figure S72. HRMS spectra of compound **2w**

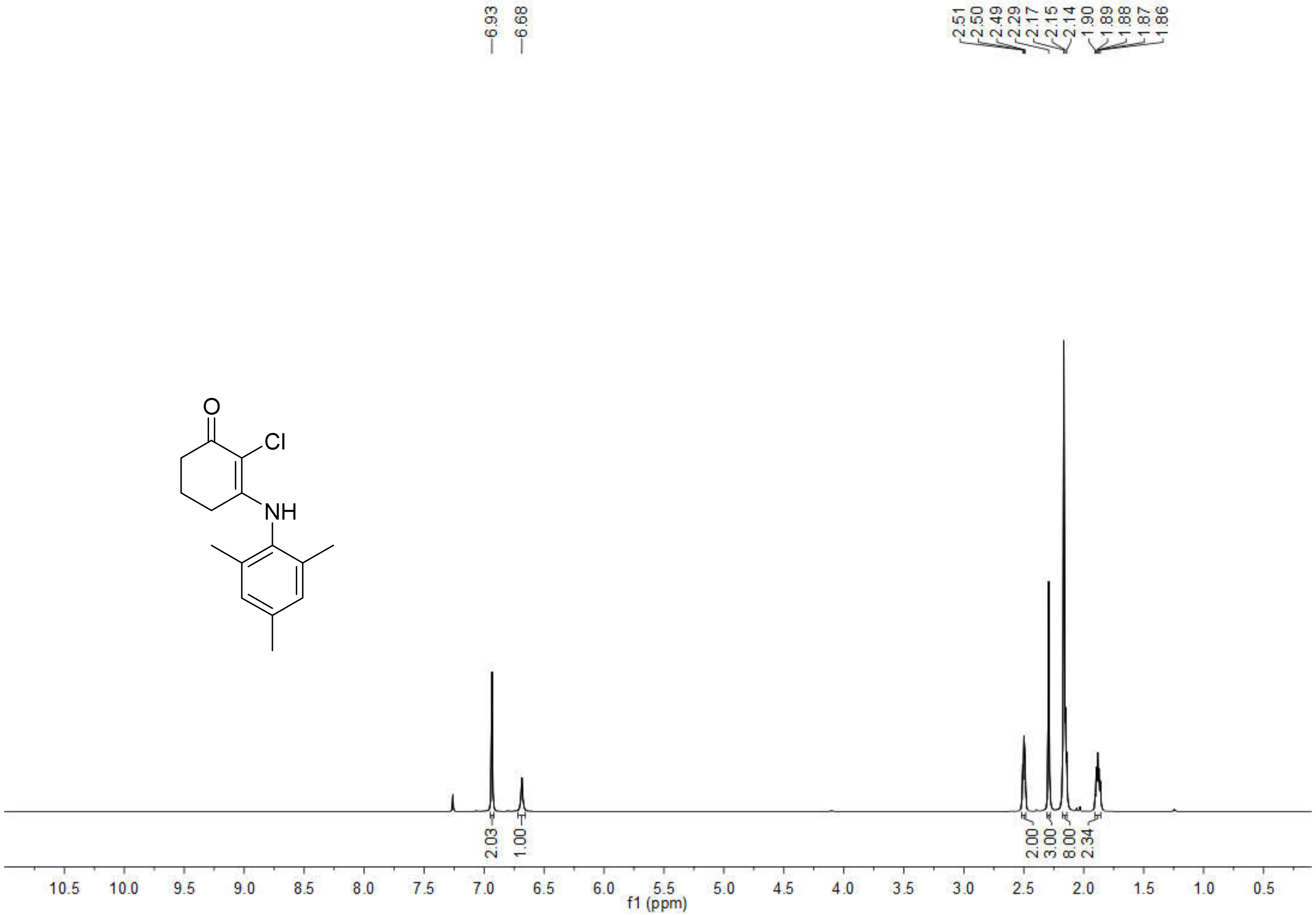

Figure S73. ^{1}H NMR (600 MHz, $CDCl_3$) spectra of compound **2x**

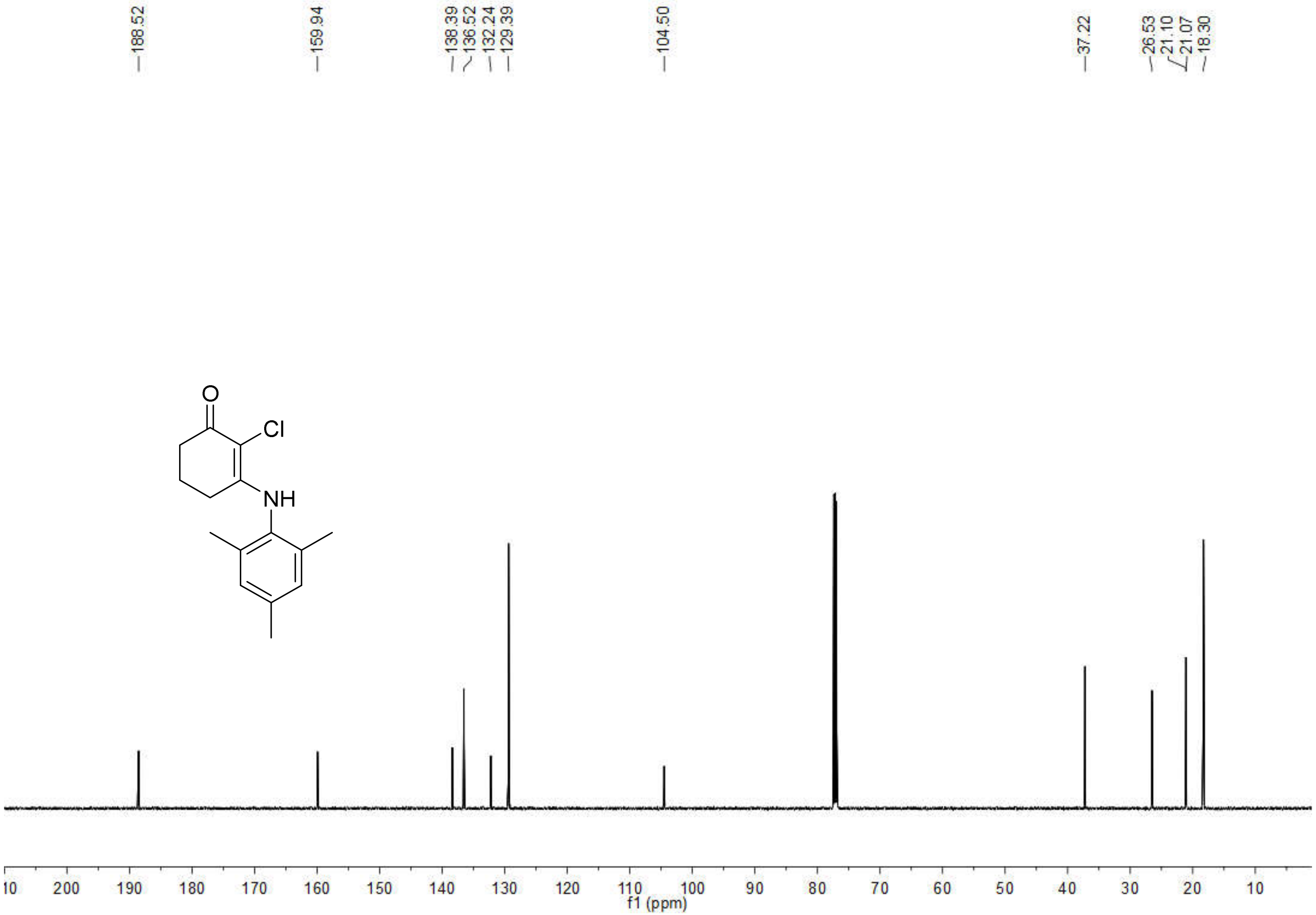

Figure S74. ^{13}C NMR (600 MHz, $CDCl_3$) spectra of compound **2x**

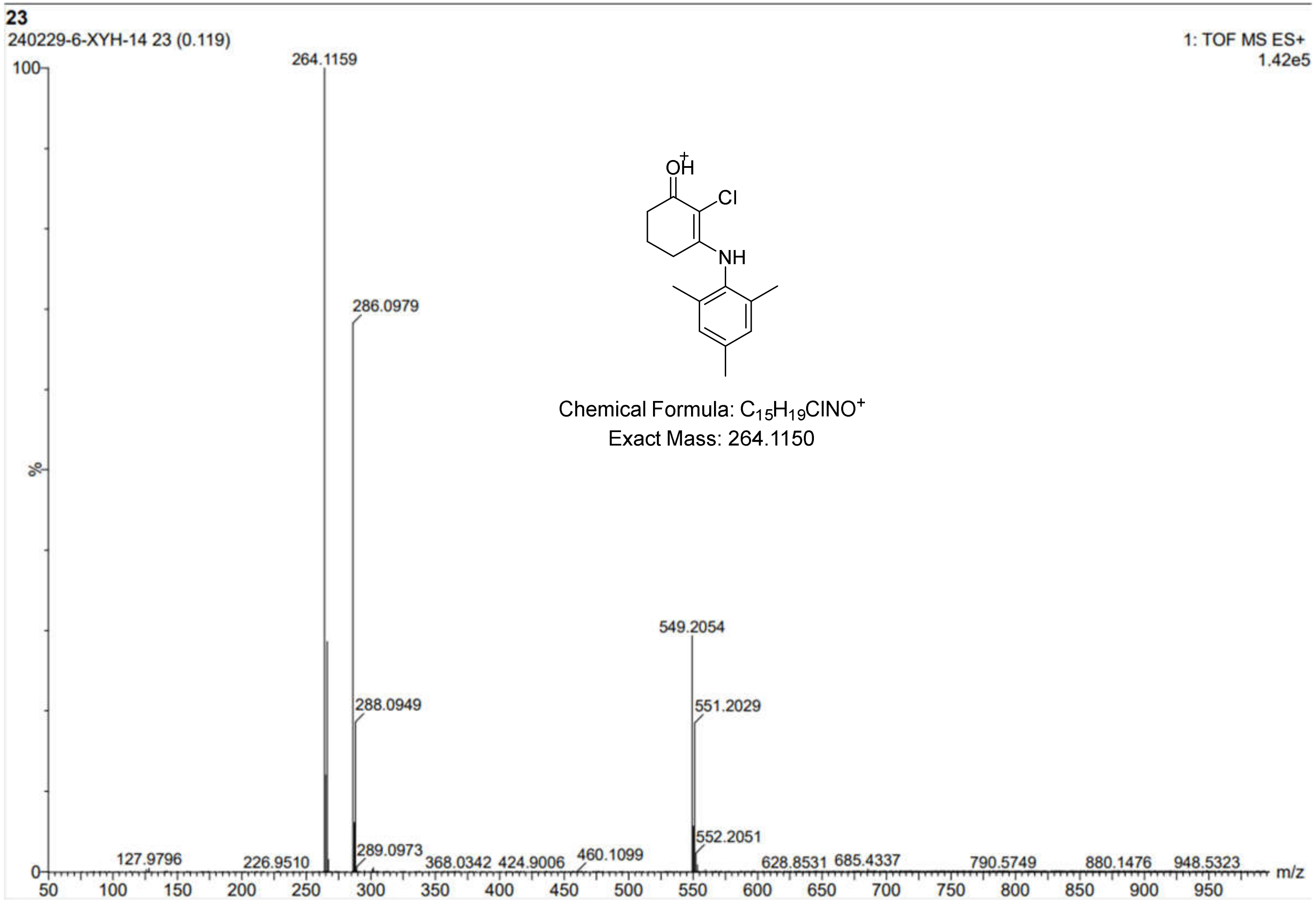

Figure S75. HRMS spectra of compound **2x**

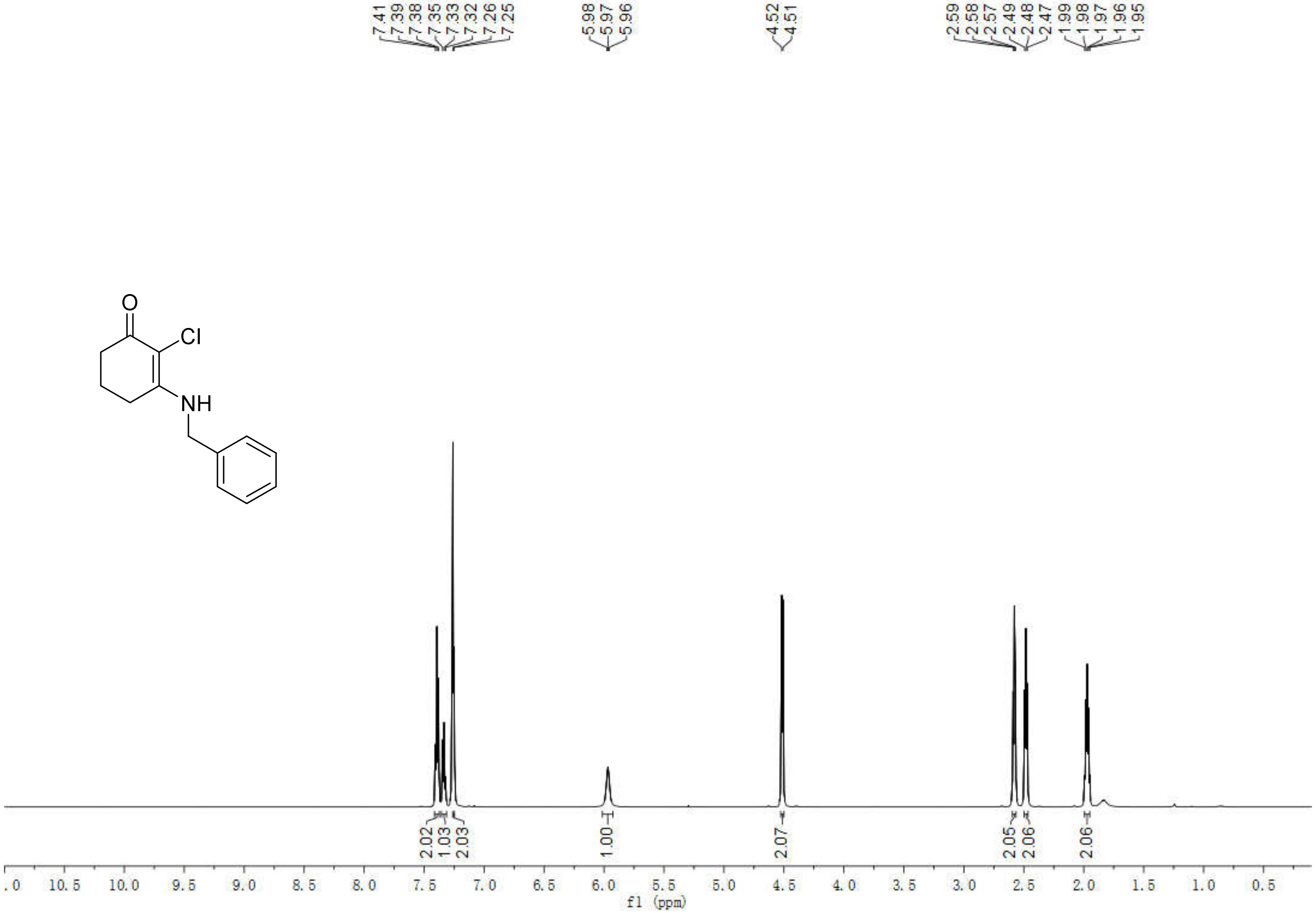

Figure S76. ^{1}H NMR (600 MHz, $CDCl_3$) spectra of compound **2y**

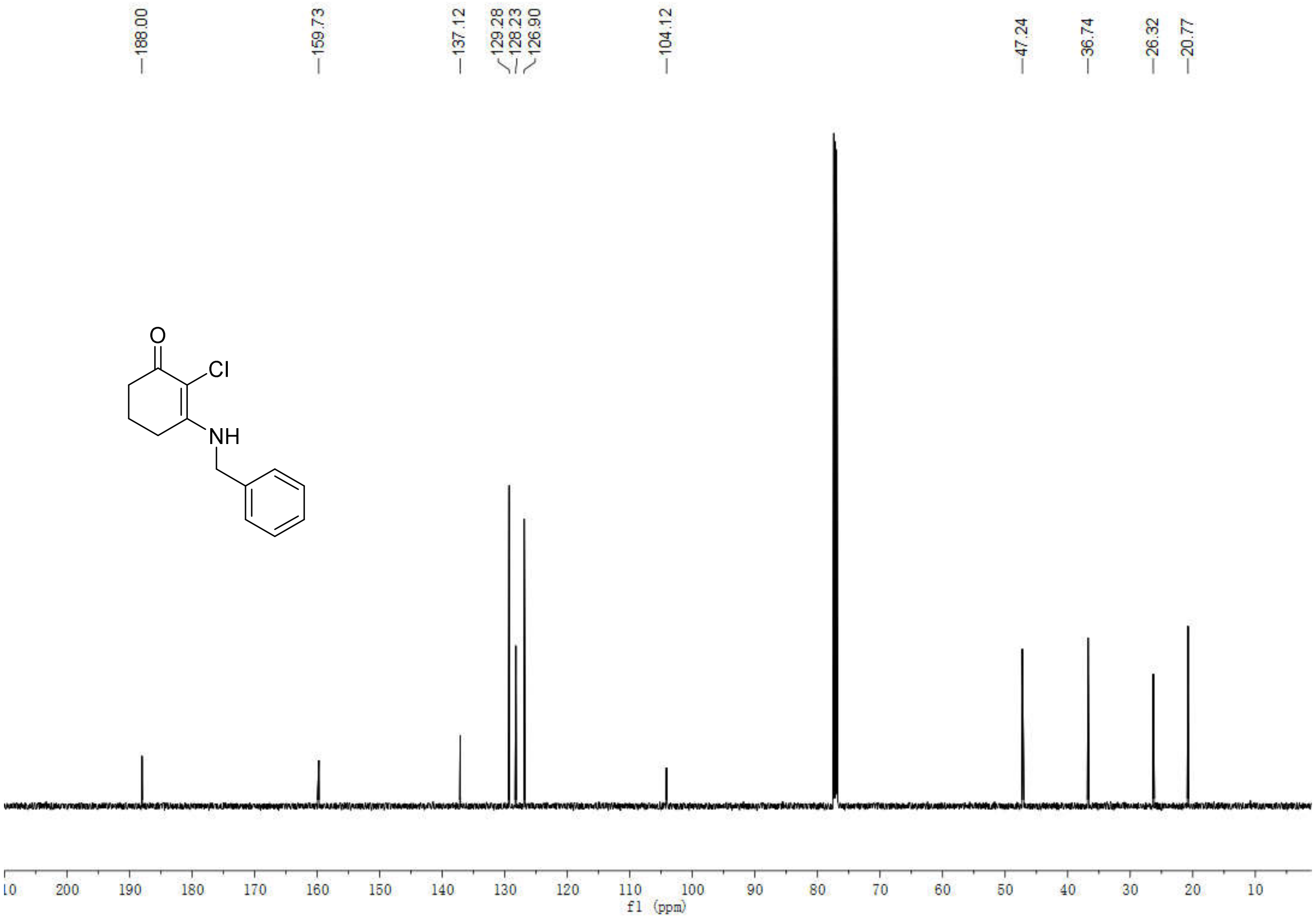

Figure S77. ^{13}C NMR (600 MHz, $CDCl_3$) spectra of compound **2y**

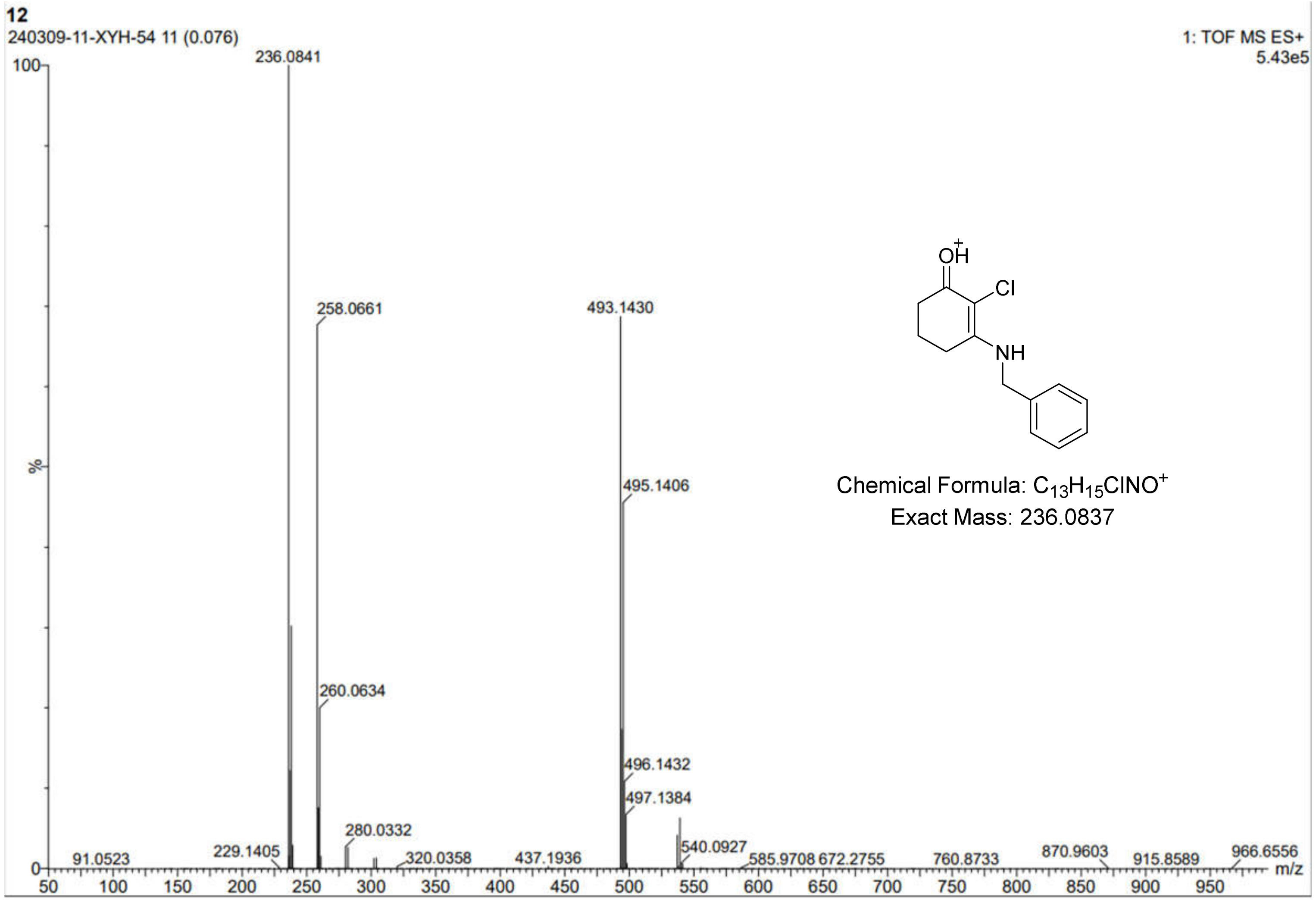

Figure S78. HRMS spectra of compound **2y**

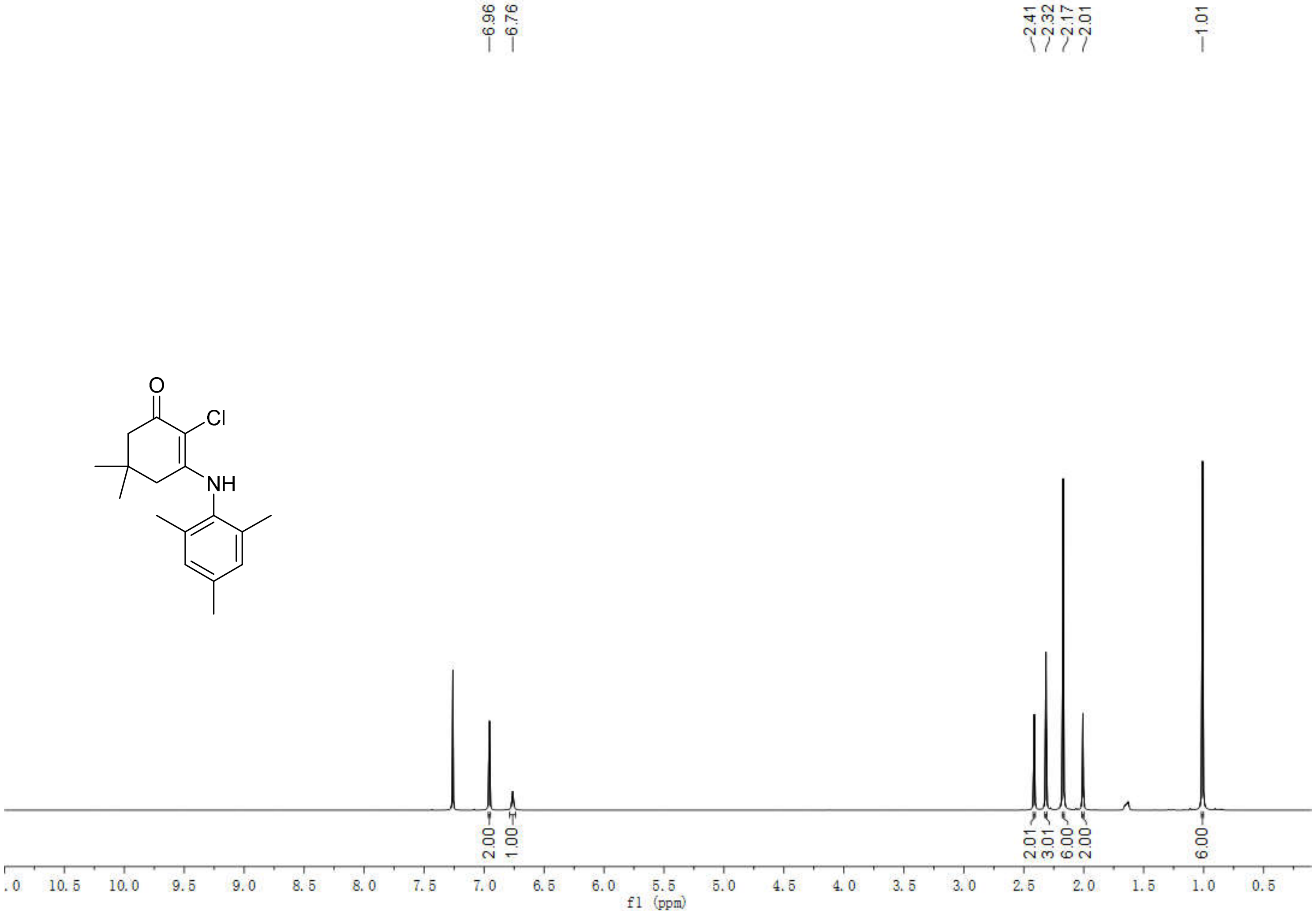

Figure S79. ^{1}H NMR (600 MHz, $CDCl_3$) spectra of compound **2z**

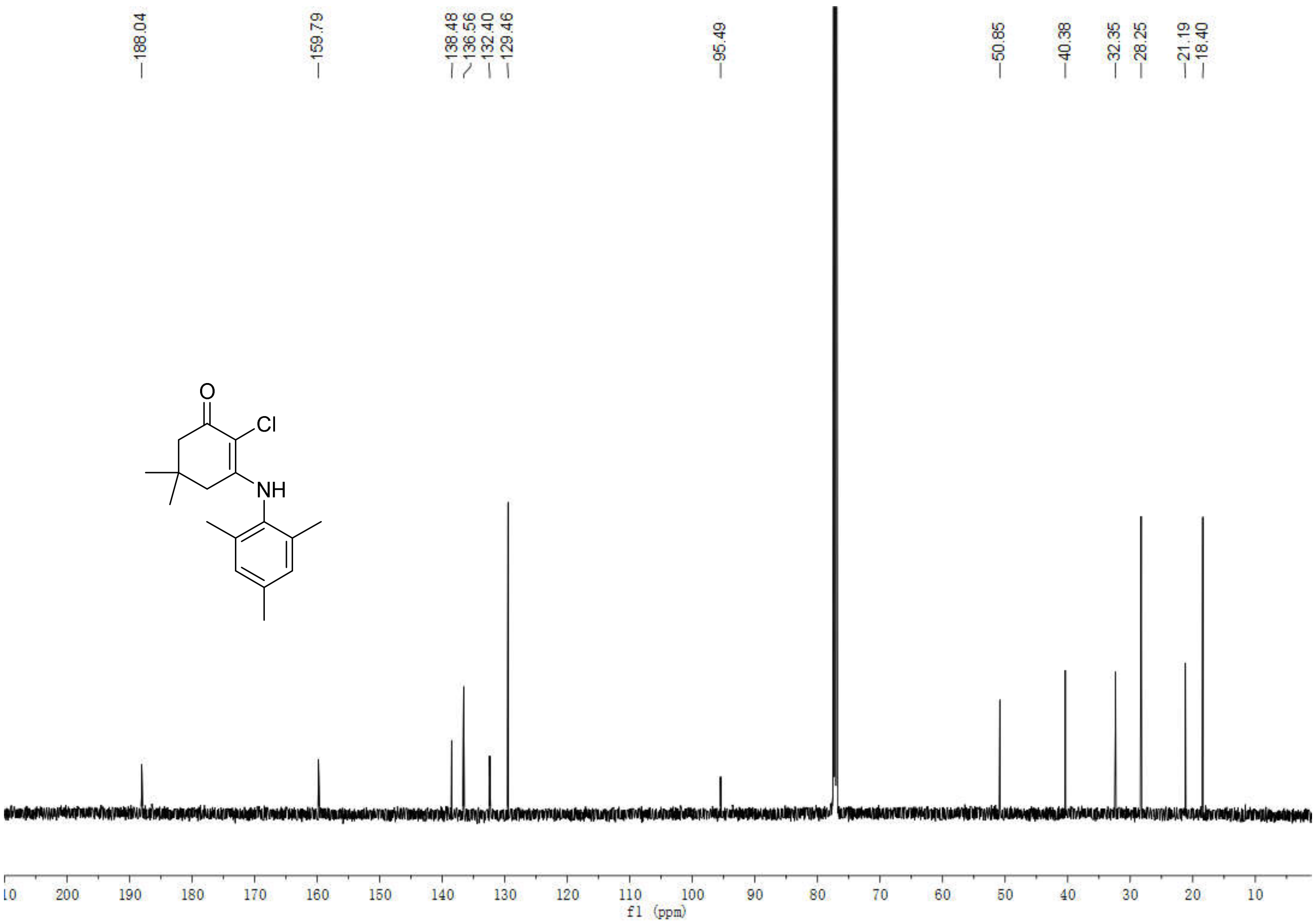

Figure S80. ^{13}C NMR (600 MHz, $CDCl_3$) spectra of compound **2z**

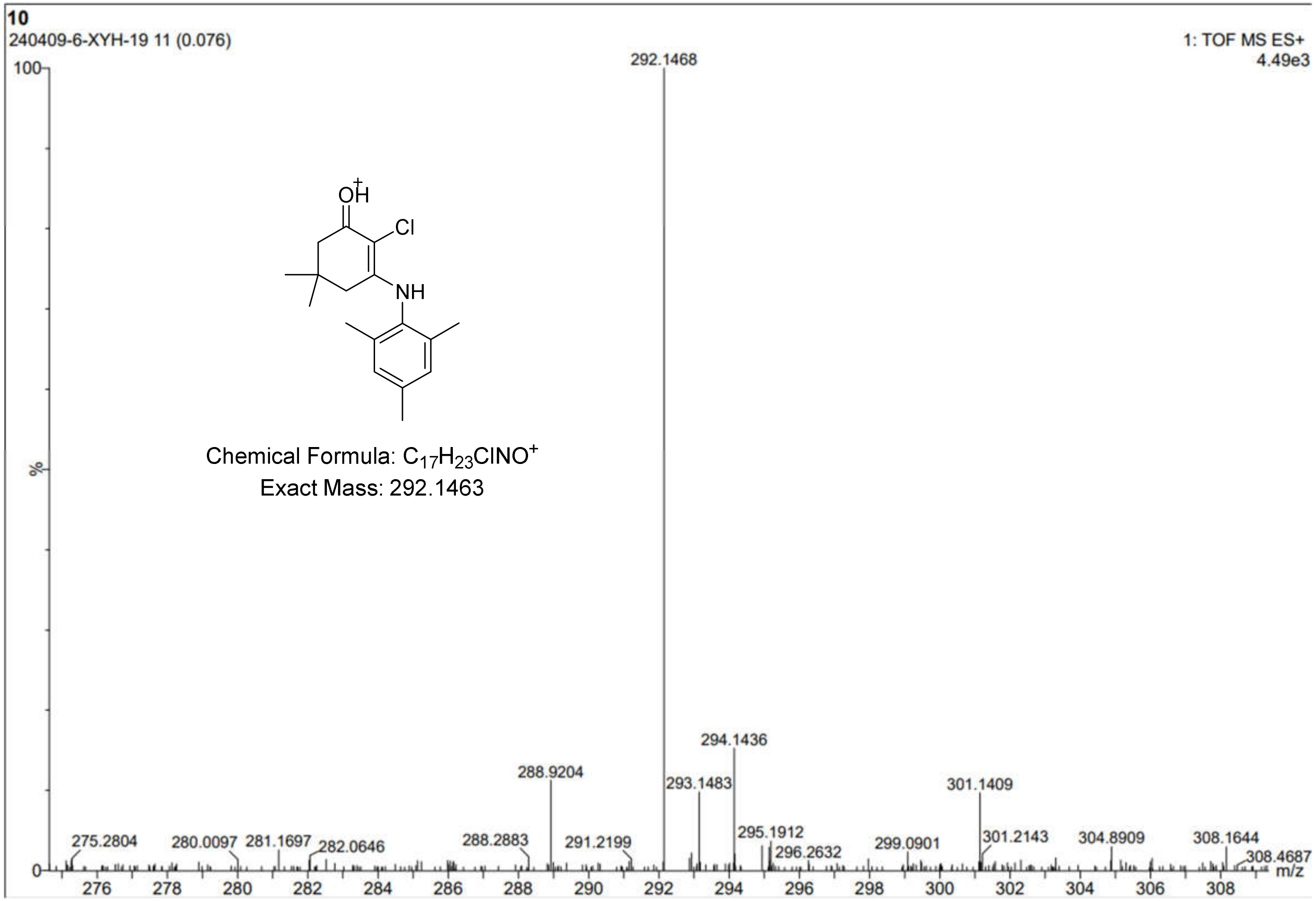

Figure S81. HRMS spectra of compound **2z**

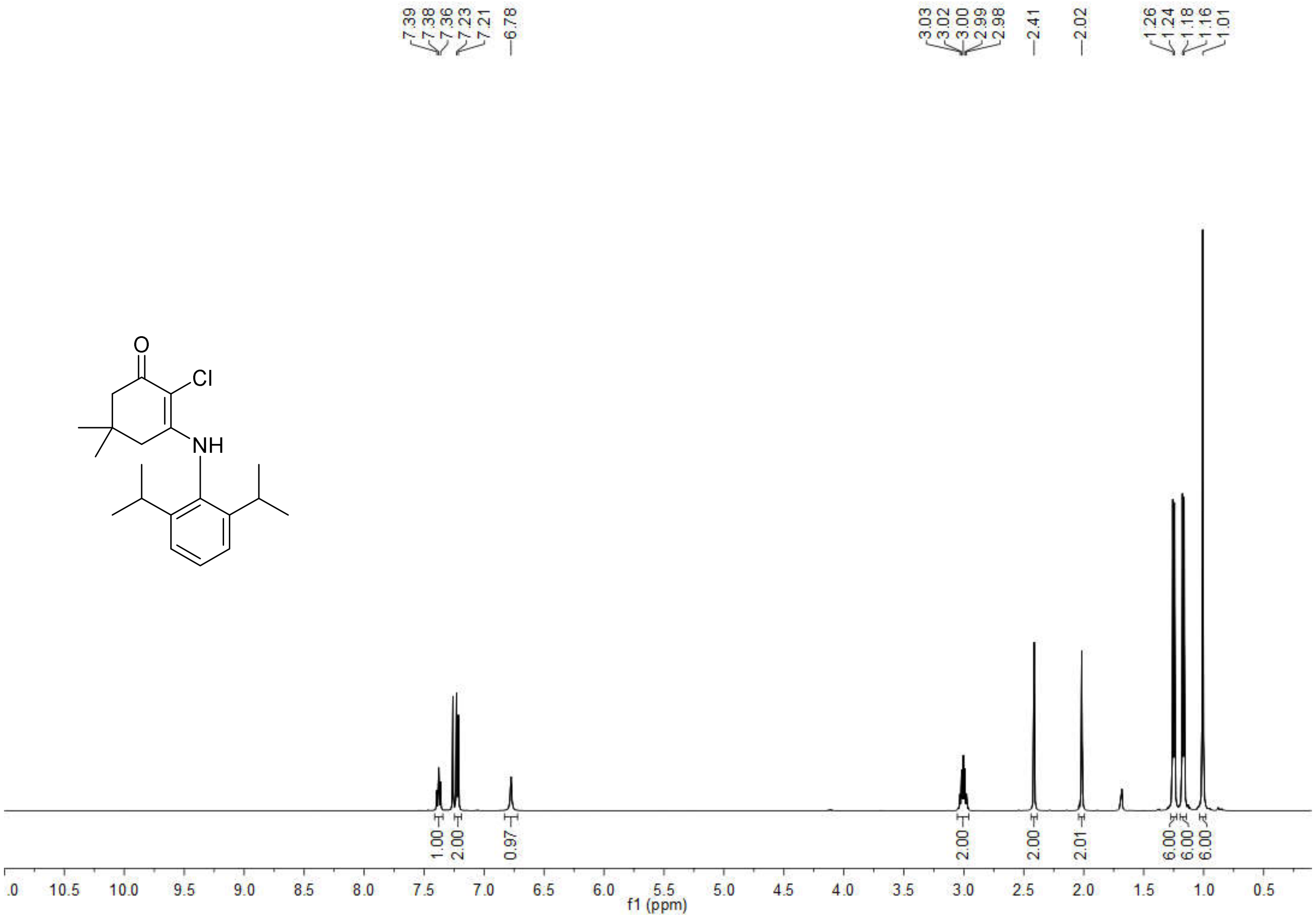

Figure S82. ^{1}H NMR (500 MHz, $CDCl_3$) spectra of compound **2a'**

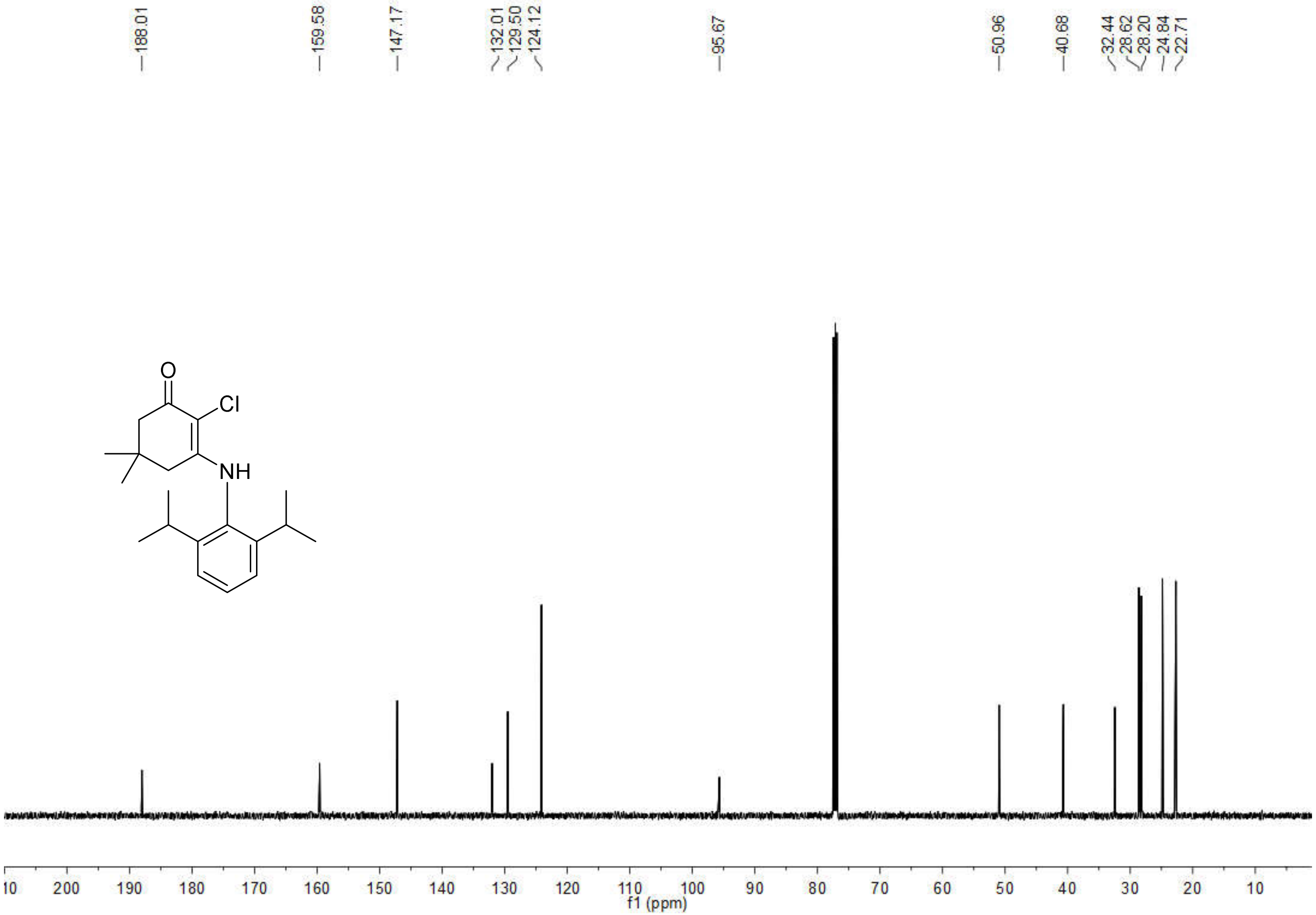

Figure S83. ^{13}C NMR (500 MHz, $CDCl_3$) spectra of compound **2a'**

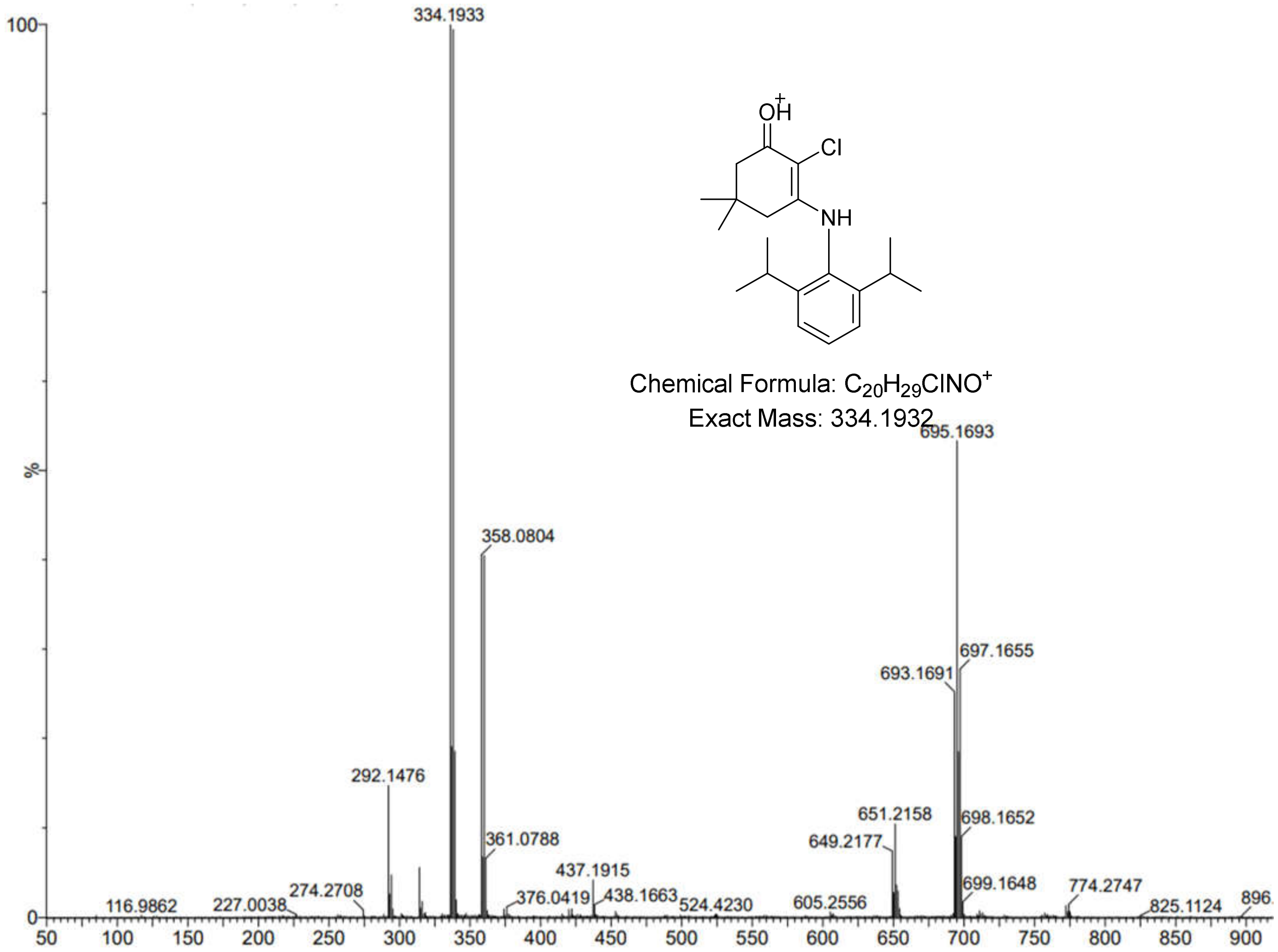

Figure S84. HRMS spectra of compound **2a'**

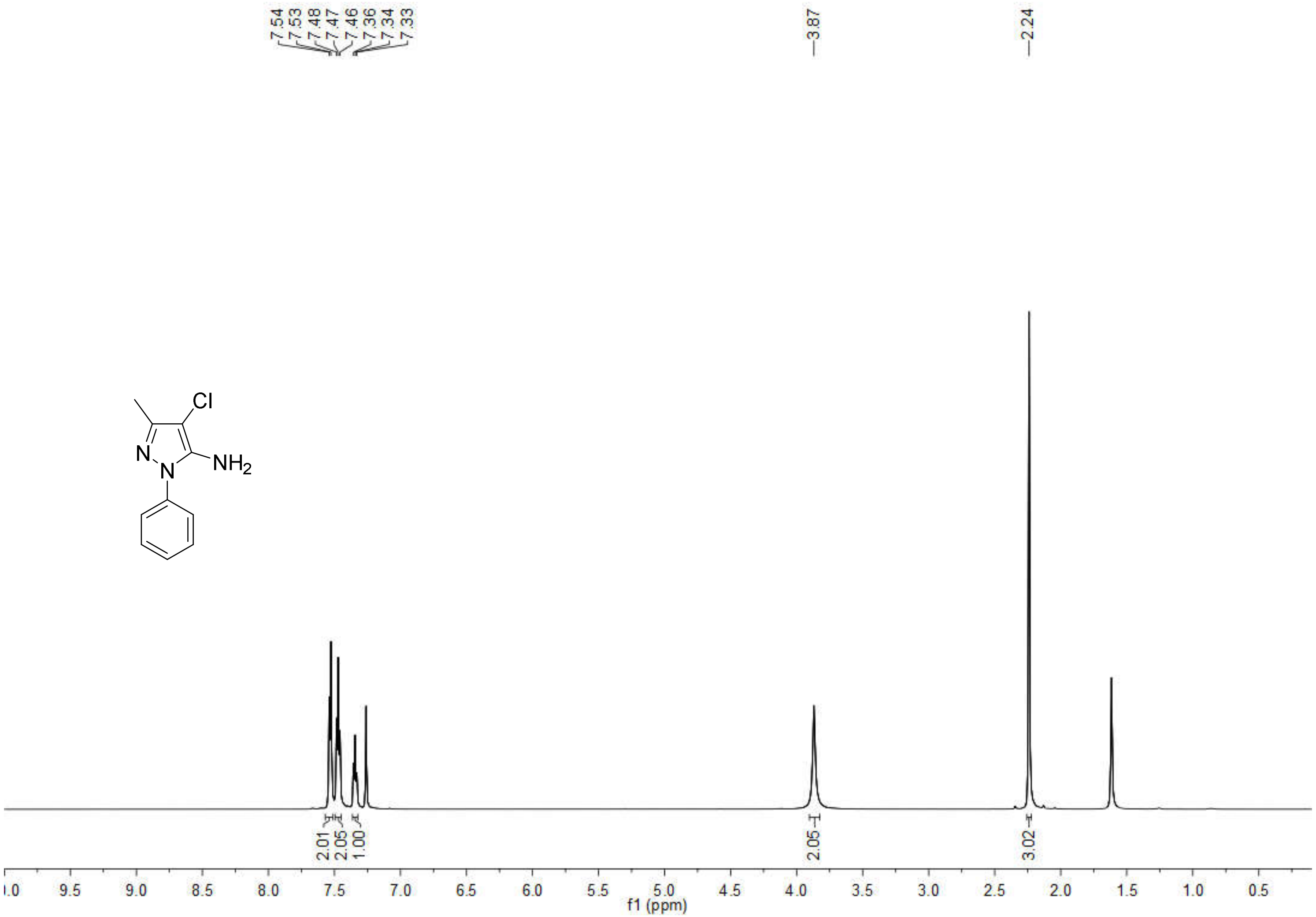

Figure S85. ^{1}H NMR (600 MHz, $CDCl_3$) spectra of compound **2b’**

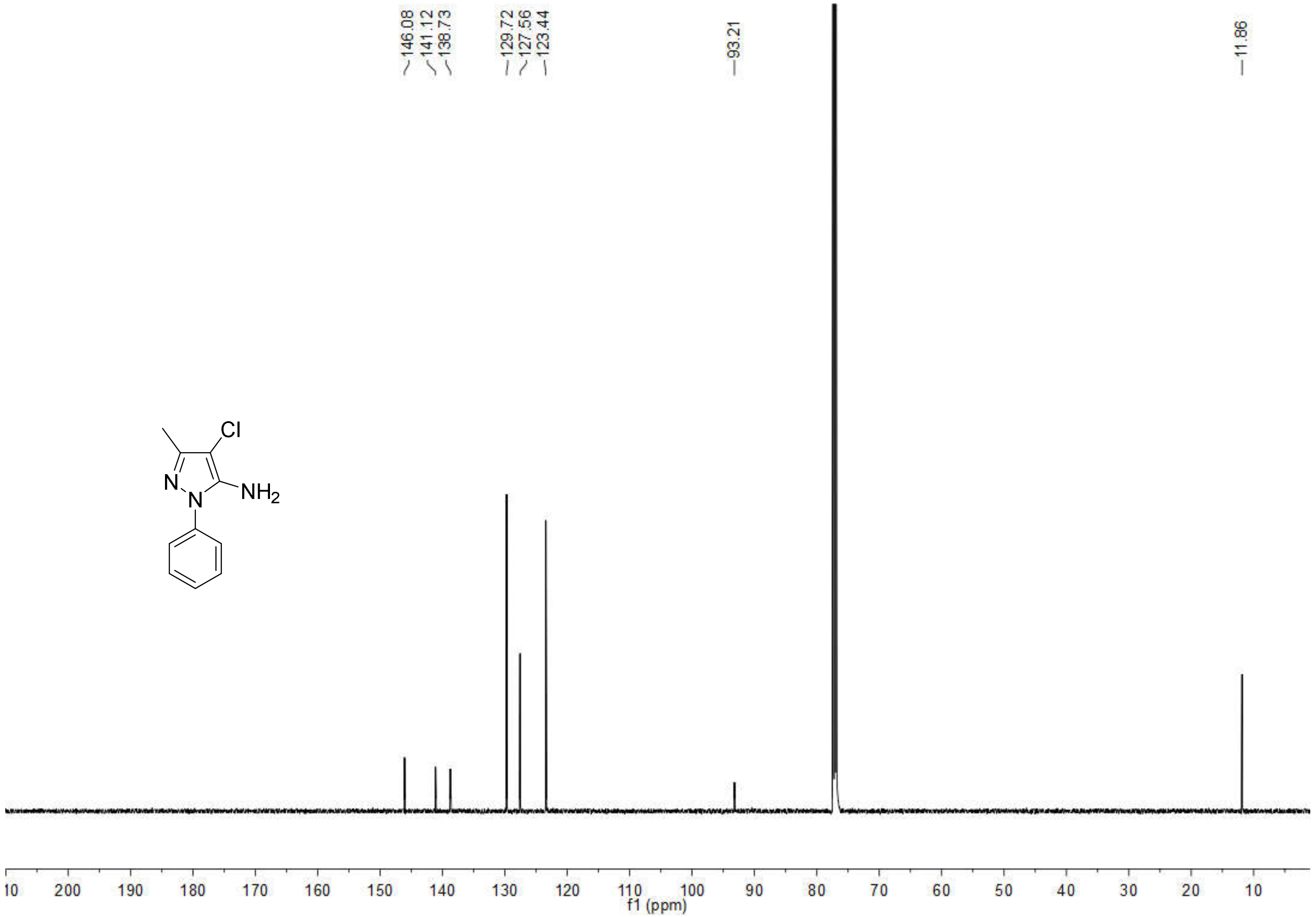

Figure S86. ^{1}H NMR (600 MHz, $CDCl_3$) spectra of compound **2b'**

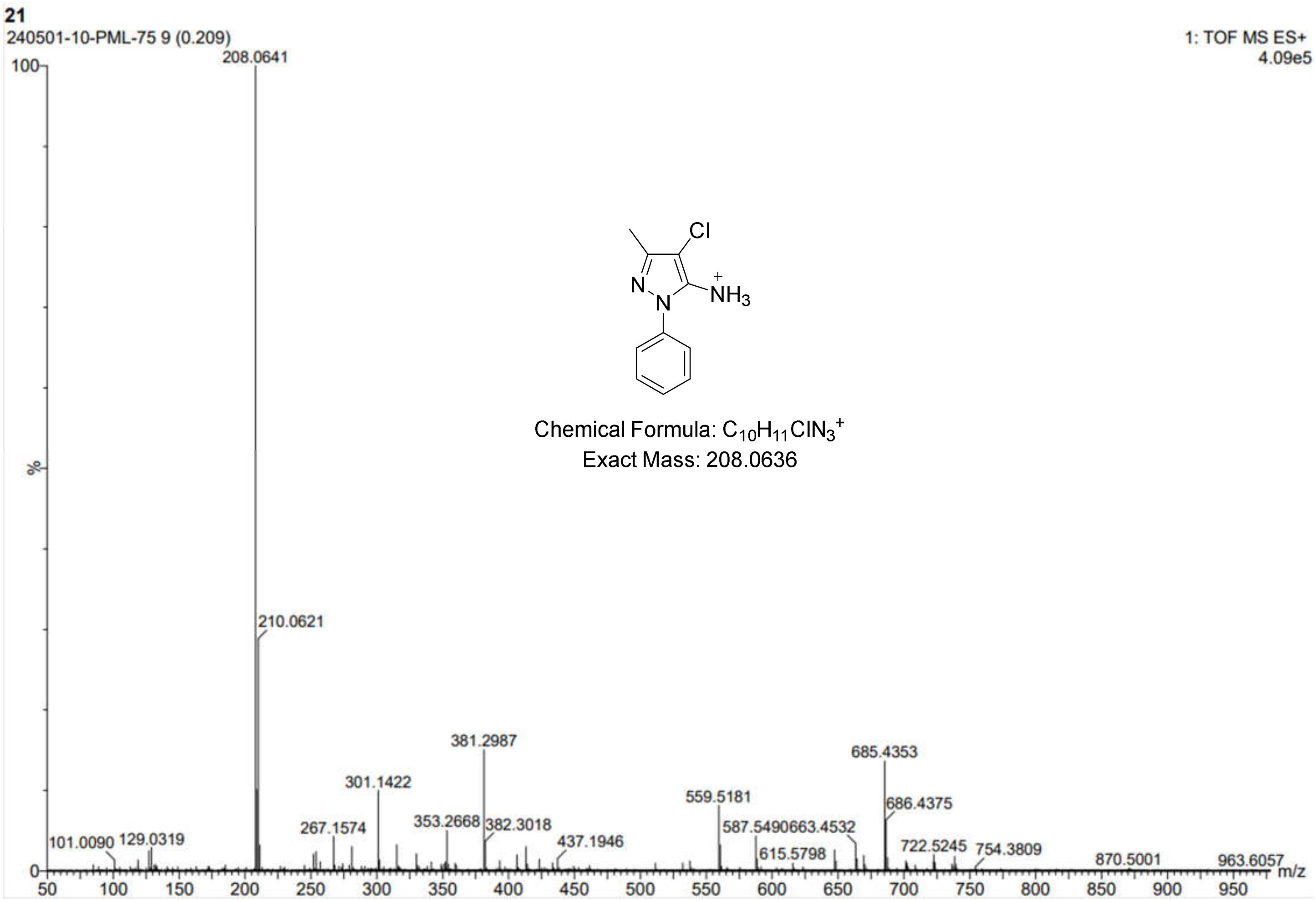

Figure S87. HRMS spectra of compound **2a’**

4. References and notes.

1. CCDC 2214194 contain the supplementary crystallographic data for compound **2m**. These data can be obtained free of charge from The Cambridge Crystallographic Data Center *via* www.ccdc.cam.ac.uk/data_request/cif.
